# Expanding the Utility of β-Diketiminate Ligands in Heavy Group VI Chemistry of Molybdenum and Tungsten

**DOI:** 10.1021/acs.organomet.3c00056

**Published:** 2023-06-01

**Authors:** Daniel Leitner, Benjamin Wittwer, Florian R. Neururer, Michael Seidl, Klaus Wurst, Frank Tambornino, Stephan Hohloch

**Affiliations:** † Faculty of Chemistry and Pharmacy, Institute for General, Inorganic and Theoretical Chemistry, University of Innsbruck, Innrain 80−82, Innsbruck 6020 Austria; ‡ Fachbereich Chemie and Wissenschaftlichen Zentrum für Materialwissenschaften (WZMW), Phillips-University Marburg, Hans-Meerwein-Straße 4, 35043 Marburg, Germany

## Abstract

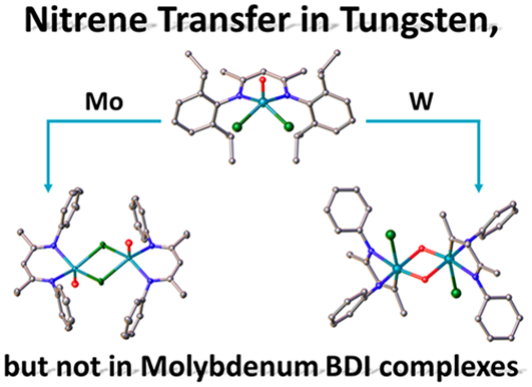

We report the synthesis of 17 molybdenum and tungsten complexes supported by the ubiquitous BDI ligand framework (BDI = β-diketiminate). The focal entry point is the synthesis of four molybdenum and tungsten(V) BDI complexes of the general formula [MO(BDI^R^)Cl_2_] [M = Mo, R = Dipp (**1**); M = W, R = Dipp (**2**); M = Mo, R = Mes (**3**); M = W, R = Mes (**4**)] synthesized by the reaction between MoOCl_3_(THF)_2_ or WOCl_3_(THF)_2_ and LiBDI^R^. Reactivity studies show that the BDI^Dipp^ complexes are excellent precursors toward adduct formation, reacting smoothly with dimethylaminopyridine (DMAP) and triethylphosphine oxide (OPEt_3_). No reaction with small phosphines has been observed, strongly contrasting the chemistry of previously reported rhenium(V) complexes. Additionally, the complexes **1** and **2** are good precursors for salt metathesis reactions. While **1** can be chemically reduced to the first stable example of a Mo(IV) BDI complex **15**, reduction of **2** resulted in degradation of the BDI ligand via a nitrene transfer reaction, leading to MAD (4-((2,6-diisopropylphenyl)imino)pent-2-enide) supported tungsten(V) and tungsten(VI) complexes **16** and **17**. All reported complexes have been thoroughly studied by VT-NMR and (heteronuclear) NMR spectroscopy, as well as UV–vis and EPR spectroscopy, IR spectroscopy, and X-ray diffraction analysis.

## Introduction

**Figure 1:**
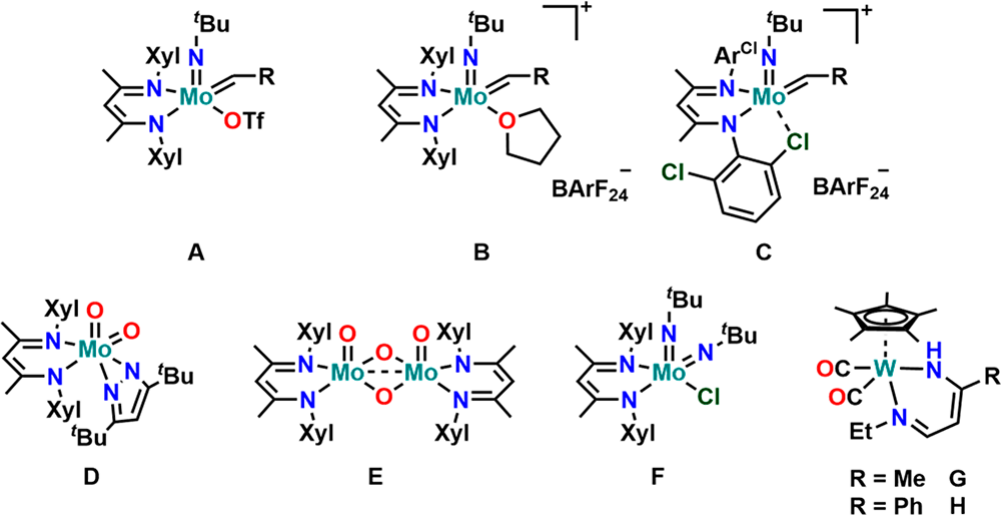
Overview of previously reported BDI complexes of molybdenum by Mösch-Zanetti (A–C)^86^ and Schrock (D–F)^84,85^ and the sole tungsten complex (G and H) by Filippou et al.^87^

β-Diketiminate (BDI) ligands are among the most explored ligands in modern organometallic and coordination chemistry.^1−3^ Due to their unique combination of simple synthetic accessibility, following a facile but highly modular condensation protocol, and their steric and electronic tunability,^4,5^ these ligands have played a vital role in the isolation of long-sought intermediates and highly reactive species. Thus, these ligands have found widespread applications all over the periodic table spanning from main group chemistry, covering both s-^6−11^ and p-block^12−14^ elements, to d-block transition metal chemistry,^15−23^ culminating in f-block chemistry.^24−28^ Focusing on their chemistry with the early transition metals,^29^ these breakthroughs included the isolation of a plethora of reactive metal–ligand multiple bonds. Mindiola and co-workers for example reported the isolation of titanium nitrido^30,31^ and phosphinidene^32,33^ complexes or a vanadium phosphinidene complex,^34^ while Arnold and co-workers recently explored the versatile chemistry of niobium^35−43^ and rhenium BDI complexes.^44−48^ The latter included a rare example of a noncarbonyl stabilized rhenium(I) cyclopentadiene complex,^49,50^ which has also been proven to be a potent candidate for the formation of new metal–metal bonds, e.g., a Re–Zn–Zn–Re complex,^51^ or Cp bridged tetranuclear actinide complexes.^52^ Apart from that, a plethora of catalytic reactions have been elucidated using BDI supported early transition metal complexes, e.g., nitrene transfer reactions to yield carbodiimides^40^ or hydrodefluorinations^39,41,53^ both catalyzed via low-valent niobium(III)^39−41^ or titanium(III)^53^ complexes. However, looking at the numerous explorations of the BDI ligand framework in group IV,^30−33,54−58^ group V,^29,34,37,42,43,59−61^ and group VII^44−49,51,52,62−69^ metal chemistry, group VI metals (except for chromium^70−83^) have been mostly neglected in this research. Hitherto, there are only three literature reports on BDI supported molybdenum complexes: two on imido alkylidene complexes by Schrock^84,85^ (Figure 1 A–C) and one covering the synthesis of dioxo and bis-imido complexes (Figure 1D–F) by Mösch-Zanetti.^86^ Turning to tungsten, only two β-diiminato complexes (Figure 1G,H) have been reported so far by Filippou and co-workers.^87^ However, these do not form via the direct installation of a BDI ligand on a tungsten complex, but by the addition of an organic nitrile onto a Cp-supported aza-allyl tungsten(II) complex. Given the fundamental meaning of molybdenum and tungsten in catalysis^88−98^ and small molecule activation,^99,100^ especially nitrogen,^101−105^ the use of BDI ligands might be interesting to develop new paths in the chemistry of these elements.

Our group has recently started to explore the chemistry of redox-active dipyrrin ligands in high-valent molybdenum bis-imido chemistry.^106^ Simultaneously, Betley and co-workers reported high-valent chromium (bis-)imido complexes,^107^ generated through the redox-versatility of low-valent chromium dipyrrin complexes. In both these reports it becomes obvious that dipyrrin ligands seem to mimic the coordination chemistry of BDI ligands very well^108^ but offer larger potential for further applications of these systems, e.g., in photo- and redox-chemistry and suppressing catalyst deactivation pathways. However, given the lack of BDI reference complexes discussed above, we aim to expand our investigations toward the scope and limitations of BDI ligands to be able to further explore the differences and similarities between these two important ligand types in early transition metal chemistry.

## Results and Discussion

**Scheme 1:**
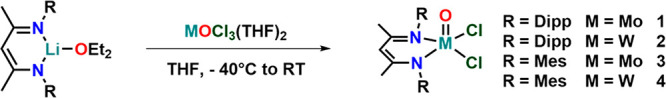
Synthesis of Molybdenum(V) and Tungsten(V) BDI Complexes with Variable Steric Demand

**Figure 2:**
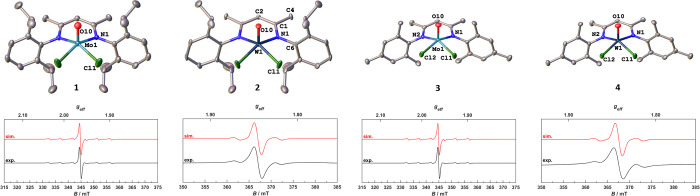
Molecular structures of the monometallic molybdenum(V) and tungsten(V) complexes **1**–**4**. Solvent molecules and hydrogen atoms have been omitted for clarity. Ellipsoids are shown at a probability level of 50% (top). EPR spectra of all complexes measured at 298 K (black line) and simulated (red line) (bottom).

Given the observed preference of the +V oxidation state over +VI in molybdenum BDI complexes,^86^ we investigated the less Lewis-acidic molybdenum(V) and tungsten(V) mono-oxo complexes. Synthesis was achieved by mixing LiBDI^R^ (R = Dipp, Mes) and the oxo trichloride precursors MOCl_3_(THF)_2_ (M = Mo, W) in THF at −40 °C and let proceed at RT overnight. This procedure gave access to complexes **1**–**4** (Scheme 1) in yields of 58–88% and is scalable to up to 10 g for **1** and **2**. Due to the inherent paramagnetism of the complexes, no characteristic NMR signals could be observed (Figure S1–S4). The Evans method revealed magnetic moments of 1.75, 1.30, 1.61, and 1.37 μ_B_ for **1**–**4** respectively, which is in line with a d^1^ configuration. Furthermore, the EPR spectra of all complexes agree with metal centered S = 1/2 spin systems (Figure 2, bottom) and revealed *g*_iso_-values of 1.963 (*a*_iso_ = 130 MHz), 1.844 (*a*_iso_ = 244 MHz), 1.962 (*a*_iso_ = 130 MHz), and 1.841 (*a*_iso_ = 249 MHz) for **1**–**4**, respectively. X-ray quality crystals were grown from concentrated diethyl ether solutions for all four complexes. The complexes **1** and **2**, as well as the complexes **3** and **4** crystallize isostructurally in the orthorhombic space group *Pnma* with half a molecule in the asymmetric unit (for **1** and **2**) or the triclinic space group *P*1̅ with a full molecule in the asymmetric unit (for **3** and **4**). In all complexes, the metal center exhibits a square pyramidal coordination geometry, displaying τ_5_ values of 0.00 for **1** and **2** and 0.13/0.12 for **3** and **4** respectively. The BDI ligand is symmetrically bound to the metal center, displaying similar metal nitrogen distances of 2.090(3) and 2.100(3) Å in complex **1** and **2** and 2.097(2)/2.089(2) and 2.084(2)/2.088(3) Å in complex **3** and **4**. This similarity in bond lengths between the molybdenum and the tungsten complexes can also be found in the M1–O10 distances lying at 1.640(4), 1.693(4), 1.657(2), and 1.681(2) Å in **1**–**4** and is a direct result of the lanthanide contraction and similar ionic radii of Mo^V^ and W^V^. Overall, the structural parameters are also comparable to the recently studied BDI complexes of niobium(V) and rhenium(V).^29,39,44,60^ For further structural parameters, please refer to the Supporting Information, Table S1–S3.

**Scheme 2:**
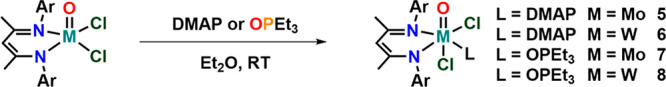
Reactivity between Complexes **1** and **2** and Neutral Lewis Bases Ar = Dipp.

**Figure 3:**
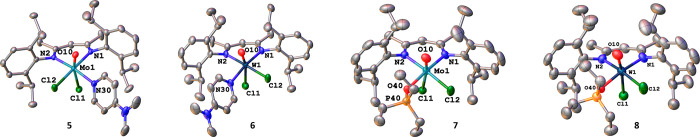
Molecular structures of the molybdenum and tungsten DMAP and OPEt_3_ adducts **5**–**8**. Hydrogen atoms and lattice solvent molecules have been omitted for clarity. Ellipsoids are shown at a probability level of 50%.

Considering the fact that the BDI^Dipp^ complexes **1** and **2** can be accessed more conveniently and in higher yields, we examined their further reactivity. Initial focus was laid on their reactivity toward neutral donor systems such as DMAP, triethylphosphine oxide (OPEt_3_), isocyanides, and small phosphines (PEt_3_ or PMe_3_). Upon mixing solutions of **1** or **2** with DMAP or OPEt_3_ in diethyl ether an instant color change of the solution was observed (see Experimental Section) concomitant with the formation of a reddish precipitate. The precipitate was filtered off, but unfortunately due to its low solubility no further characterization of this material was possible. The remaining solutions were evaporated and gave access to the DMAP (**5** and **6**) and OPEt_3_ adducts (**7** and **8**) in yields between 33% and 60% (Scheme 2). As expected, all complexes are paramagnetic (Figure S5–S8) displaying effective magnetic moments ranging from 1.51 to 1.82 μ_B_ indicative for a d^1^ configured metal center. Room temperature EPR spectra also showed the expected signals for an unpaired electron located at the respective molybdenum and tungsten centers (Figure S52–S55). X-ray quality crystals of the adducts **5**–**8** were grown from concentrated diethyl ether solutions at room temperature over 2 days. The corresponding molybdenum and tungsten adducts crystallize isostructurally in the monoclinic space groups *P*2_1_/*n* for **5** and **6** and *C*2/*c* for **7** and **8** with one molecule of the respective complex in the asymmetric unit. All complexes display a distorted octahedral environment around the metal center with the *trans* O10–M1–Cl1 angles being 165.62(7)°, 167.43(14)°, 171.97(6)°, and 172.95(12)° in **5**, **6**, **7**, and **8** (Figure 3). Notably, Cl1 oriented *trans* to the oxo ligand experiences a strong *trans*-influence, as its distance to the metal center increases by 0.06–0.1 Å to 2.453(3), 2.4594(16), 2.4945(6) and 2.4946(13) Å compared to 2.383(2), 2.3753(14), 2.3946(6), 2.3935(13) Å for M1–Cl2 for **5**, **6**, **7** and **8** respectively. The additional donor ligands are located *trans* to a BDI nitrogen donor and *cis* to the oxo ligand (Figure 3) and display distances of 2.256(3) and 2.237(5) Å for M1–N30 in the DMAP adducts **5** and **6**, and M1–O40 distances of 2.1117(15) and 2.104(3) Å in the triethylphosphine oxide adducts **7** and **8**. The substantially contracted M1–O40 distances (shorter by ca. 0.1 Å) in **7** and **8** compared to the M1–N30 distances in **5** and **6** is in line with OPEt_3_ being the stronger donor, as it would be expected for oxophilic transition metals. Similar conformations have also been reported for rhenium oxo BDI adducts.^44^

In contrast, no defined reaction took place between complexes **1** and **2** and xylyl or *tert*-butyl isocyanides. Instead, complex reaction mixtures were observed employing different reaction conditions. Also, with triethylphosphine (PEt_3_) no reaction was observed. Both are surprising, since the corresponding niobium^38,109^ and rhenium^44,110^ adducts both have been reported. Furthermore, heating samples containing PEt_3_ to 100 °C did not facilitate any visible amount of OAT (oxygen atom transfer) reactivity in these systems (see Figure S24 for NMR monitoring of the reaction between **1** and PEt_3_), contrasting the rhenium case.^44^ The inertness of complexes **1** and **2** toward small phosphines is further surprising, since other molybdenum complexes can be easily deoxygenated under similar conditions.^95,111^ A possible explanation for this could be, that the deoxygenation of **1** and **2** would result in a corresponding M(III) oxidation state, which is highly reducing for both, molybdenum and tungsten. Thus, it is possible that stronger reductants (stronger oxygen atom acceptors) are needed to promote this reaction.

**Scheme 3:**
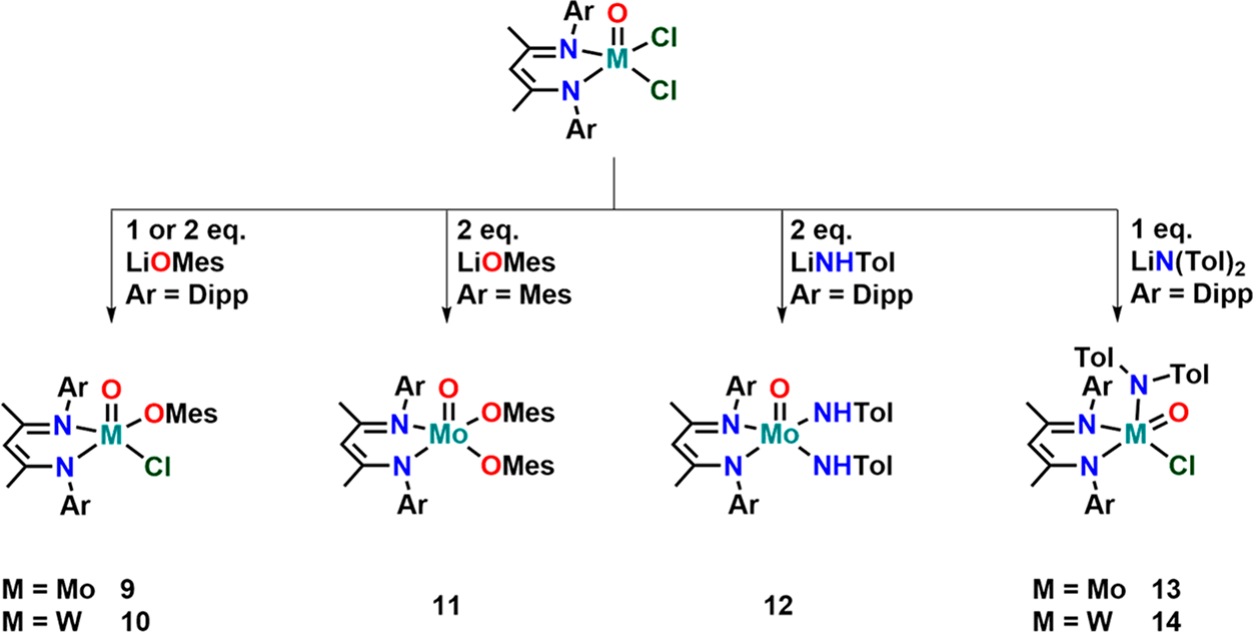
Salt Metathesis Reactivity between Complexes **1**, **2**, and **3** with Mesitolate, Tolyl-amide, and Bis-tolyl-amide

**Figure 4:**
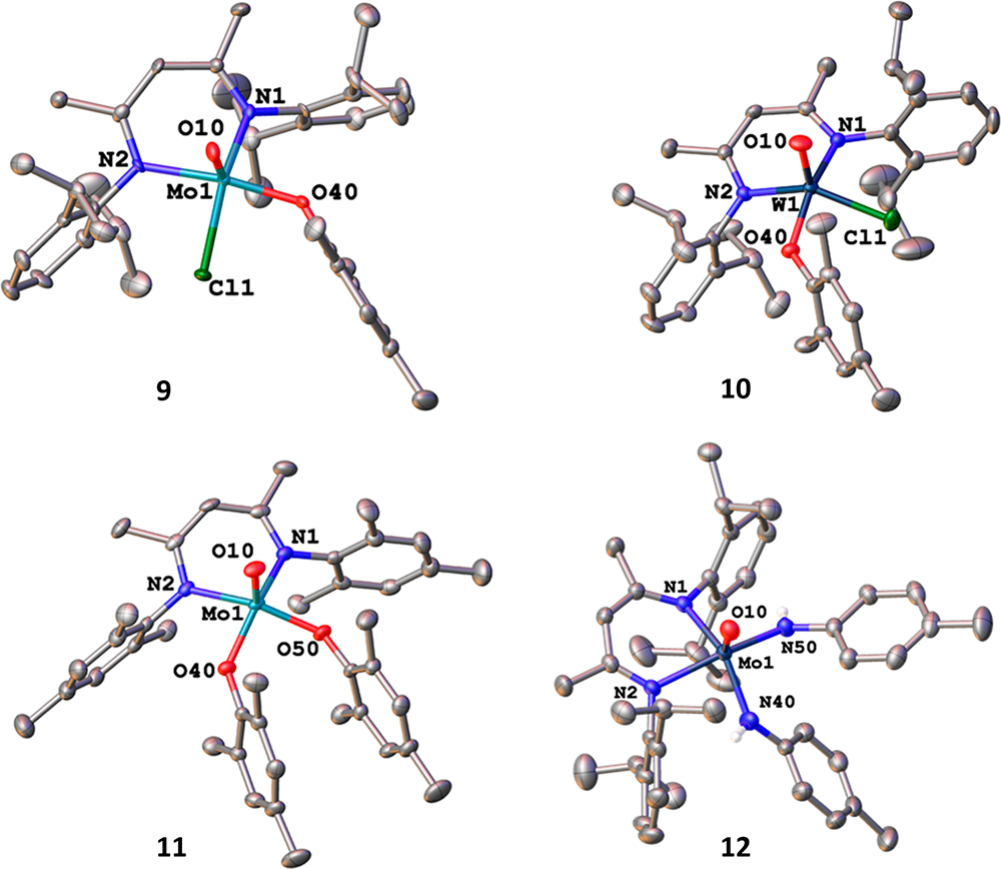
Molecular structures of the mesitolate complexes **9**–**11** and the bis-*p*-tolyl-amide complex **12**. Hydrogen atoms and solvent lattice molecules have been omitted for clarity. Ellipsoids are shown at a probability level of 50%.

Next, salt metathesis reactions, with complexes **1** and **2** were investigated, aiming toward the formation of more complex and reactive structures (Scheme 3). Independent from the equivalents used (1 or 2), the reaction between **1** or **2** with lithium mesitolate (LiOMes) resulted in the formation of the monomesitolate complexes **9** and **10** (Scheme 3). Even under forcing conditions (50 °C overnight), it was not possible to replace the second chloride ion by an additional phenolate ligand. We assume that this is related to steric crowding caused by the BDI^Dipp^ supporting ligand. To support this hypothesis, we also performed the reaction with the BDI^Mes^ complex **3** (*vide infra*) and two equivalents of LiOMes. As expected, this led to the clean and direct isolation of the bis-mesitolate complex **11**. All mesitolate complexes display the expected features of a paramagnetic d^1^ configured metal center with magnetic moments of 1.57, 1.26, and 1.63 μ_B_ for **9**, **10**, and **11** (Figure S9–S11) as well as the expected metal centered EPR signals (Figure S56–S58). Unambiguous proof for the identity of the mesitolate complexes was provided by X-ray structure analysis. X-ray quality crystals of all complexes were grown from concentrated diethyl ether solutions over several days. Complex **8** crystallizes in the orthorhombic space group *Pbca*, complex **10** in the triclinic space group *P*1̅ and complex **11** in the monoclinic space group *P*2_1_/*n*, all with one molecule in the asymmetric unit. In all complexes, the metal center is penta-coordinated by the BDI ligand, the oxo ligand and the anionic mesitolate/chloride ligands. Notably, with a τ_5_ value of 0.30 and 0.24 for **9** and **10**, compared to 0.03 in **11** the monosubstituted complexes show a more distorted square-pyramidal environment around the metal center. However, this might not only be related to the monosubstitution but also to the higher steric congestion caused by the Dipp group in **9** and **10** compared to the Mes-substituents in **11**. At the same time, this “asymmetry” causes a minor distortion in the bond lengths toward the BDI ligands. While in the previous cases (**1**–**8**), the BDI-metal distances were symmetric (deviating only by 0.02 Å in average), for **9** and **10** differences up to 0.08 Å between M1–N1 and M1–N2 can be observed. The metal mesitolate distances are 1.932(4) in **9**, 1.938(3) in **10**, and 1.9390(16)/1.9356(16) in **11** and are thus in the range of previously reported molybdenum mesitolate complexes (Figure 4).^94^

Since mesitolate is sterically too crowded to be installed twice on the sterically more demanding complex **1** with the BDI^Dipp^ supporting ligand, we turned our focus to smaller nucleophiles. Indeed, when reducing the steric bulk of the coligands from mesityl to *p*-tolyl as in *p*-tolylamide, the clean formation of the corresponding bis-*p*-tolylamide complex **12** is observed. Similar to the mesitolate complexes discussed before, the complex exhibits a d^1^ configuration displaying an effective magnetic moment of 1.52 μ_B_ and a *g*-value of 1.962 (see Figure S12 and S59 for NMR and EPR). The EPR spectrum shows hyperfine coupling (*a*_iso_(H) = 17.2 MHz) to two NH protons, further supporting a successful salt metathesis reaction. Unambiguous proof for the formation of **12** was obtained by X-ray diffraction analysis from single crystals grown from concentrated pentane solutions within 10 min at room temperature. Similar to the other complexes the molybdenum center is penta-coordinated but due to the steric repulsion between the ligands, its geometry is in between of square pyramidal and trigonal bipyramidal displaying a τ_5_ value of 0.45. The molybdenum amido distances are 1.984(2) and 2.006(2) Å for Mo1–N40 and Mo1–N50. Thus, these are substantially shortened compared to the BDI nitrogen distances at 2.118(3) and 2.2098(19) Å for Mo1–N1 and Mo1–N2 respectively.

**Figure 5:**
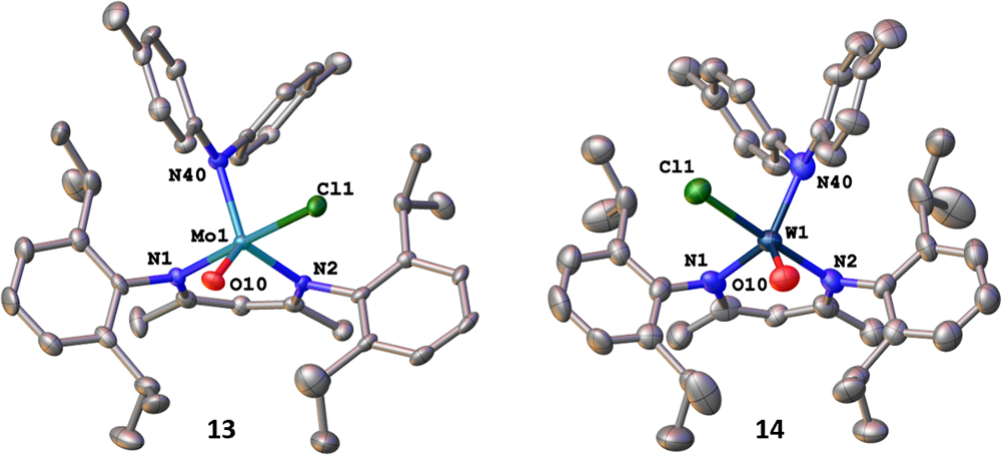
Molecular structures of the di-*p*-tolyl-amide complexes **13** and **14**. Hydrogen atoms and solvent lattice molecules have been omitted for clarity. Ellipsoids are shown at a probability level of 50%.

In all complexes described so far, the geometry around the metal center is square-pyramidal (except for **12**), with the oxo ligand O10 taking up the apical position of the coordination polyhedron. This situation changes if sterically more encumbering groups are introduced. Both **1** and **2** react smoothly with lithium di-*p*-tolyl-amide (LiN(Tol)_2_) giving access to dark green and purple colored complexes for molybdenum (**13**) and tungsten (**14**) (Figure 5). Both **13** and **14** show the expected d^1^ electronic structure by Evans method and EPR spectroscopy (see Experimental Section and Figure S13–S14 and S60, S61 for more information). It should be emphasized at this point, that the salt metathesis reactions leading to **13** and **14** give higher yields and cleaner conversions if the reaction is performed in toluene instead of diethyl ether. X-ray quality crystals grown from concentrated diethyl ether solutions and subsequent diffraction analysis, however, revealed that the metal centers are now coordinated in a trigonal-bipyramidal fashion displaying τ_5_ values of 0.89 (**13**) and 0.74 (**14**). Strikingly, the oxygen atom O10 is not in the apical position anymore, but has moved into the equatorial position of the trigonal bipyramid while the di-*p*-tolyl amide ligand occupies the expected position of O10. Overall, the di-*p*-tolyl amide, one BDI nitrogen and the oxo ligand occupy the equatorial position, while the other BDI nitrogen donor and the remaining chloride form the axial ligands. The change in conformation has no influence on the M1–O10 distances which are 1.662(2) and 1.669(9) Å in **13** and **14**, compared to 1.640(4) and 1.693(3) Å in **1** and **2**. We assume that the square pyramidal ligand arrangement is caused by the strong steric repulsion between the BDI and the *p*-tolylamide ligand. However, with M1–N40 distances of 1.995(4) and 1.984(14) Å these are slightly shorter compared to other molybdenum/tungsten–secondary amide interactions reported in the literature (2.00–2.12 Å).^112−122^ Further studies on the influence of this rearrangement on the reactivity of the oxo ligand (e.g. towards small phosphines)^44^ are currently ongoing.

We have also tried other nucleophiles (thiomesitolate, mesitylphosphanide) but these reactions resulted in the formation of complex (paramagnetic) mixtures, from which no defined material was isolated. Also, the synthesis of a dimethyl analogue employing methyl lithium or methyl magnesium halides have failed so far.

**Figure 6:**
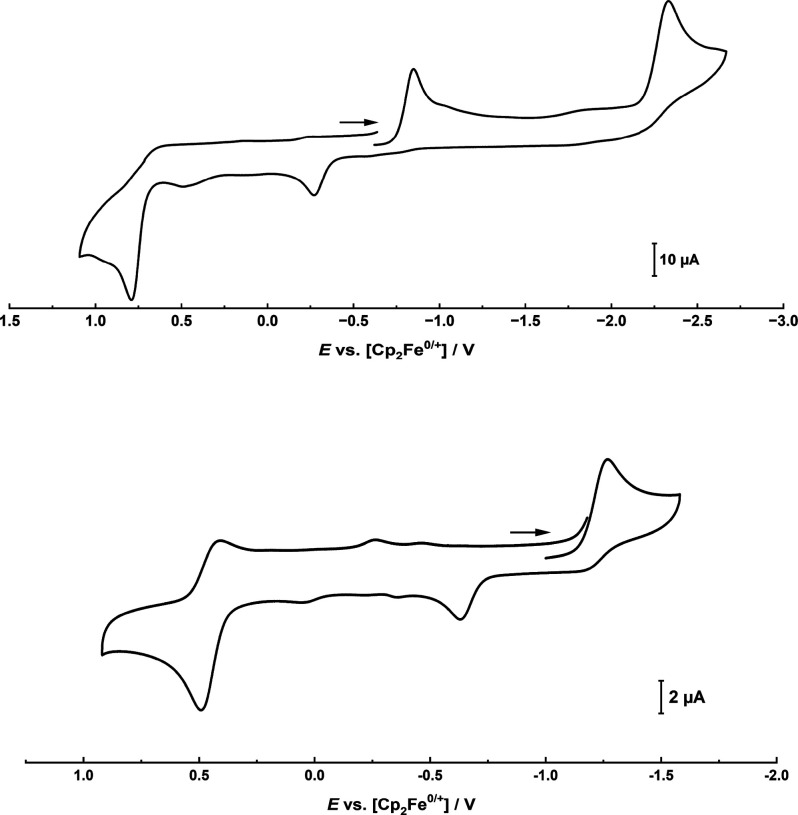
Cyclic voltammogram of complex **1** (top) and complex **2** (bottom), recorded in 0.2 M NBu_4_PF_6_ solution in MeCN at 298 K. Analyte concentration: 0.001 M. Scan Rate: 100 mV s^–1^.

Finally, we examined the electrochemical properties of complexes **1** and **2** with emphasis on the question: Can low-valent molybdenum(IV) and tungsten(IV) complexes be accessed? The isolation of such a species is of major interest for future applications of the BDI complexes in small molecule activation and catalysis. Cyclic voltammograms of **1** and **2** were recorded in MeCN (0.001 M analyte; 0.2 M NBu_4_PF_6_ electrolyte) and showed that both complexes can indeed be reduced but at very different potentials (Figure 6). The molybdenum complex **1** shows a reductive process at −0.95 V vs. Fc/[Fc]^+^ and a second one at −2.35 V vs. Fc/[Fc]^+^. Upon reoxidation, we found the second reduction to be irreversible. However, for the first reduction, reoxidation at −0.27 V vs Fc/[Fc]^+^ is observed. Although at 680 mV the peak-to-peak separation is relatively large, the Randles–Sevcik plot (Figure S49 and S50) is indicative of a reversible process. For complex **2** only one irreversible process is observed at −1.27 V vs. Fc/[Fc]^+^. Turning to the oxidative side, both complexes can be irreversibly oxidized at +0.79 V for **1** and +0.46 V vs. Fc/[Fc]^+^ for **2**.

**Scheme 4:**
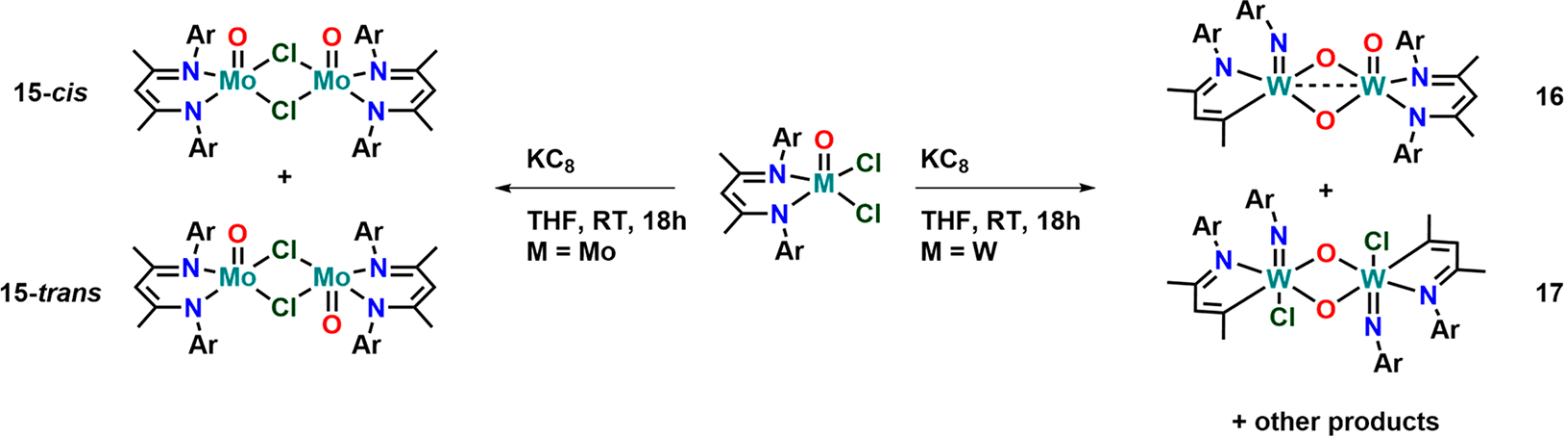
Synthesis and Attempt to Synthesize Low-Valent Molybdenum and Tungsten(IV) Complexes Supported by BDI Ligand Frameworks Using Potassium Graphite as a Reductant Ar = Dipp.

**Figure 7:**
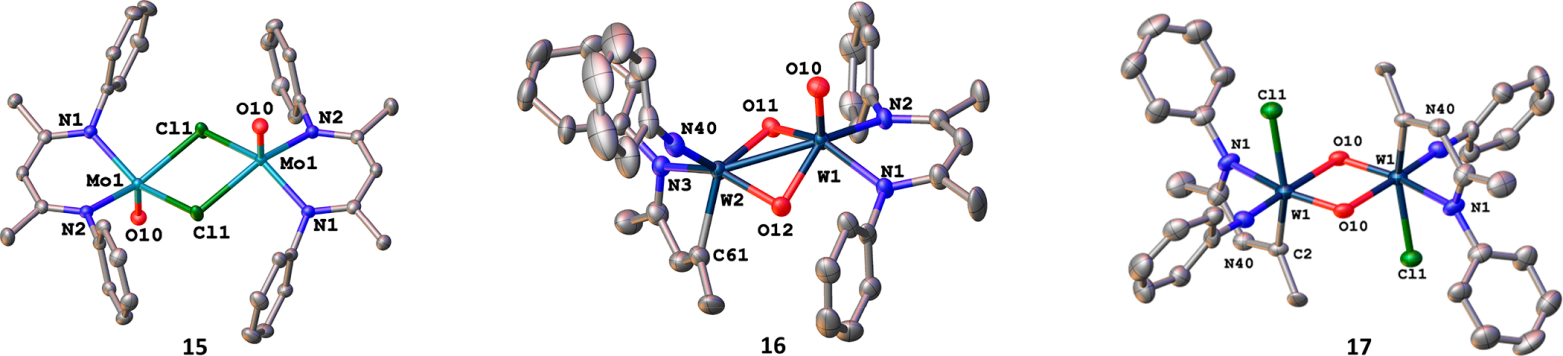
Molecular structures of the complexes **15**, **16**, and **17** (left to right) isolated after reduction of complexes **1** and **2** by KC_8_. Please note that for complex **15**, only the trans isomer is shown. Hydrogen atoms, additional lattice solvent molecules, and ^*i*^Pr groups of the Dipp substituents have been omitted for clarity. All ellipsoids are shown at a probability level of 50%.

Consequently, we attempted the chemical facilitation of these redox-processes. Even though the cyclic voltammograms indicate that high-valent molybdenum(VI) and tungsten(VI) complexes might be accessible we have not yet been able to isolate any useful products from the oxidation of **1** and **2** using various oxidizing reagents such as silver(I) salts, trityl chloride, tribromide salts, or PhICl_2_. This is in line with the results by Mösch-Zanetti and co-workers, showing that the combination of oxo-ligands in molybdenum(VI) ions and BDI ligands make a nonideal fit.^86^ Reductions of the complexes proved to be more successful. Mixing complex **1** with 1.5 equiv of potassium graphite (KC_8_) in THF and subsequent stirring overnight, results in a color change from dark green to light green/yellow and large orange/yellow blocks can be isolated after crystallization from pentane. ^1^H NMR studies revealed the presence of two diamagnetic species in solution in a 1:0.7 ratio (determined via the characteristic BDI backbone proton; Figure S15). The presence of a diamagnetic complex would be expected for a d^2^ configured molybdenum(IV) center and is in line with related d^2^-configured rhenium complexes.^44^ VT-NMR between 25 and 70 °C show that the ratio between the signals changes from 1:0.7 to 1:0.88, pointing to the presence of two isomers of the same species (Figure S16 and S22). The formulation of two isomers is also consistent with the fact, that ^1^H-DOSY measurements indicated the same diffusion coefficient for these two species (Figure S17). Given the structural information on two *C*_*2*_-symmetric species by ^1^H NMR spectroscopy we assume that the two possible isomers are the dimeric complexes **15-***cis* and **15-***trans*, whereby *cis* and *trans* refers to the orientation of the terminal metal oxo function (Scheme 4). The presence of two isomers is also supported by elemental analysis fitting perfectly for the molecular formula of complex **15**. X-ray diffraction analysis revealed the presence of a chloride bridged dimer showing the proposed *cis*–*trans* isomers with respect to the oxo orientation in a 95:5 ratio with the *cis* isomer being the minor one. Please note that even though the isomeric ratio in the crystal differs from the one determined in solution, this might also be related to crystal packing and picking effects. We have not checked all crystals obtained for their isomeric ratios. Complex **15** crystallizes in the monoclinic space group *P*2_1_/*n* with half a molecule in the asymmetric unit (Figure 7). The molybdenum–molybdenum distance was found to be 3.869(1) Å ruling out any direct interaction between the two molybdenum centers. The chloride bridge is almost symmetric displaying Mo–Cl distances of 2.4648(5) and 2.4841(5) Å. In line with the reduction of the metal center, i.e., an increase of its ionic radius, the molybdenum BDI-nitrogen distances increase from 2.090(3) Å in complex **1** to 2.1110(16) and 2.1184(16) in complex **15**. Similarly, the Mo1–O10 oxo distance slightly increases from 1.640(4) in **1** to 1.6585(14) Å in **15**. This is also in line with other molybdenum(IV) oxo complexes reported so far.^123−132^

**Scheme 5:**
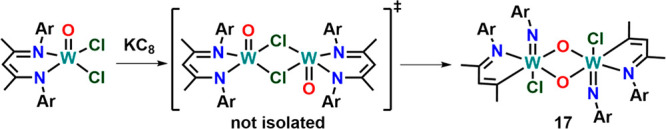
Proposed Reaction Sequence to the Formation of Complex **17** by One-Electron Reduction of Complex **2**

Turning to the tungsten complex **2** reduction under the same conditions applied for the reduction of complex **1** to **15** resulted in the formation of a complex product mixture (Figure S23), which could not be further separated. However, we were able to grow minor quantities of two different compounds (inseparable from each other) from the reaction mixture, giving some insight into its outcome (Figure 7). The first crystal (complex **16**) consisted of a dinuclear, oxo-bridged tungsten oxo and tungsten imido unit, on which the BDI ligand remained intact on the tungsten oxo-fragment, but has decomposed to a MAD ligand (MAD = 4-((2,6-diisopropylphenyl)imino)pent-2-enide) via a nitrene transfer reaction on the tungsten imido side. We believe that at least one of the additional oxo ligand, bridging the two tungsten centers comes from residual water in the crystallization solvent. Overall, counting the anionic charges present in complex **16**, the two tungsten centers have the formal +V oxidation state. This is in line with the W–W distance of 2.663(3) Å, indicating a weak bond between the two tungsten centers and a d^1^–d^1^ interaction. The bond lengths within the tungsten oxo bridges range from 1.916(4)–1.976(4) Å being substantially longer than the terminal oxo distance, 1.704(4) Å in **2**. These values are in accordance with the literature for other diamond-core μ-O,O bridged tungsten(V) complexes.^133−140^ The tungsten imido distance was found to be 1.750(5) Å, which is also in line with previously reported tungsten Dipp-imido complexes.^141−150^ While the exact mechanism of the formation of complex **16** is unclear, the formation of the other crystallized reaction product can be rationalized more easily. Complex **17** is a dimeric diamond-core μ-O,O complex. However, contrasting complex **16**, both BDI ligands have been degraded to the MAD ligand framework by nitrene transfer reaction, resulting in the formation of two oxo-imido tungsten units, which are bridged via the oxygen atoms. In addition, one chloride ion remains on each tungsten atom. Overall, both tungsten atoms are in the +VI oxidation state and we propose the following reaction: Initial reduction of W^V^ to a transient W^IV^ complex takes place. This W^IV^ complex is not stable and undergoes a two-electron oxidative nitrene transfer reaction, thus degrading the BDI ligand and forming the MAD ligand (Scheme 5).

Nitrene transfer reactions are commonly observed with BDI ligands, especially with low-valent early transition metals,^3^ e.g., titanium,^151−156^ niobium,^35,36,109^ and rhenium.^44^ Complex **17** crystallizes in the triclinic space group *P*1̅ with half a molecule in the asymmetric unit. A W1–W1 distances of 3.1742(14) Å clearly rules out any direct bonding between the two tungsten centers. Each tungsten center is hexacoordinated in a distorted octahedral coordination environment by the MAD ligand, the two bridging oxides, the new imido ligand and the chloride ligand. The tungsten imido distance of 1.764(12) Å for W1–N40 is substantially longer as in the terminal oxo complexes reported here (between 1.65–1.69 Å) but matches well with previously reported Dipp-imido tungsten complexes (1.73–1.77 Å in average).^141,143,148,149,157,158^ The oxo bridge is asymmetric with W1–O10 distances of 1.801(10) and 2.220(11) Å. These distances are slightly longer compared to the tungsten(V) dimer **16**, which is expected considering the lack of a d^1^–d^1^ W^V^–W^V^ interaction.

## Conclusion

We have presented a series of 17 new molybdenum and tungsten complexes supported by the BDI ligand framework (or its degradation products). Both the molybdenum(V) and tungsten(V) complexes **1** and **2** are excellent precursors for the coordination of neutral and anionic donor ligands such as DMAP, OPEt_3_, mesitolates, and primary or secondary amides. Interestingly, the complexes do not react (neither via adduct formation, nor OAT) with small phosphines (PEt_3_), contrasting the otherwise well-examined OAT reactivity of high-valent molybdenum(VI) and tungsten(VI) complexes.^129−132^ Cyclic voltammetry revealed both complexes to be reducible with one electron processes. Chemical reduction of complex **1** forged the first molybdenum(IV) complex **15** supported by the BDI ligand framework. In contrast, reduction of the tungsten complex **2** gave access to intractable mixtures, from which single crystals of the tungsten(VI) complex **17** supported by a MAD ligand were isolated. Complex **17** forms most likely by a nitrene transfer degradation of the BDI ligand,^3^ via an intermediate tungsten(IV) complex. In summary, this study not only shows the subtle difference in the stability of the +IV oxidation states of molybdenum and tungsten, but also gives access to an interesting new starting material, complex **15**, whose reactivity and propensity to engage in small molecule activation will be further explored by our group.

## Experimental Section

### General Remarks

If not stated otherwise, all transformations were conducted in an argon filled glovebox under inert conditions. Solvents were dried by an MBraun SPS system and stored over activated molecular sieves (3 Å) for at least 1 day prior to use. C_6_D_6_ was dried over sodium/benzophenone followed by vacuum transfer and three freeze–pump–thaw cycles. H[BDI], Li[BDI],^159^ KC_8_,^160,161^ MoOCl_3_(THF)_2_,^162^ and WOCl_3_(THF)_2_^163^ were synthesized according to the literature; LiOMes, LiNHTol, and LiNTol_2_ were obtained by deprotonating the corresponding phenol or aniline in pentane using 1.2 equiv of *n*-BuLi and filtering off the white products. DMAP and OPEt_3_ were used as commercially received, without any additional purification or drying steps. NMR spectra were collected at ambient temperature on an Ascent 400 or Ascent 700 spectrometer; ^1^H and ^13^C NMR chemical shifts (δ) are reported in ppm and were calibrated to residual solvent peaks. IR spectra were collected using a Bruker Alpha IR spectrometer using an ATR detector setup. UV–vis spectra were collected on a PerkinElmer Lambda XLS+ spectrometer in 10 mm fused silica cuvettes. Cyclic voltammetry was recorded using a BioLogic potentiostat and a three-electrode array (working electrode: glassy carbon, counter electrode: platinum, reference electrode: silver). Solvents for cyclic voltammetry (THF and DCM, electrochemical grade) have been dried over activated molecular sieves for at least 48 h prior to use. The supporting electrolyte NBu_4_PF_6_ (electrochemical grade) was recrystallized three times from absolute ethanol and dried under a high vacuum (1 × 10^–3^ mbar) at 80 °C for 48 h and was stored in an argon-filled glovebox. All experiments have been performed in 0.2 M solutions of NBu_4_PF_6_ in the corresponding solvent at 0.001 M solutions of the analyte under strictly inert conditions in an argon-filled glovebox. Purity of the electrolyte solutions was confirmed by back-ground scans prior to addition of the analyte, showing no redox-events. Electron paramagnetic spectroscopy was conducted on a Magnettech 5000 X-Band spectrometer equipped with a variable temperature unit in 3 mm o.d. J-Young style fused silica tubes. Simulations were performed using the garlic and pepper functions of the EasySpin package for MatLab and parameters obtained with least-squares fitting.^164^

### Synthetic Procedures

#### General Procedure for the Synthesis of **1**–**4**

Metal precursors MOCl_3_(THF)_2_ and ligand LiBDI^R^(Et_2_O) were dissolved separately 5 mL THF each and cooled to −40 °C. The ligand was added dropwise under stirring while the mixture was allowed to warm to room temperature. After 24 (Mo) or 48 (W) hours the solvent was removed *in vacuo* and the residue dissolved in diethyl ether. After filtration the solution was concentrated and crystals of compounds **1**–**4** were obtained within 24 h.

#### BDI^Dipp^MoOCl_2_ (**1**)

From MoOCl_3_(THF)_2_ (1 equiv, 0.500 mmol, 181 mg) and LiBDI^Dipp^(Et_2_O) (1 equiv, 0.525 mmol, 257 mg). 238 mg (79%) of a dark green solid. ^1^H NMR (C_6_D_6_, 298 K) δ (ppm) 8.60, 6.57, 2.11, 1.83, −1.61. Elemental analysis (%) calc’d for C_29_H_41_N_2_O_1_Cl_2_Mo_1_: C, 58.00; H, 6.88; N, 4.67. Found C, 57.64; H, 6.77; N, 4.78. μ_eff_ (Evans Method, C_6_D_6_): 1.75 μB. IR (cm^–1^) 2961, 2928, 2869, 1619, 1541, 1464, 1441, 1384, 1354, 1313, 1284, 1245, 1227, 1178, 1098, 1058, 1041, 1023, 988, 935, 861, 849, 798, 788, 763, 704, 533, 463. UV–vis λ_max_ 320 nm (ε = 7000 L mol^–1^ cm^–1^), 393 nm (ε = 5800 L mol^–1^ cm^–1^), 583 nm (ε = 950 L mol^–1^ cm^–1^).

#### BDI^Dipp^WOCl_2_ (**2**)

From WOCl_3_(THF)_2_ (1 equiv, 0.385 mmol, 173 mg) and LiBDI^Dipp^(Et_2_O) (1.05 equiv, 0.404 mmol, 190 mg). 199 mg (75%) of a gray solid. ^1^H NMR (C_6_D_6_, 298 K) δ (ppm) 8.14, 7.53, 1.83, 1.41, 0.99, 0.40. Elemental analysis (%) calc’d for C_29_H_41_N_2_O_1_Cl_2_W_1_: C, 50.60; H, 6.00; N, 4.07. Found C, 50.80; H, 5.72; N, 3.81. μ_eff_ (Evans Method, C_6_D_6_): 1.30 μB. IR (cm^–1^) 2965, 2930, 2871, 1633, 1590, 1544, 1515, 1460, 1441, 1384, 1364, 1315, 1290, 1245, 1182, 1147, 1098, 1058, 1023, 994, 960, 935, 866, 849, 798, 759, 706, 535, 453, 422. UV–vis λ_max_ 336 nm (ε = 10400 L mol^–1^ cm^–1^), 369 nm (ε = 13300 L mol^–1^ cm^–1^).

#### BDI^Mes^MoOCl_2_ (**3**)

From MoOCl_3_(THF)_2_ (1 equiv, 0.500 mmol, 181 mg) and LiBDI^Mes^(Et_2_O) (1.05 equiv, 0.525 mmol, 218 mg). To ensure all lithium chloride to precipitate, the ether extract is stirred for 6 h at room temperature prior to filtration. 162 mg (62%) of a brown to green solid. ^1^H NMR (C_6_D_6_, 298 K) δ (ppm) 5.81, 3.75, 1.71, 1.54, 1.49, 1.41, 1.37, −3.16. Elemental analysis (%) calc’d for C_23_H_29_N_2_O_1_Cl_2_Mo_1_: C, 53.50; H, 5.68; N, 5.43. Found C, 50.44; H, 5.60; N, 4.93. The low carbon value can be explained by metal carbide formation which is a common problem in group VI chemistry. μ_eff_ (Evans Method, C_6_D_6_): 1.61 μB. IR (cm^–1^) 2969, 2920, 2856, 1609, 1562, 1535, 1509, 1476, 1423, 1370, 1341, 1282, 1233, 1209, 1192, 1141, 1023, 986, 964, 937, 896, 859, 817, 735, 702, 657, 629, 569, 506, 416. UV–vis λ_max_ 323 (ε = 7900 L mol^–1^ cm^–1^), 376 (ε = 5600 L mol^–1^ cm^–1^), 560 nm (ε = 1000 L mol^–1^ cm^–1^).

#### BDI^Mes^WOCl_2_ (**4**)

From WOCl_3_(THF)_2_ (1 equiv, 0.300 mmol, 135 mg) and LiBDI^Mes^(Et_2_O) (1.05 equiv, 0.315 mmol, 131 mg). To ensure all lithium chloride to precipitate, the ether extract is stirred for 6 h at room temperature prior to filtration. 89 mg (50%) of a brown solid. ^1^H NMR (C_6_D_6_, 298 K) δ (ppm) 6.44, 3.86, 3.32, 1.70, 1.43. Elemental analysis (%) calc’d for C_23_H_29_N_2_O_1_Cl_2_W_1_: C, 45.72; H, 4.84; N, 4.64. Found C, 45.93; H, 5.06; N, 4.20. μ_eff_ (Evans Method, C_6_D_6_): 1.37 μB. IR (cm^–1^) 2918, 2856, 1609, 1517, 1476, 1431, 1362, 1343, 1313, 1288, 1243, 1229, 1211, 1194, 1143, 1113, 1025, 994, 964, 853, 829, 817, 731, 657, 639, 629, 596, 572, 543, 500, 425. UV–vis λ_max_ 257 nm (ε = 7700 L mol^–1^ cm^–1^), 324 nm (ε = 9800 L mol^–1^ cm^–1^).

#### General Procedure for the Synthesis of **5**–**8**

The corresponding base complex was dissolved in 5 mL of diethyl ether, and a solution of the reactant (OPEt_3_, DMAP) in 2 mL diethyl ether was added in one portion. After stirring for 2 h, the reaction mixture was filtrated and evaporated to dryness. The crude samples were then recrystallized from concentrated pentane solutions (0.5 mL) at room temperature.

#### BDI^Dipp^MoOCl_2_(DMAP) (**5**)

From BDI^Dipp^MoOCl_2_ (1 equiv, 0.167 mmol, 100 mg) and DMAP (1 equiv, 0.167 mmol, 20 mg). 63 mg (52%) of a bright green solid. ^1^H NMR (C_6_D_6_, 298 K) δ (ppm) 8.43, 6.10, 2.09. Elemental analysis (%) calc’d for C_36_H_51_N_4_O_1_Cl_2_Mo_1_: C, 59.83; H, 7.11; N, 7.75. Found C, 59.22; H, 7.53; N, 7.00. μ_eff_ (Evans Method, C_6_D_6_): 1.51 μB. IR (cm^–1^) 2965, 2926, 2867, 1623, 1544, 1517, 1462, 1439, 1366, 1317, 1247, 1233, 1166, 1100, 1062, 1013, 978, 937, 855, 798, 761, 735, 696, 529, 457. UV–vis λ_max_ 257 nm (ε = 11700 L mol^–1^ cm^–1^), 294 nm (ε = 13000 L mol^–1^ cm^–1^), 583 nm (ε = 800 L mol^–1^ cm^–1^).

#### BDI^Dipp^WOCl_2_(DMAP) (**6**)

From BDI^Dipp^WOCl_2_ (1 equiv, 0.102 mmol, 70 mg) and DMAP (1 equiv, 0.102 mmol, 12 mg). 42 mg (51%) of a dark green - brown solid. ^1^H NMR (C_6_D_6_, 298 K) δ (ppm) 8.45, 7.69, 6.88, 6.09, 5.42, 2.81, 2.22, 1.97, 1.67, 1.34, 0.60. Elemental analysis (%) calc’d for C_36_H_51_N_4_O_1_Cl_2_W_1_ · 1.5 Et_2_O:: C, 54.73; H, 7.22; N, 6.08. Found C, 54.98; H, 7.47; N, 5.81. μ_eff_ (Evans Method, C_6_D_6_): 1.54 μB. IR (cm^–1^) 2969, 2928, 2869, 1646, 1623, 1544, 1521, 1462, 1439, 1382, 1366, 1317, 1286, 1233, 1168, 1107, 1060, 1015, 949, 857, 800, 761, 729, 704, 631, 610, 553, 529, 459, 414, 404. UV–vis λ_max_ 258 nm (ε = 19900 L mol^–1^ cm^–1^), 270 nm (ε = 19800 L mol^–1^ cm^–1^), 342 nm (ε = 14200 L mol^–1^ cm^–1^), 559 nm (ε = 1700 L mol^–1^ cm^–1^).

#### BDI^Dipp^MoOCl_2_(OPEt_3_) (**7**)

From BDI^Dipp^MoOCl_2_ (1 equiv, 0.167 mmol, 100 mg) and OPEt_3_ (1 equiv, 0.167 mmol, 22 mg). 73 mg (60%) of a dark green solid. ^1^H NMR (C_6_D_6_, 298 K) δ (ppm) 8.57, 6.56, 3.8, 2.40, 1.33. Elemental analysis (%) calc’d for C_35_H_56_N_2_O_2_P_1_Cl_2_Mo_1_: C, 57.22; H, 7.68; N, 3.81. Found C, 57.66; H, 7.29; N, 4.10. μ_eff_ (Evans Method, C_6_D_6_): 1.82 μB. IR (cm^–1^) 2963, 2928, 2871, 1535, 1462, 1439, 1384, 1354, 1315, 1282, 1247, 1098, 1058, 1023, 984, 937, 859, 796, 761, 704, 527, 455. UV–vis λ_max_ 313 nm (ε = 8900 L mol^–1^ cm^–1^), 391 nm (ε = 6000 L mol^–1^ cm^–1^), 599 nm (ε = 960 L mol^–1^ cm^–1^).

#### BDI^Dipp^WOCl_2_(OPEt_3_) (**8**)

From BDI^Dipp^WOCl_2_ (1 equiv, 0.145 mmol, 0.100 mg) and OPEt_3_ (1 equiv, 0.145 mmol, 20 mg). 40 mg (33%) of an orange solid. ^1^H NMR (C_6_D_6_, 298 K) δ (ppm) 7.95, 7.36, 4.00, 2.48, 1.67, 1.60, 1.51, 1.46 0.39. Elemental analysis (%) calc’d for C_35_H_56_N_2_O_2_P_1_Cl_2_W_1_: C, 57.22; H, 7.68; N, 3.81. Found C, 56.89; H, 7.66; N, 3.43. μ_eff_ (Evans Method, C_6_D_6_): 1.76 μB. IR (cm^–1^) 2967, 2940, 2867, 1533, 1462, 1437, 1407, 1382, 1364, 1315, 1284, 1254, 1166, 1102, 1066, 1051, 1035, 1017, 986, 957, 857, 798, 786, 766, 729, 706, 680, 635, 608, 525, 478, 453, 431. UV–vis λ_max_ 258 nm (ε = 12700 L mol^–1^ cm^–1^), 322 nm (ε = 5600 L mol^–1^ cm^–1^), 373 nm (ε = 6500 L mol^–1^ cm^–1^), 407 nm (ε = 4900 L mol^–1^cm^–1^).

#### General Procedure for the Synthesis of **9**–**14**

The corresponding base complex was dissolved in 5 mL of diethyl ether and cooled to −40 °C. A solution of the reactant in 5 mL diethyl ether was added dropwise with continuous cooling. After the addition the reaction mixture was allowed to warm slowly to room temperature and stirred for 16 h. The solvent was removed *in vacuo* and the solids suspended in pentane. After filtration the solution was evaporated to dryness to give the title compound.

#### BDI^Dipp^MoOCl(OMes) (**9**)

From BDI^Dipp^MoOCl_2_ (1 equiv, 0.117 mmol, 70 mg) and LiOMes (2.1 equiv, 0.246 mmol, 35 mg). 53 mg (65%) of a dark blue to purple solid. ^1^H NMR (C_6_D_6_, 298 K) δ (ppm) 7.84, 6.60, 2.16, 2.09, 1.53. Elemental analysis (%) calc’d for C_38_H_52_N_2_O_2_Cl_1_Mo_1_ · 0.55 C_5_H_12_: C, 66.15; H, 7.98; N, 3.79. Found C, 66.02; H, 7.62; N, 3.44. μ_eff_ (Evans Method, C_6_D_6_): 1.57 μB. IR (cm^–1^) 2963, 2930, 2869, 1537, 1511, 1476, 1462, 1439, 1358, 1313, 1276, 1247, 1219, 1180, 1151, 1100, 1055, 1021, 974, 957, 935, 855, 827, 798, 784, 761, 735, 706, 594, 547, 531, 453, 431. UV–vis λ_max_ 308 nm (ε = 14700 L mol^–1^ cm^–1^), 366 nm (ε = 13300 L mol^–1^ cm^–1^), 549 nm (ε = 3600 L mol^–1^ cm^–1^).

#### BDI^Dipp^WOCl(OMes) (**10**)

From BDI^Dipp^WOCl_2_ (1 equiv, 0.102 mmol, 70 mg) and LiOMes (2.1 equiv, 0.214 mmol, 31 mg). 65 mg (81%) of a dark red solid. ^1^H NMR (C_6_D_6_, 298 K) δ (ppm) 7.85, 7.49, 2.76, 1.75, 1.59. Elemental analysis (%) calc’d for C_38_H_52_N_2_O_2_Cl_1_W_1_ · 0.5 C_5_H_12_: C, 59.02; H, 7.09; N, 3.40. Found C, 59.01; H, 6.83; N, 2.91. μ_eff_ (Evans Method, C_6_D_6_): 1.26 μB. IR (cm^–1^) 2963, 2926, 2869, 1619, 1593, 1541, 1476, 1464, 1439, 1374, 1356, 1311, 1258, 1241, 1213, 1151, 1100, 1055, 1019, 978, 957, 935, 851, 835, 796, 759, 725, 706, 680, 651, 576, 547, 533, 500, 459. UV–vis λ_max_ 258 nm (ε = 12600 L mol^–1^ cm^–1^), 343 nm (ε = 11600 L mol^–1^ cm^–1^).

#### BDI^Mes^MoO(OMes)_2_ (**11**)

From BDI^Mes^MoOCl_2_ (1 equiv, 0.116 mmol, 60 mg) and LiOMes (2.2 equiv, 0.256 mmol, 36 mg). 60 mg (72%) of a dark blue to purple solid. ^1^H NMR (C_6_D_6_, 298 K) δ (ppm) 7.85, 7.49, 3.49, 2.80, 1.77, 1.58, 1.36, −0.11. Elemental analysis (%) calc’d for C_41_H_51_N_2_O_3_Mo_1_: C, 68.80; H, 7.18; N, 3.91. Found C, 69.12; H, 7.42; N, 3.64. μ_eff_ (Evans Method, C_6_D_6_): 1.63 μB. IR (cm^–1^) 2963, 2926, 2869, 1623, 1588, 1537, 1511, 1476, 1464, 1437, 1360, 1309, 1276, 1247, 1219, 1151, 1100, 1055, 1021, 974, 957, 935, 894, 884, 853, 827, 798, 784, 761, 735, 725, 706, 649, 627, 594, 580, 541, 531, 453, 429. UV–vis λ_max_ 265 nm (ε = 12400 L mol^–1^ cm^–1^), 365 nm (ε = 9000 L mol^–1^ cm^–1^), 549 nm (ε = 2300 L mol^–1^ cm^–1^).

#### BDI^Dipp^MoO(NHTol)_2_ (**12**)

From BDI^Dipp^MoOCl_2_ (1 equiv, 0.117 mmol, 70 mg) and LiNHTol (2.1 equiv, 0245 mmol, 28 mg). To ensure all lithium chloride to precipitate, the pentane extract is stirred for 6 h at room temperature prior to filtration. 76 mg (88%) of an intense dark green solid. ^1^H NMR (C_6_D_6_, 298 K) δ (ppm) 7.40, 6.44, 2.11. Elemental analysis (%) calc’d for C_43_H_57_N_4_O_1_Mo_1_: C, 67.05; H, 7.74; N, 7.55. Found C, 67.05; H, 7.63; N, 7.29. The low carbon value can be explained by metal carbide formation which is a common problem in group VI chemistry. μ_eff_ (Evans Method, C_6_D_6_): 1.52 μB. IR (cm^–1^) 3326, 3304, 2961, 2926, 2867, 1621, 1588, 1548, 1517, 1503, 1462, 1435, 1376, 1315, 1256, 1174, 1098, 1058, 1021, 970, 939, 868, 855, 808, 794, 761, 702, 637, 596, 525, 506, 490, 455, 414, 404. UV–vis λ_max_ 275 nm (ε = 18000 L mol^–1^ cm^–1^), 295 nm (ε = 19300 L mol^–1^ cm^–1^), 369 nm (ε = 11400 L mol^–1^ cm^–1^), 549 nm (ε = 2100 L mol^–1^ cm^–1^).

#### BDI^Dipp^MoOCl(N(Tol)_2_) (**13**)

Equivalent to the general procedure in toluene. From BDI^Dipp^MoOCl_2_ (1 equiv, 0.262 mmol, 157 mg) and LiN(Tol)_2_ (1.1 equiv, 0.289 mmol, 59 mg). To ensure all lithium chloride to precipitate, the pentane extract is stirred for 6 h at room temperature prior to filtration. 105 mg (52%) of a dark green solid. ^1^H NMR (C_6_D_6_, 298 K) δ (ppm) 6.57, 1.35. Elemental analysis (%) calc’d for C_43_H_55_Cl_1_N_3_O_1_Mo_1_: C, 67.84; H, 7.28; N, 7.29. Found C, 64.29; H, 7.31; N, 4.97. The low carbon value can be explained by metal carbide formation which is a common problem in group VI chemistry. μ_eff_ (Evans Method, C_6_D_6_): 1.59 μB. IR (cm^–1^) 2961, 2926, 2869, 1609, 1507, 1460, 1437, 1366, 1315, 1298, 1252, 1209, 1178, 1102, 1055, 1021, 984, 955, 937, 892, 855, 806, 796, 759, 704, 582, 549, 529, 500, 455. UV–vis λ_max_ 258 nm (ε = 19500 L mol^–1^ cm^–1^), 286 nm (ε = 18700 L mol^–1^ cm^–1^), 384 nm (ε = 8100 L mol^–1^ cm^–1^).

#### BDI^Dipp^WOCl(N(Tol)_2_) (**14**)

From BDI^Dipp^WOCl_2_ (1 equiv, 0.236 mmol, 162 mg) and LiN(Tol)_2_ (1.1 equiv, 0.259 mmol, 53 mg). 104 mg (52%) of a dark brown solid. ^1^H NMR (C_6_D_6_, 298 K) δ (ppm) 8.59, −1.96 Elemental analysis (%) calc’d for C_43_H_55_Cl_1_N_3_O_1_W_1_: C, 60.82; H, 6.53; N, 4.95. Found C, 60.48; H, 6.77; N, 4.75. μ_eff_ (Evans Method, C_6_D_6_): 1.45 μB. IR (cm^–1^) 3024, 2963, 2922, 2867, 1609, 1590, 1515, 1460, 1439, 1382, 1362, 1315, 1243, 1178, 1107, 1055, 1039, 1019, 992, 966, 937, 906, 866, 806, 759, 704, 680, 639, 592, 502. UV–vis λ_max_ 258 nm (ε = 12100 L mol^–1^ cm^–1^), 295 nm (ε = 13600 L mol^–1^ cm^–1^), 639 nm (ε = 1700 L mol^–1^ cm^–1^).

#### [BDI^Dipp^MoOCl]_2_ (**15**)

Solid BDI^Dipp^MoOCl_2_ (1 equiv, 0.266 mol, 160 mg) and KC_8_ (1.5 equiv, 0.400 mmol, 54 mg) were mixed and cooled to −40 °C. Seven milliliters of cold THF was added in one portion, and the mixture was slowly warmed to room temperature over 16 h. After filtration, the solvent was removed and the residue suspended in 10 mL pentane. The mixture was filtrated again and concentrated to 5 mL. After 1 h a light green solid started to precipitate. The solid was filtered after 24 h and washed with a minimal amount of pentane to give clean [BDI^Dipp^MoOCl]_2_. 65 mg (43%) of a light green solid. ^1^H NMR (C_6_D_6_, 298 K, 700 MHz): Isomer A δ 7.00 (t, *J* = 7.7 Hz, 2H, Aryl para-*H*), 6.92 (d, *J* = 7.7 Hz, 4H, Aryl meta-*H*), 4.88 (s, 1H, BDI α-*H*), 3.39 (hept, *J* = 6.8 Hz, 2H, ^*i*^Pr–C*H*), 2.63 (hept, *J* = 6.8 Hz, 2H, ^*i*^Pr–C*H*), 1.49 (s, 6H, BDI-C*H*_*3*_), 1.45 (d, *J* = 6.8 Hz, 6H, ^*i*^Pr–C*H*_*3*_), 1.24 (d, *J* = 6.8 Hz, 6H, ^*i*^Pr–C*H*_*3*_), 1.23 (d, *J* = 6.8 Hz, 6H, ^*i*^Pr–C*H*_*3*_), 1.04 (d, *J* = 6.8 Hz, 6H, ^*i*^Pr–C*H*_*3*_), Isomer B δ 7.03 (t, *J* = 7.7 Hz, 2H, Aryl para-*H*), 6.94 (d, *J* = 7.7 Hz, 4H, Aryl meta-*H*), 4.81 (s, 1H, BDI α-*H*), 3.29 (hept, *J* = 6.8 Hz, 2H, ^*i*^Pr–C*H*), 2.72 (hept, *J* = 6.8 Hz, 2H, ^*i*^Pr–C*H*), 1.43 (s, 6H, BDI-C*H*_*3*_), 1.38 (d, *J* = 6.8 Hz, 6H, ^*i*^Pr–C*H*_*3*_), 1.20 (d, *J* = 6.8 Hz, 6H, ^*i*^Pr–C*H*_*3*_), 1.06 (d, *J* = 6.8 Hz, 12H, ^*i*^Pr–C*H*_*3*_). ^13^C{^1^H} NMR (C_6_D_6_, 298 K, 176 MHz) Isomer A δ 167.30 (BDI β-*C*), 149.07 (Aryl *C*), 141.23 (Aryl-*C*-^*i*^Pr), 141.12 (Aryl-*C*-^*i*^Pr), 126.20 (Aryl-*C*H), 124.46 (Aryl-*C*H), 123.16 (Aryl-*C*), 104.04 (BDI-*C*H), 29.03 (^*i*^Pr-*C*H), 26.96 (^*i*^Pr-*C*H), 25.49 (*C*H_3_), 24.65 (*C*H_3_), 24.58 (*C*H_3_), 24.41 (*C*H_3_), 24.33 (*C*H_3_). Isomer B δ 167.12 (BDI β-*C*), 149.26 (Aryl *C*), 141.61 (Aryl-*C*-^*i*^Pr), 140.54 (Aryl-*C*-^*i*^Pr), 125.88 (Aryl-*C*H), 125.11 (Aryl-*C*H), 122.68 (Aryl-*C*H), 103.85 (BDI-*C*H), 29.23 (^*i*^Pr-*C*H), 26.84 (^*i*^Pr-*C*H), 25.11 (*C*H_3_), 24.92 (*C*H_3_), 24.74 (*C*H_3_), 24.63 (*C*H_3_), 24.48 (*C*H_3_). Elemental analysis (%) calc’d for C_58_H_82_N_4_O_2_Cl_2_Mo_2_: C, 61.64; H, 7.31; N, 4.96. Found C, 61.44; H, 7.04; N, 4.75. IR (cm^–1^) 2963, 2928, 2867, 1529, 1460, 1437, 1366, 1317, 1284, 1252, 1176, 1100, 1058, 1019, 980, 937, 853, 796, 770, 757, 704, 635, 543, 527, 451. UV–vis λ_max_ 228 nm (ε = 7200 L mol^–1^ cm^–1^), 258 nm (ε = 10200 L mol^–1^ cm^–1^), 381 nm (ε = 9000 L mol^–1^ cm^–1^).

### Reduction of **2**

Solid BDI^Dipp^WOCl_2_ (1 equiv, 0.232 mmol, 160 mg) and KC_8_ (1.5 equiv, 0.349 mmol, 47 mg) were mixed and cooled to −40 °C. Seven milliliters of cold THF was added in one portion, and the mixture was slowly warmed to room temperature over 16 h. After filtration, the solvent was removed and the residue suspended in 10 mL pentane. The mixture was filtrated again and the solvent was evaporated. Few single crystals of **16** and **17** were obtained by stopping the evaporation of the pentane extract at 1 mL volume of pentane and leaving the solution overnight at room temperature. The crude NMR can be seen in Figure S23.

### X-ray Crystallography

X-ray diffraction experiments were performed at the analytical facility of the University of Innsbruck. Data collection was performed using the ApexIV software package on a Bruker D8 Quest instrument. Data refinement and reduction were performed using the Bruker ApexIV suite 2022. Using the OLEX2 software package,^165^ all structures were solved with SHELXT^166^ and refined with SHELXL.^167^ Strongly disordered solvent molecules have been removed using the SQUEEZE operation.^168^ All non-hydrogen atoms were refined anisotropically, and hydrogen atoms were included at the geometrically calculated positions and refined using a riding model. For further crystallographic details, see Tables S1 and S2 in the Supporting Information.

## Supporting Information Available

NMR and EPR spectra, IR spectra, crystallographic details, cyclic voltammograms, and UV–vis spectra (PDF)
