# Supplementary material for: Expanding the Utility of β-Diketiminate Ligands in Heavy Group VI Chemistry of Molybdenum and Tungsten
Source: Organometallics. 2023 Jun 1;42(12):1411–24. doi: 10.1021/acs.organomet.3c00056 (PMC10302891; doi:10.1021/acs.organomet.3c00056)
Supplement: Supplementary file 1 — om3c00056_si_001.pdf [file om3c00056_si_001.pdf]

Expanding the Utility of β-Diketiminate Ligands in Heavy Group VI Chemistry of Molybdenum and Tungsten

Daniel Leitner[a] Benjamin Wittwer,[a] Florian Neururer,[a] Michael Seidl,[a] Klaus Wurst,[a] Frank Tambornino[b] and Stephan Hohloch[a]*

Contents

1. NMR spectra 2
2. UV-Vis spectra 26
3. IR spectra 30
4. Crystallographic details 38
5. Cyclic Voltammetry 41
6. EPR spectra 43

[a] *University of Innsbruck, Faculty of Chemistry and Pharmacy, Institute for General, Inorganic and Theoretical Chemistry, Innrain 80 – 82 Innsbruck, 6020 Austria.*
[b] *Phillips-University Marburg, Fachbereich Chemie and Wissenschaftlichen Zentrum für Materialwissenschaften (WZMW), Hans-Meerwein-Straße 4, 35043 Marburg, Germany*

Electronic Supplementary Information (ESI) available: [details of any supplementary information available should be included here]. See DOI: 10.1039/x0xx00000x

1. NMR spectra

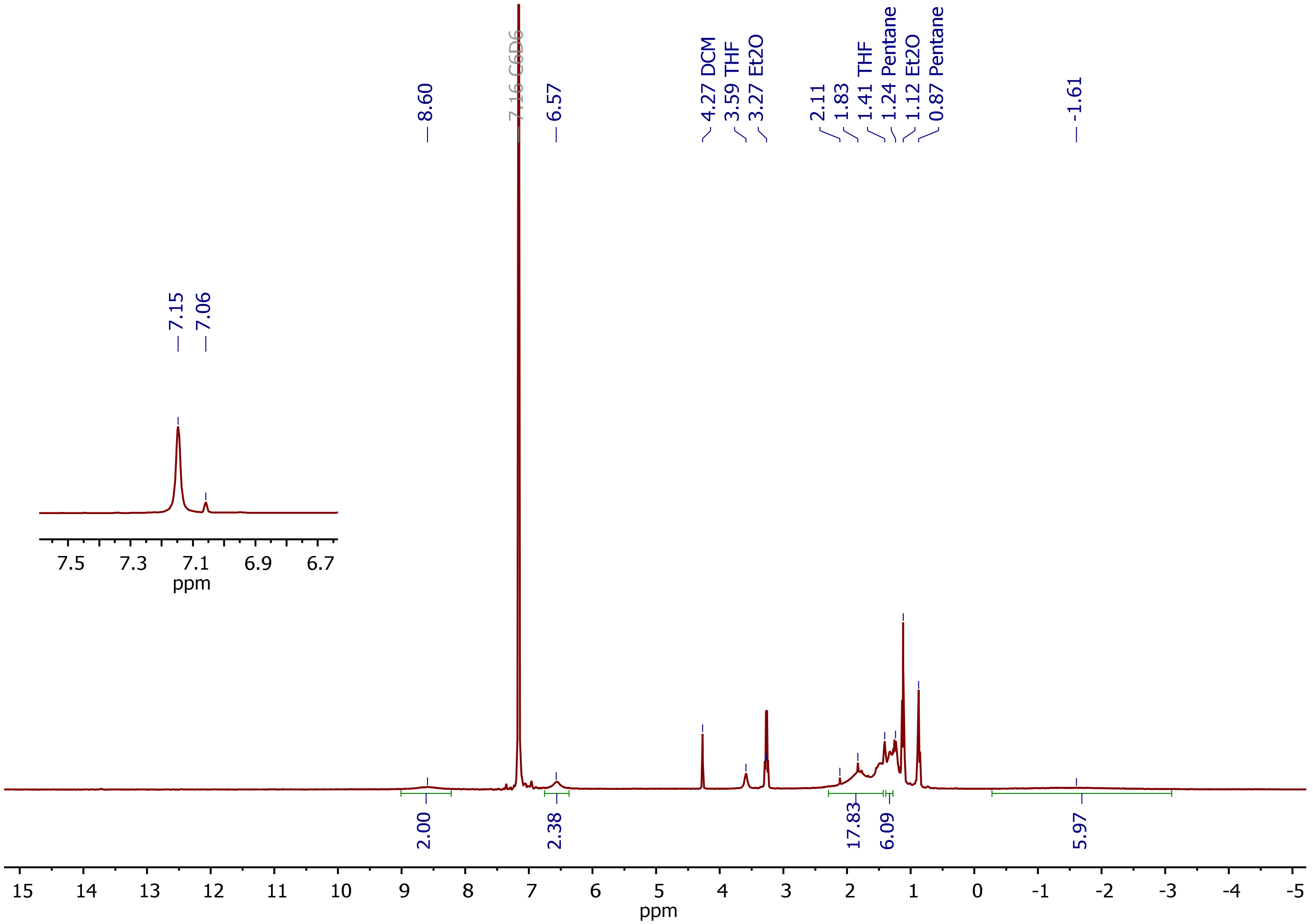

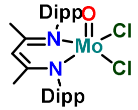

*Figure S 1: ^{1}H NMR of **1** in C_6D_6 at 298 K (10 mg). The inlay shows the NMR shift from the Evans Method.*

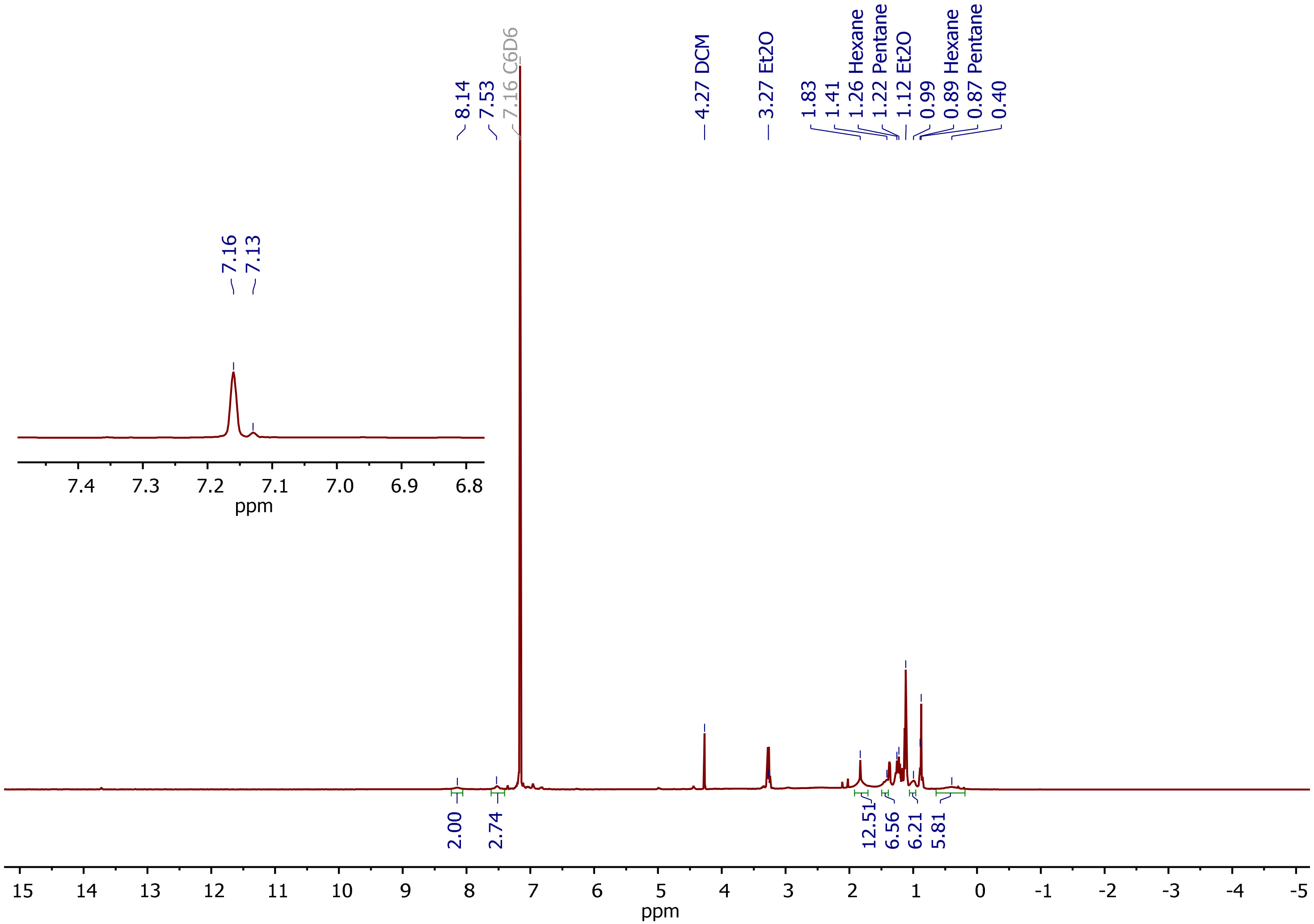

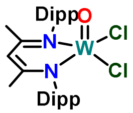

*Figure S 2: ^{1}H NMR of **2** in C_6D_6 at 298 K (10 mg). The inlay shows the NMR shift from the Evans Method.*

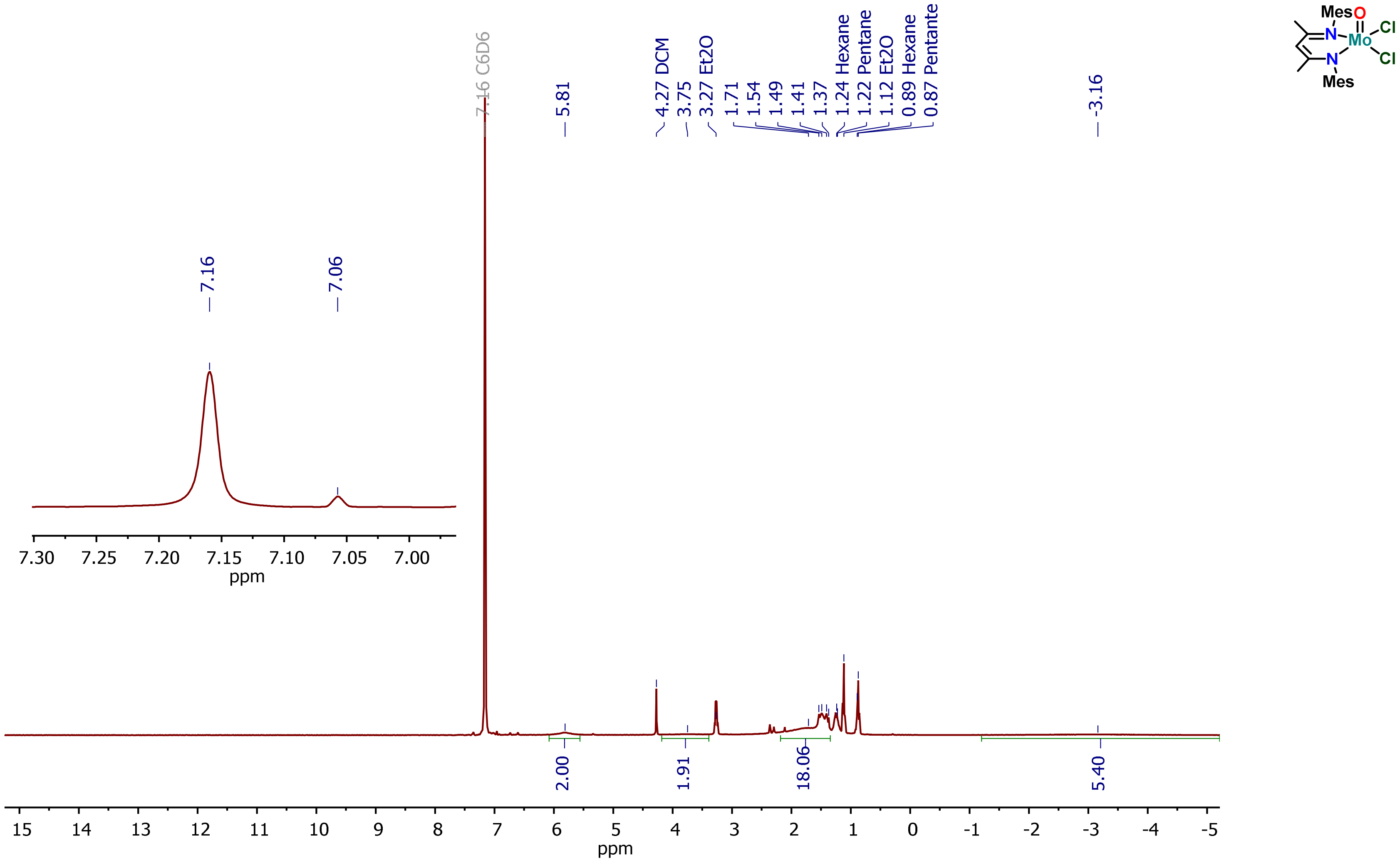

*Figure S 3: ^{1}H NMR of **3** in C_6D_6 at 298 K (12 mg). The inlay shows the NMR shift from the Evans Method.*

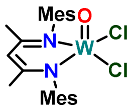

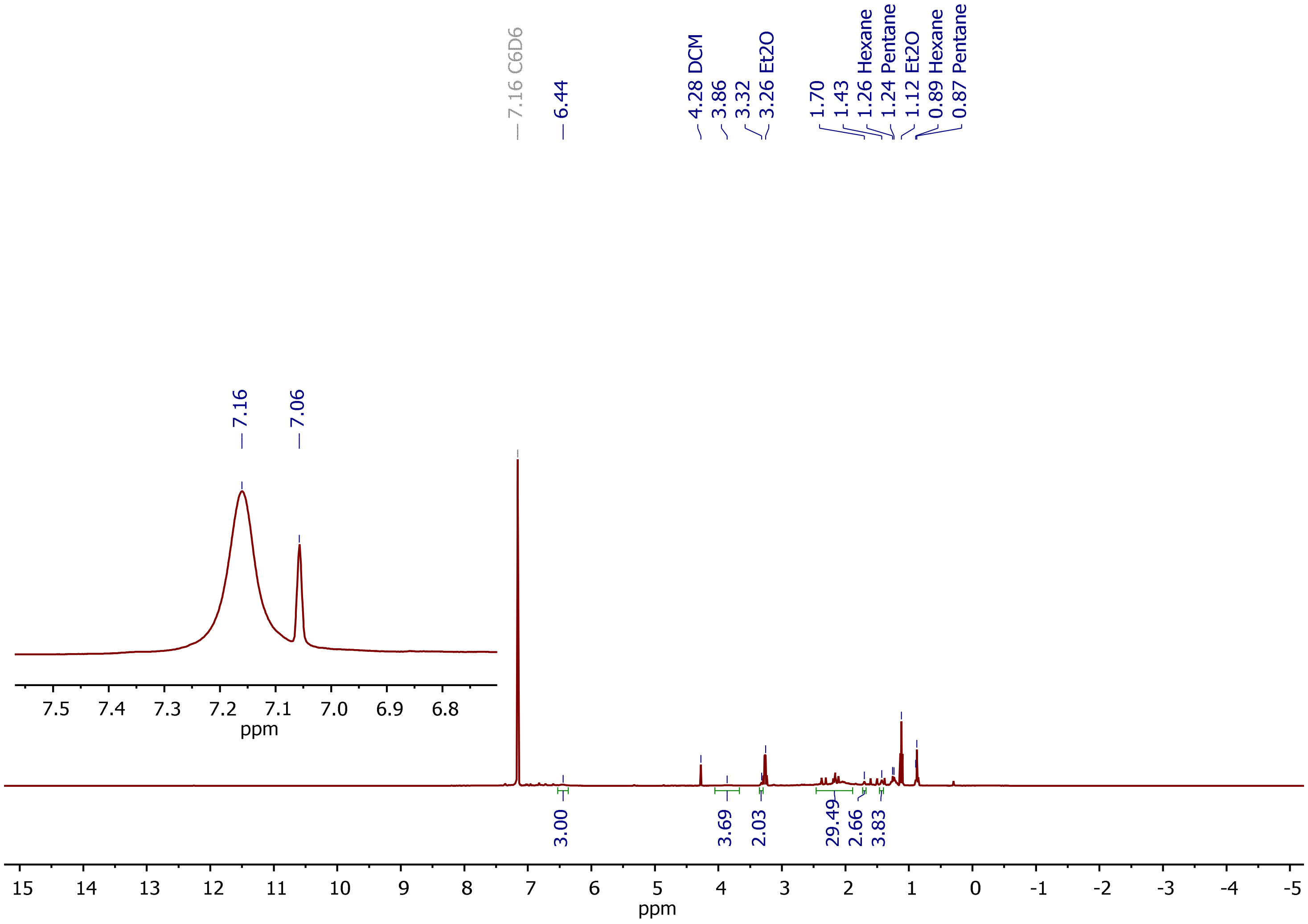

*Figure S 4: ^{1}H NMR of **4** in C_6D_6 at 298 K (13 mg). The inlay shows the NMR shift from the Evans Method.*

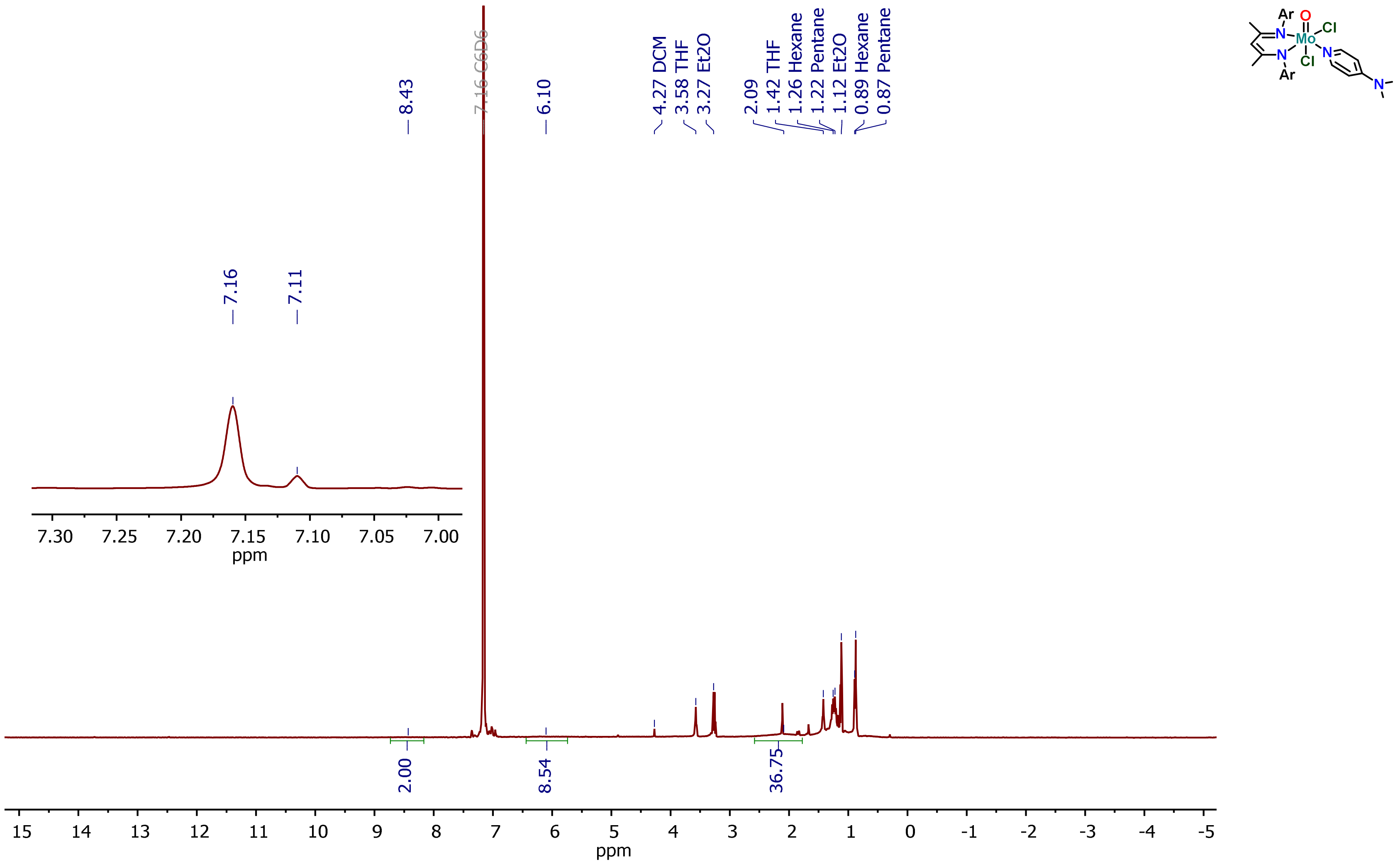

*Figure S 5: ^{1}H NMR of **5** in C_6D_6 at 298 K (11 mg). The inlay shows the NMR shift from the Evans Method.*

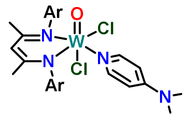

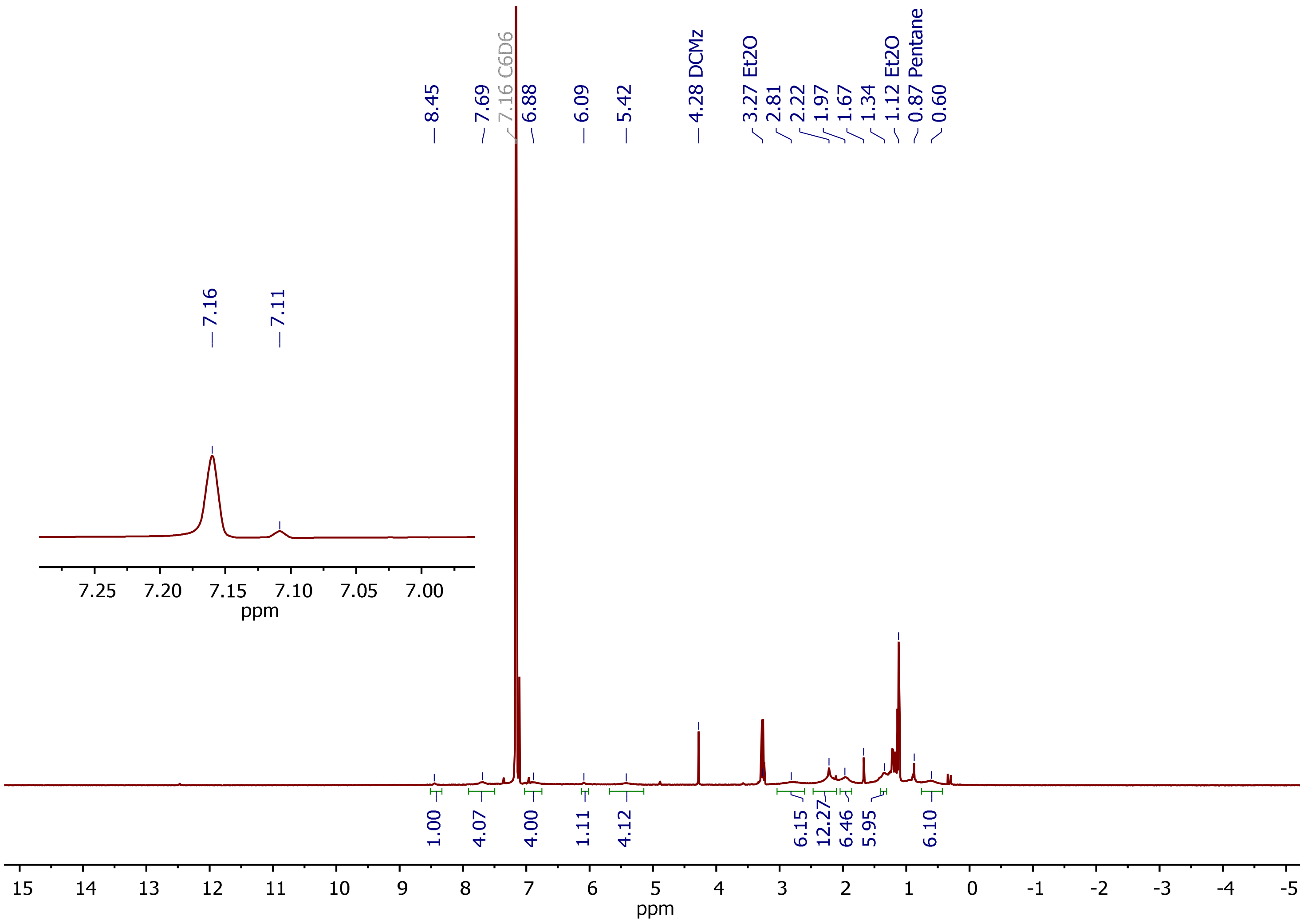

*Figure S 6:^{1}H NMR of **6** in C_6D_6 at 298 K (11 mg). The inlay shows the NMR shift from the Evans Method.*

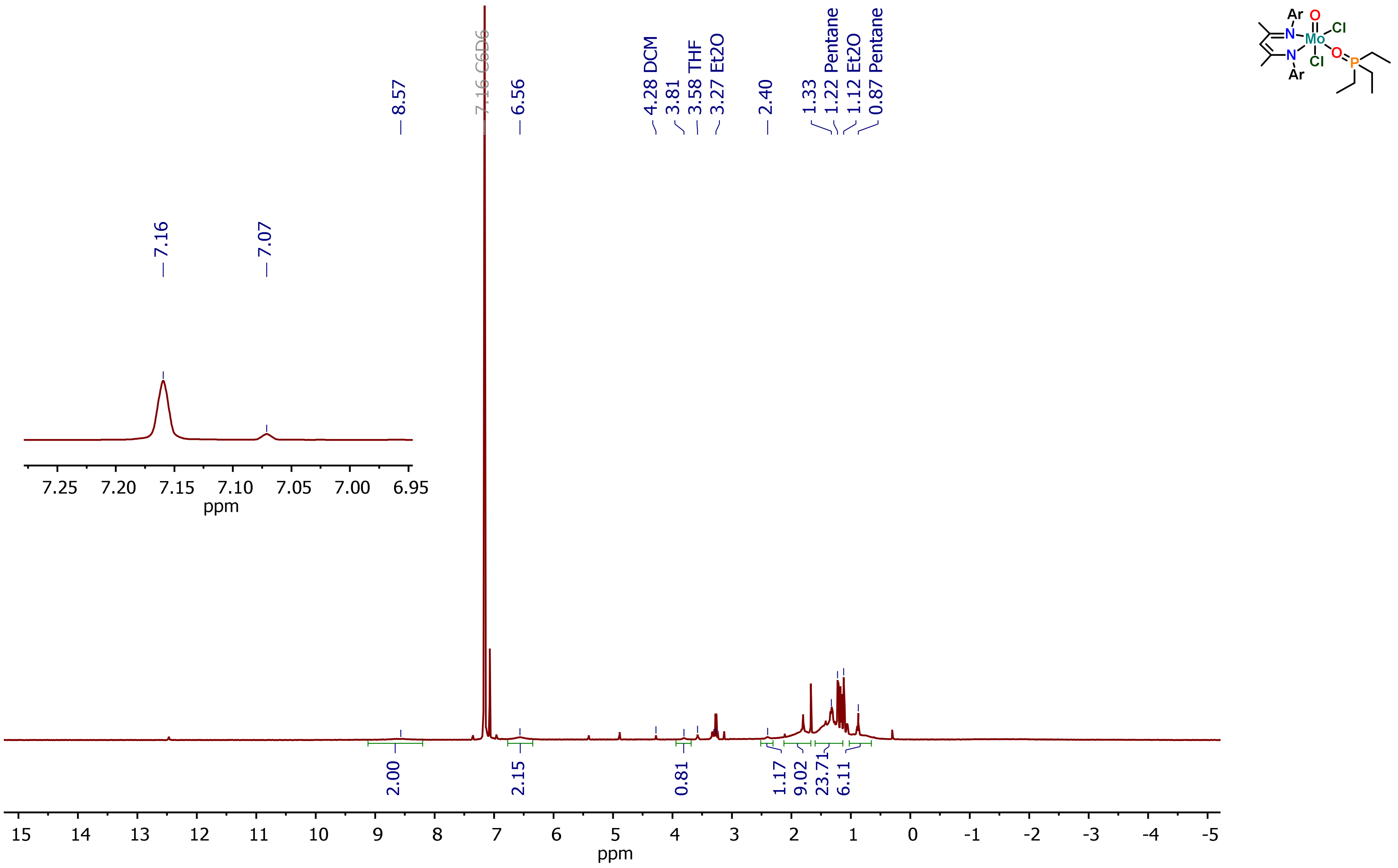

*Figure S 7: ^{1}H NMR of **7** in C_6D_6 at 298 K (9 mg). The inlay shows the NMR shift from the Evans Method. Minor impurity of H[BDI] at 12.47 and 4.88 ppm are present.*

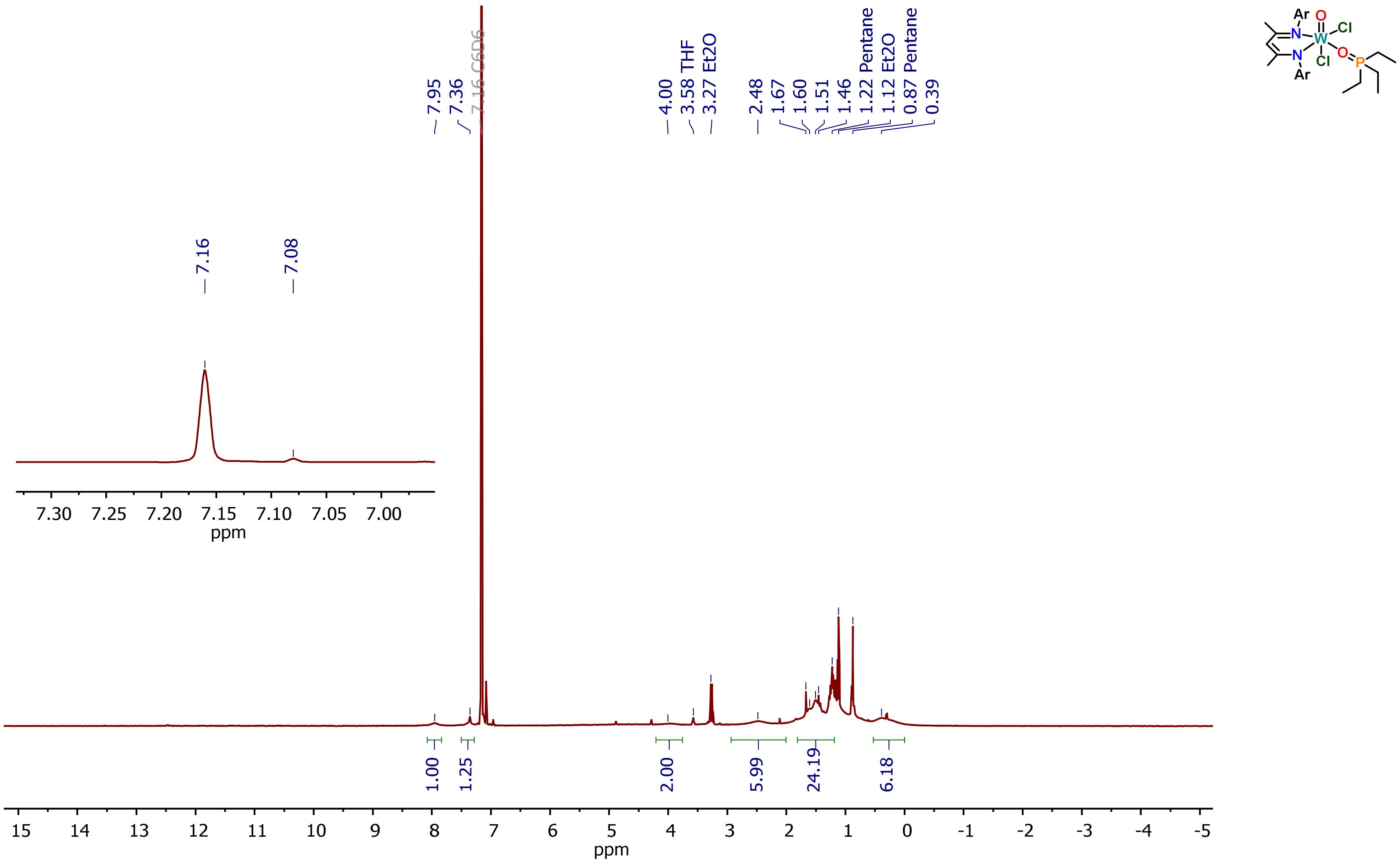

*Figure S 8: ^{1}H NMR of **8** in C_6D_6 at 298 K (10 mg). The inlay shows the NMR shift from the Evans Method. Minor impurity of H[BDI] at 12.47 and 4.88 ppm are present.*

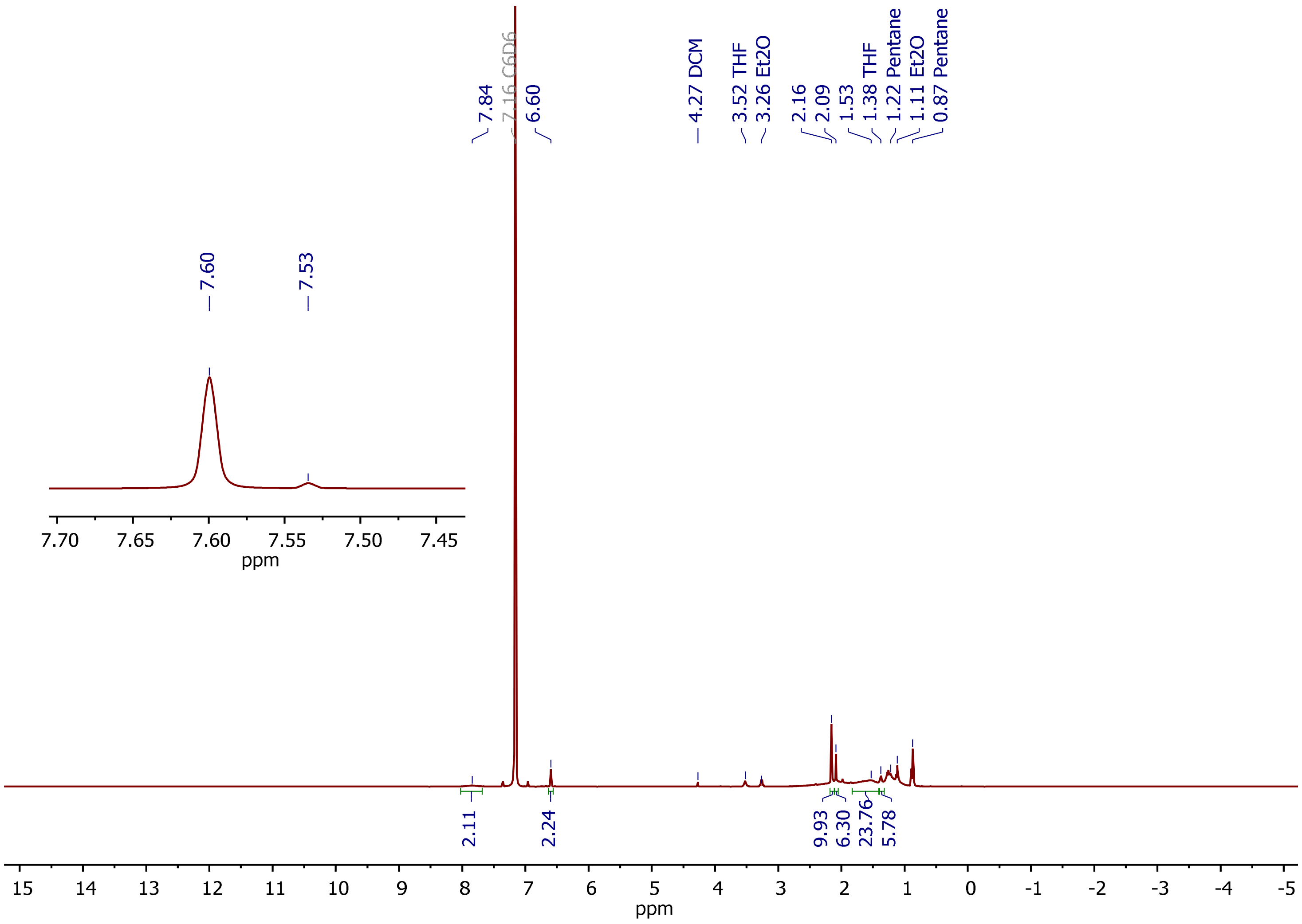

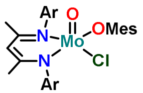

*Figure S 9: ^{1}H NMR of **9** in C_6D_6 at 298 K (12 mg). The inlay shows the NMR shift from the Evans Method.*

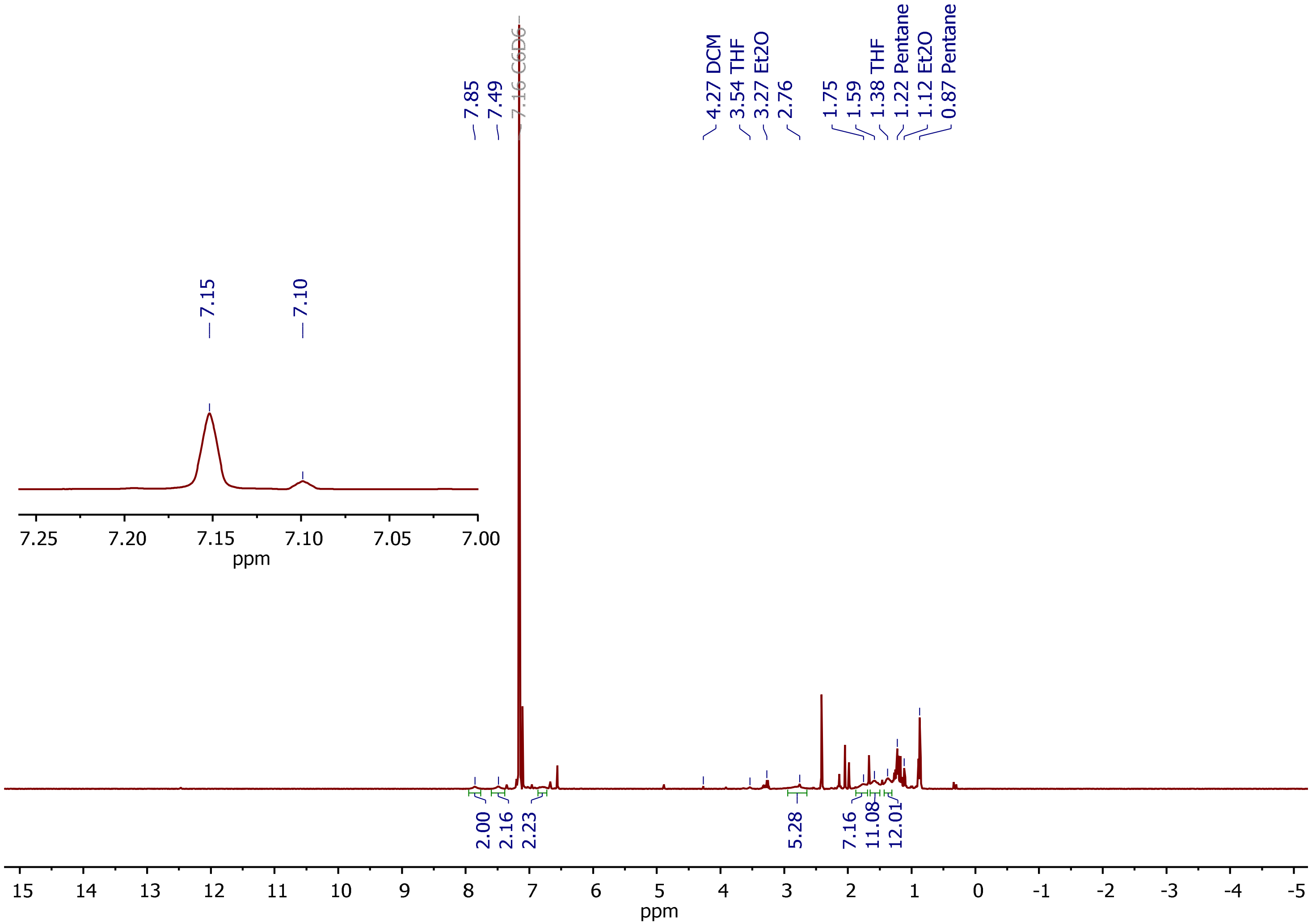

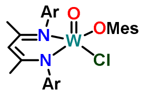

*Figure S 10: ^{1}H NMR of **10** in C_6D_6 at 298 K (12 mg). The inlay shows the NMR shift from the Evans Method.*

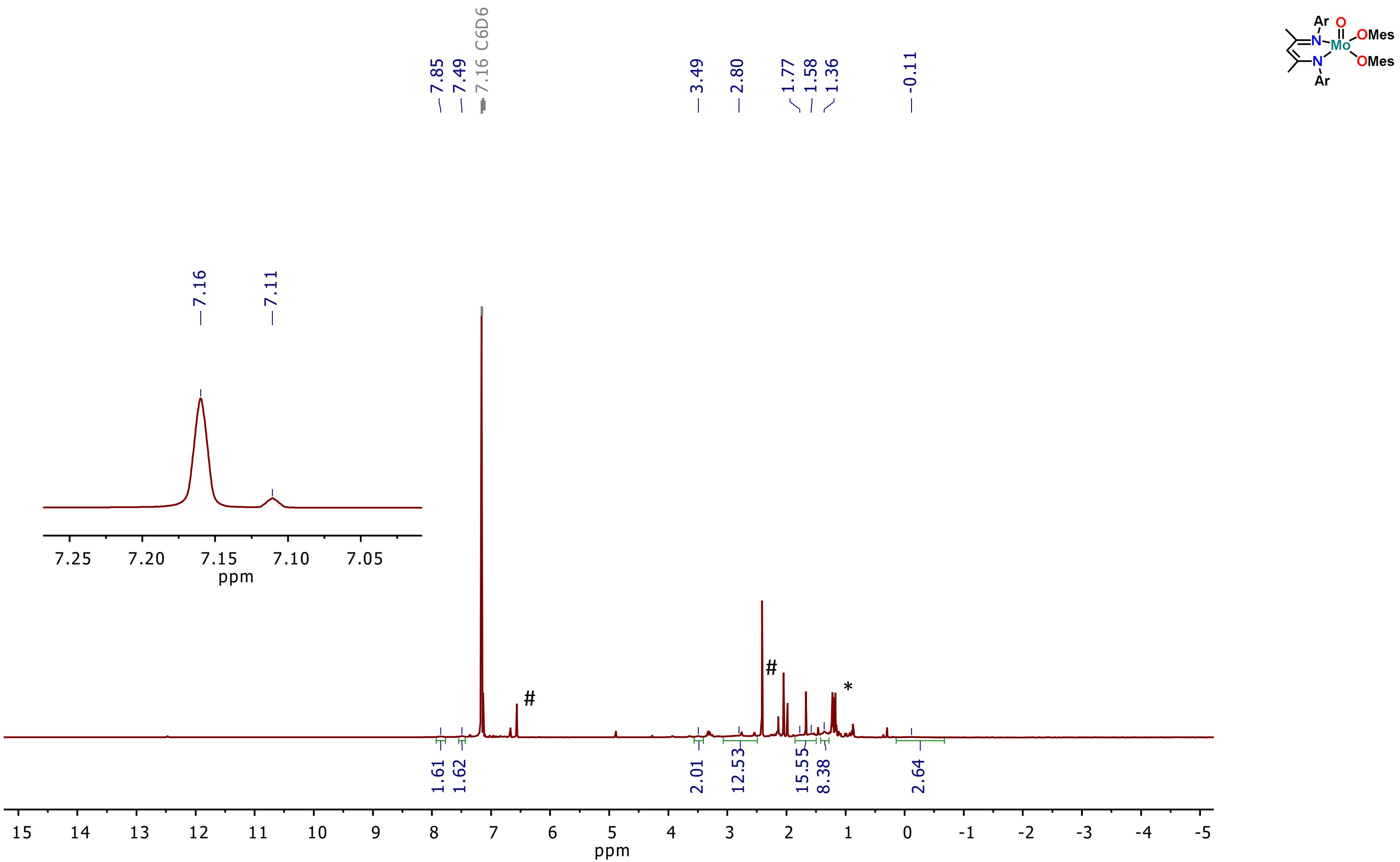

*Figure S 11: ^{1}H NMR of **11** in C_6D_6 at 298 K (11 mg). The inlay shows the NMR shift from the Evans Method. Resonances at 6.52, 2.41 and 2.04 (#) belong to residual mesitol (2,4,6-trimethylphenol) as a residual impurity. Peaks marked with a * belong to an unknown impurity.*

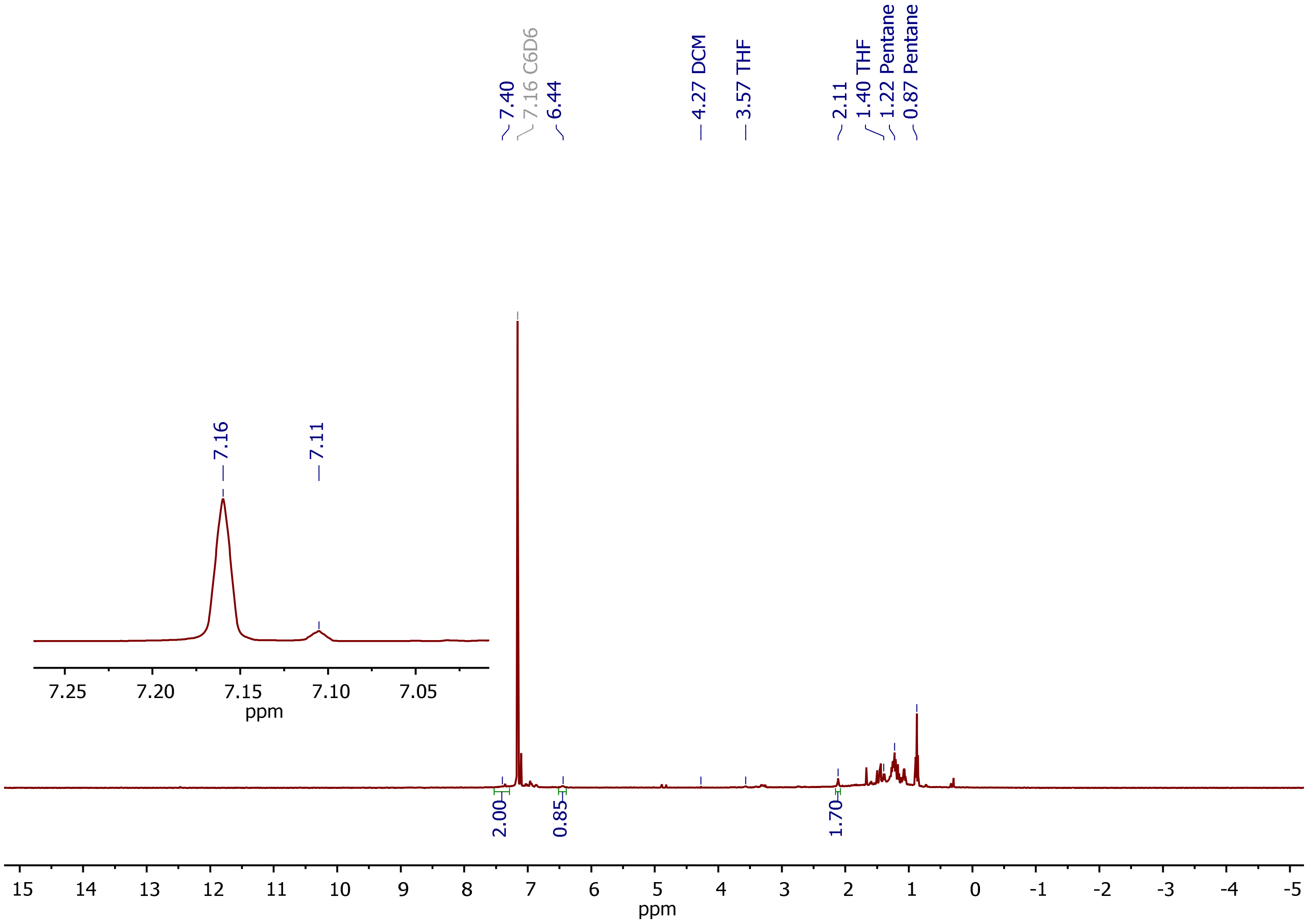

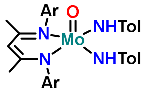

*Figure S 12: ^{1}H NMR of **12** in C_6D_6 at 298 K (12 mg). The inlay shows the NMR shift from the Evans Method. Resonances between 1.59 and 1.40 belong to an unknown and intractable impurity.*

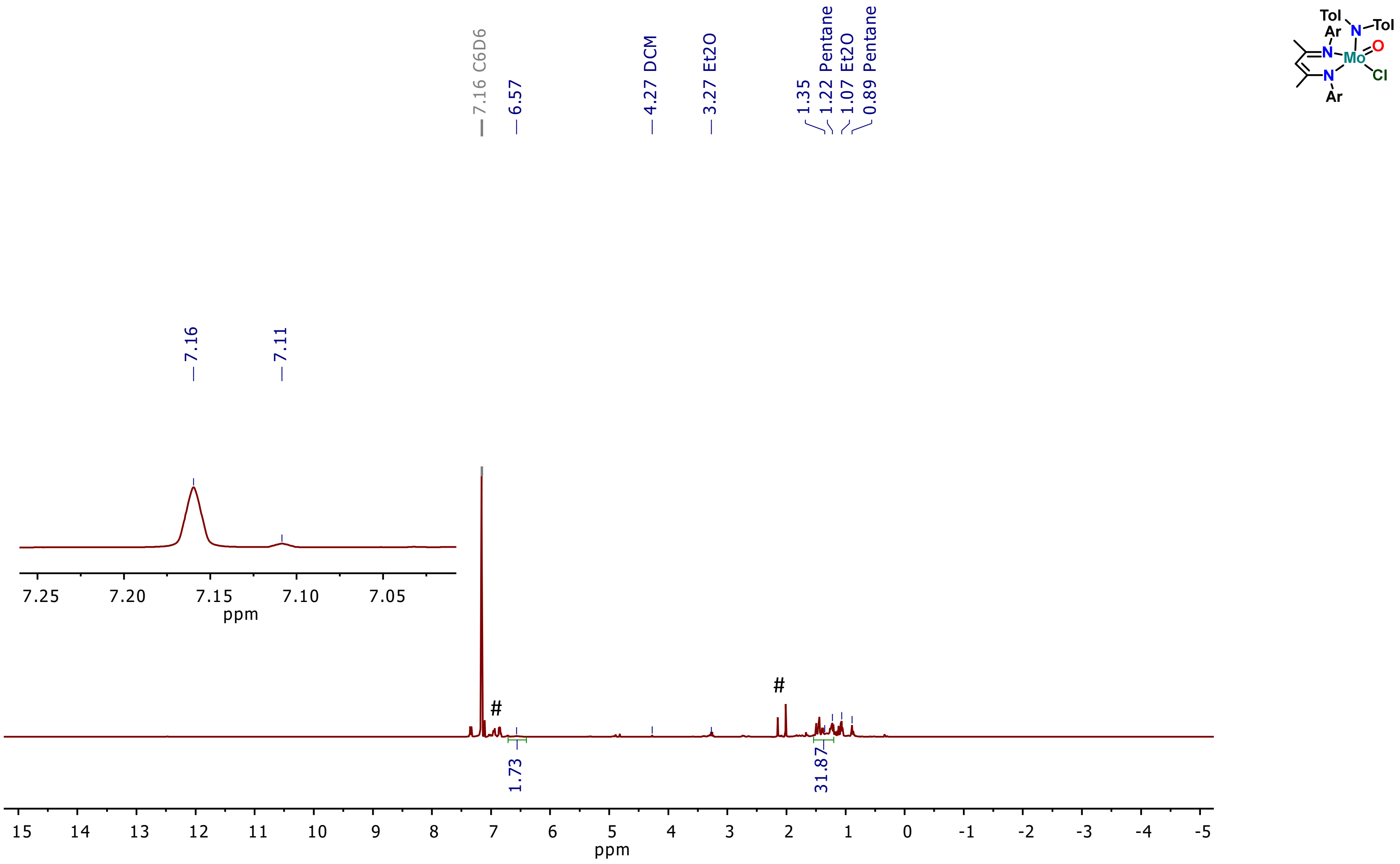

*Figure S 13: ^{1}H NMR of **13** in C_6D_6 at 298 K (11 mg). The inlay shows the NMR shift from the Evans Method. Resonances between 7.35 and 6.83 as well as 2.14 and 1.40 (#) belong to residual $HN(Tol)_2$.*

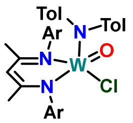

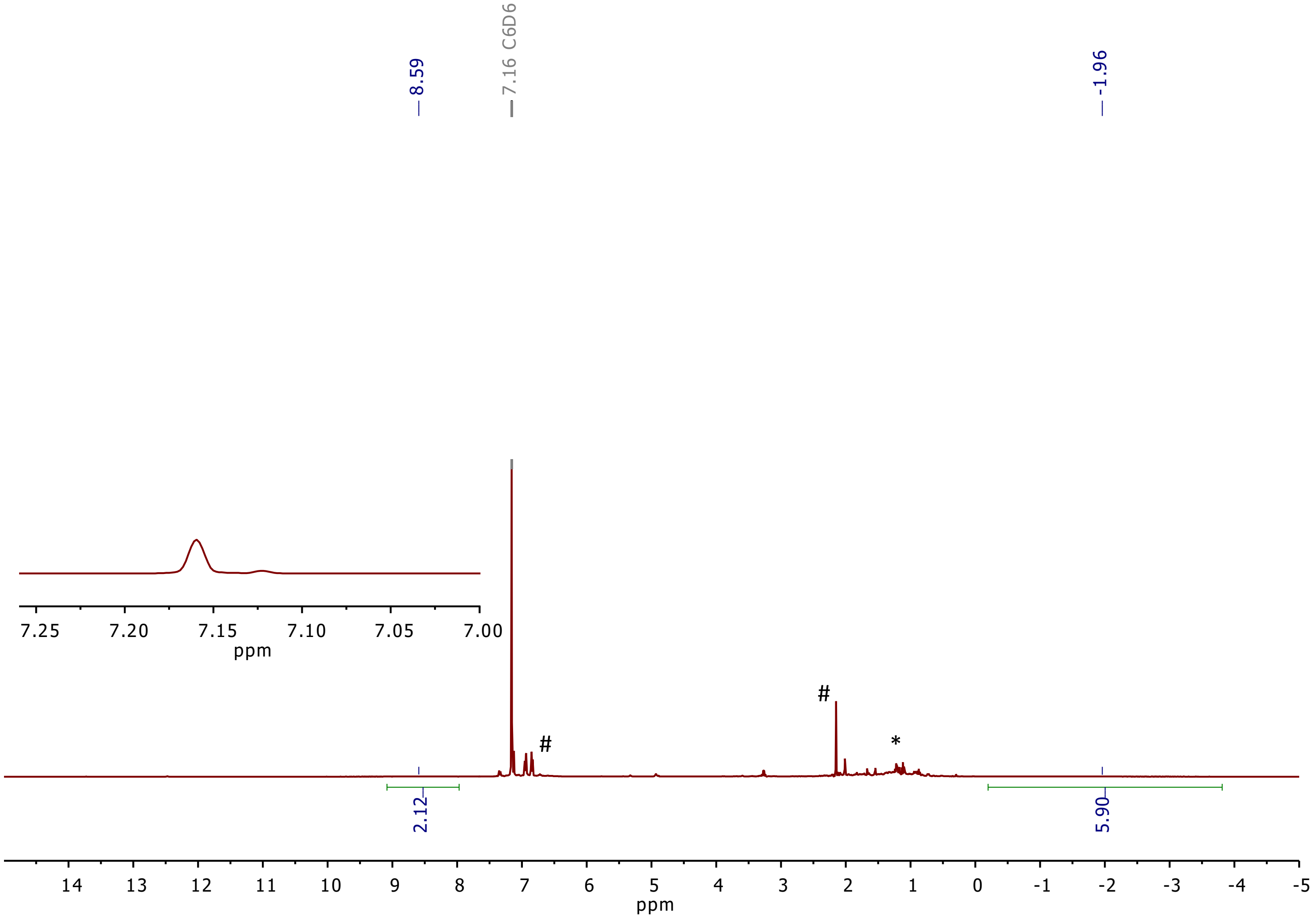

*Figure S 14 : ^{1}H NMR of **14** in C_6D_6 at 298 K (15 mg). The inlay shows the NMR shift from the Evans Method. Resonances between 7.35 and 6.83 as well as 2.14 and 1.40 (#) belong to an impurity of residual $HN(Tol)_2$, impurities marked with a * are unknown.*

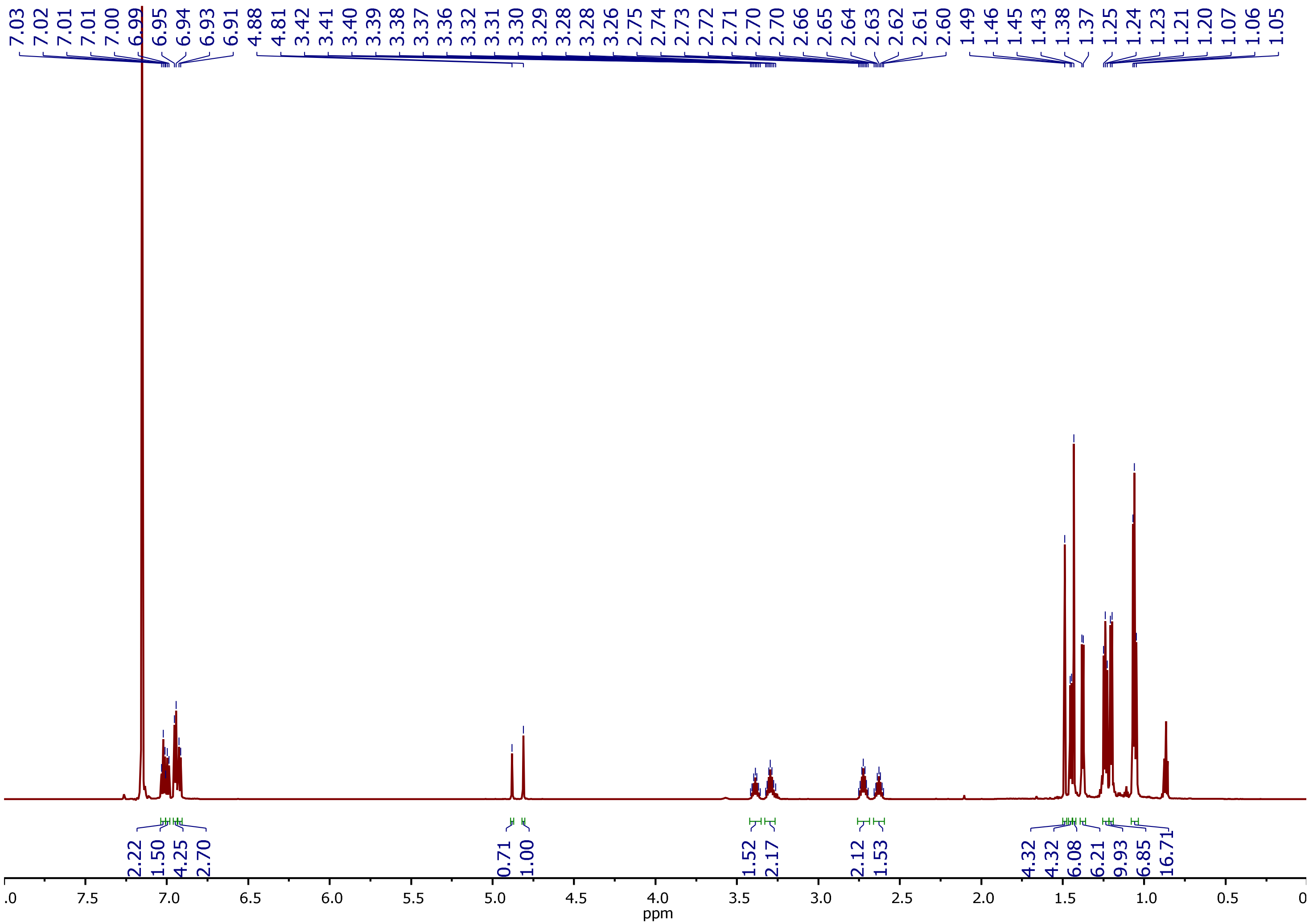

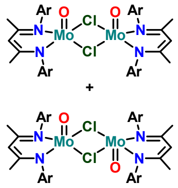

Figure S 15: ^{1}H NMR of **15-*cis*** and **15-*trans*** in C_6D_6 at 298 K.

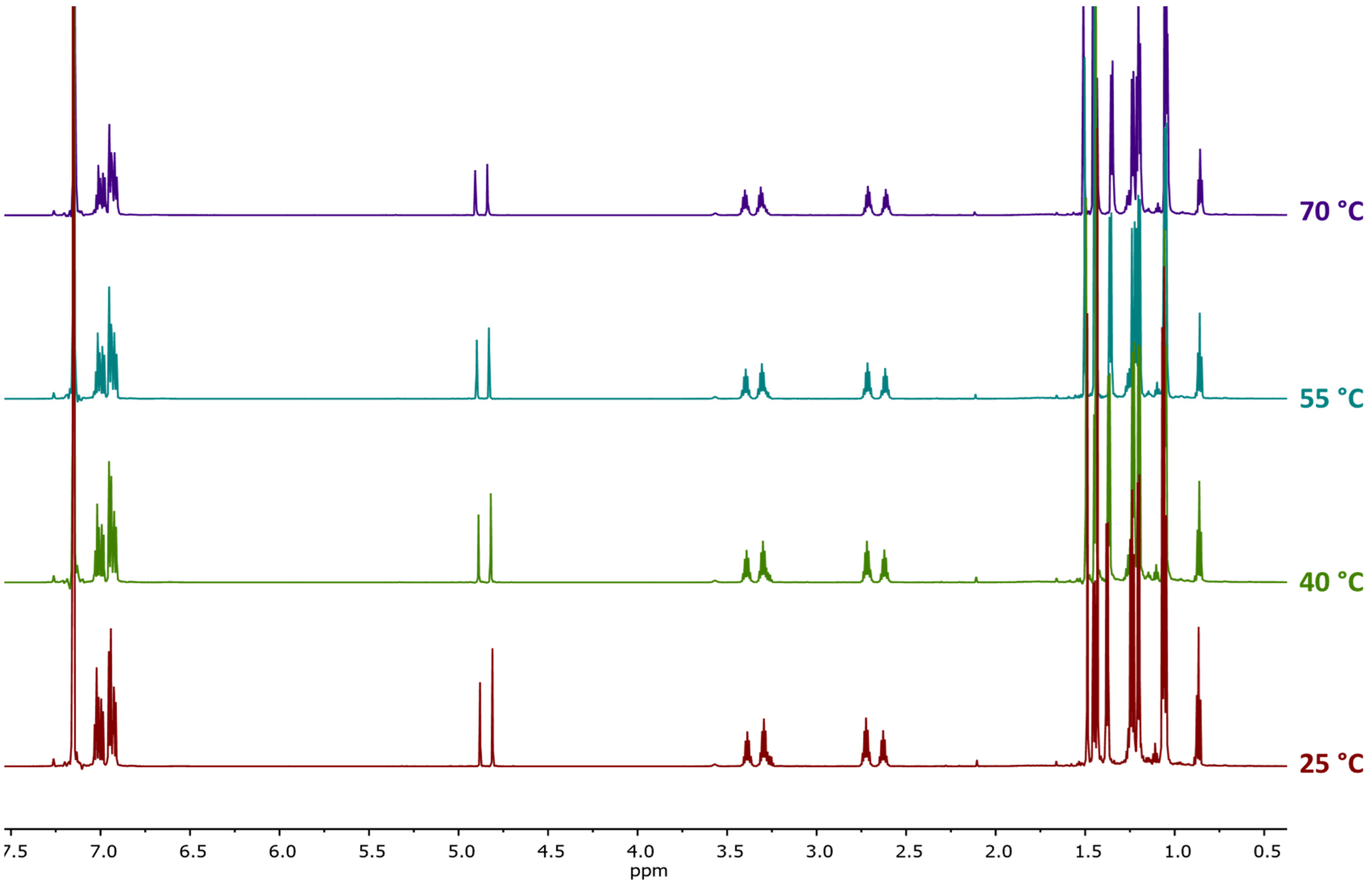

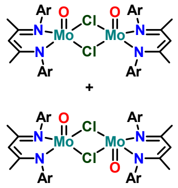

Figure S 16: 1H VT NMR of complex **15** between 25 and 70 °C (298 – 343 K) in C_6D_6.

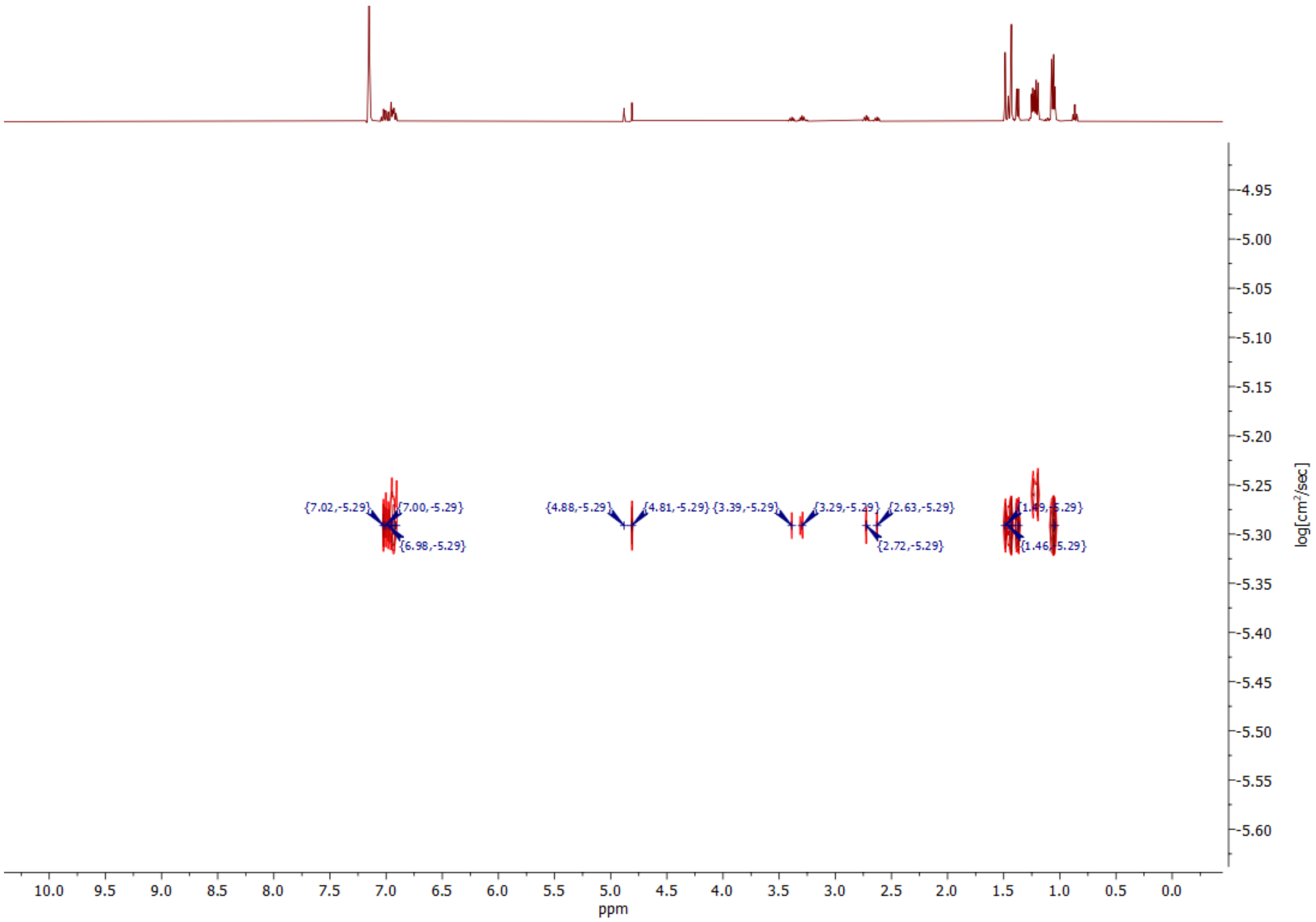

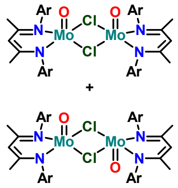

Figure S 17: ^{1}H-DOSY NMR of **15** in C_6D_6 at 298 K. The signals at 5.25 log[cm^2/s] belong to an impurity of pentane.

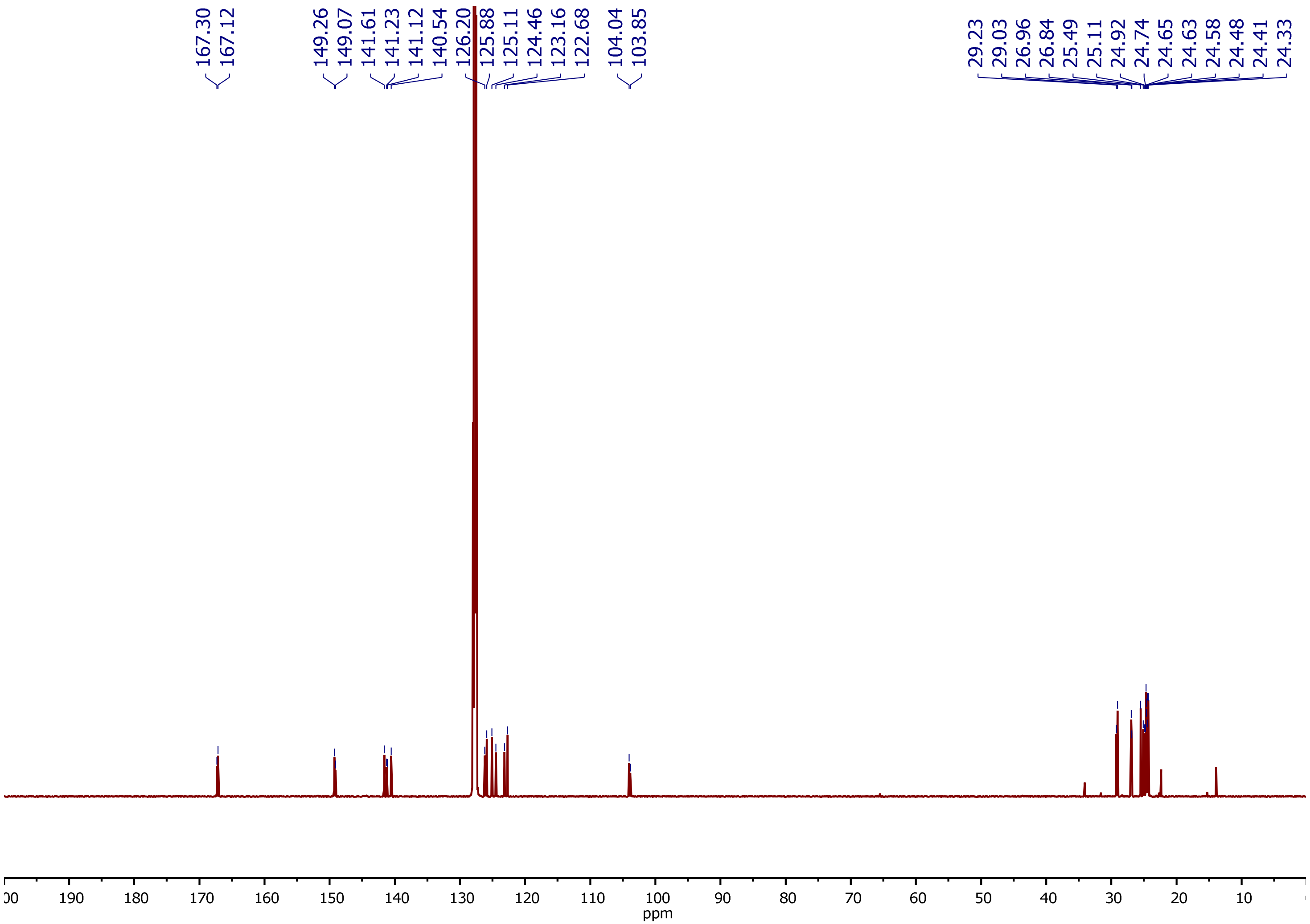

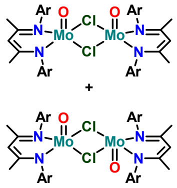

Figure S 18: ^{13}C NMR of **15** in C_6D_6 at 298 K.

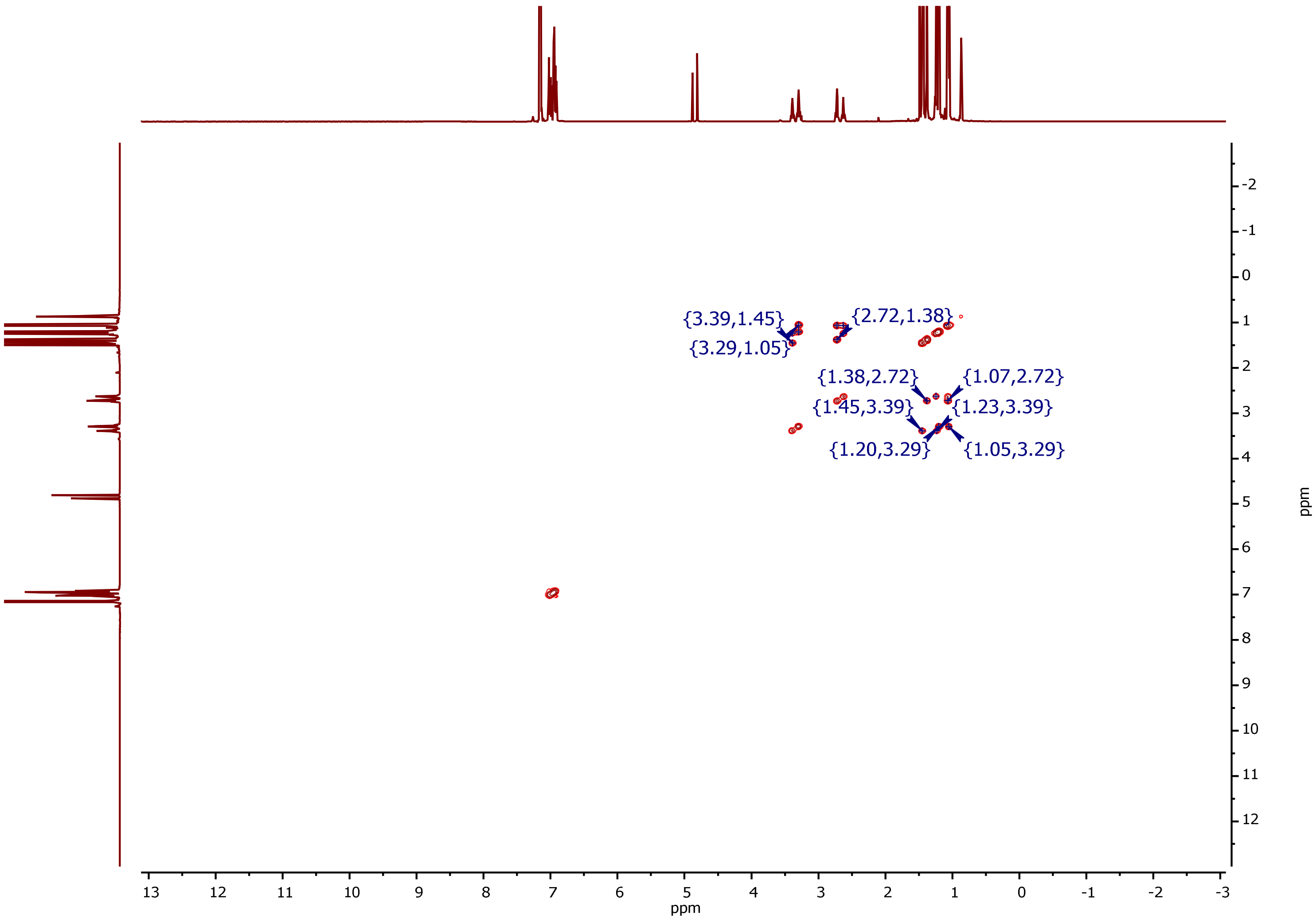

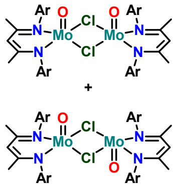

Figure S 19: ^1H-^1H COSY of **15** in C_6D_6 at 298 K.

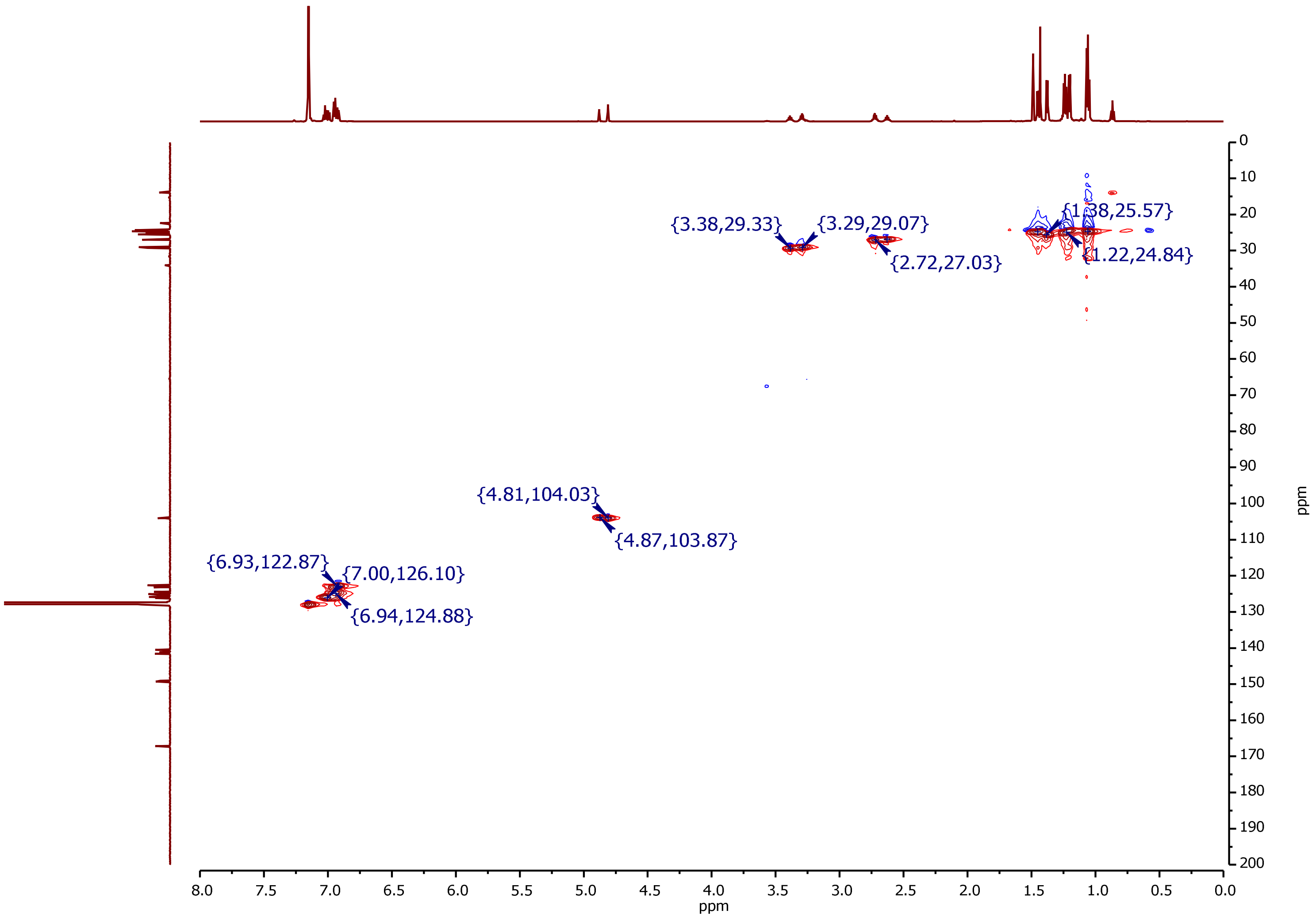

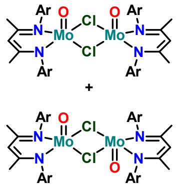

Figure S 20: ^{1}H-^{13}C HSQC of **15** in C_6D_6 at 298 K.

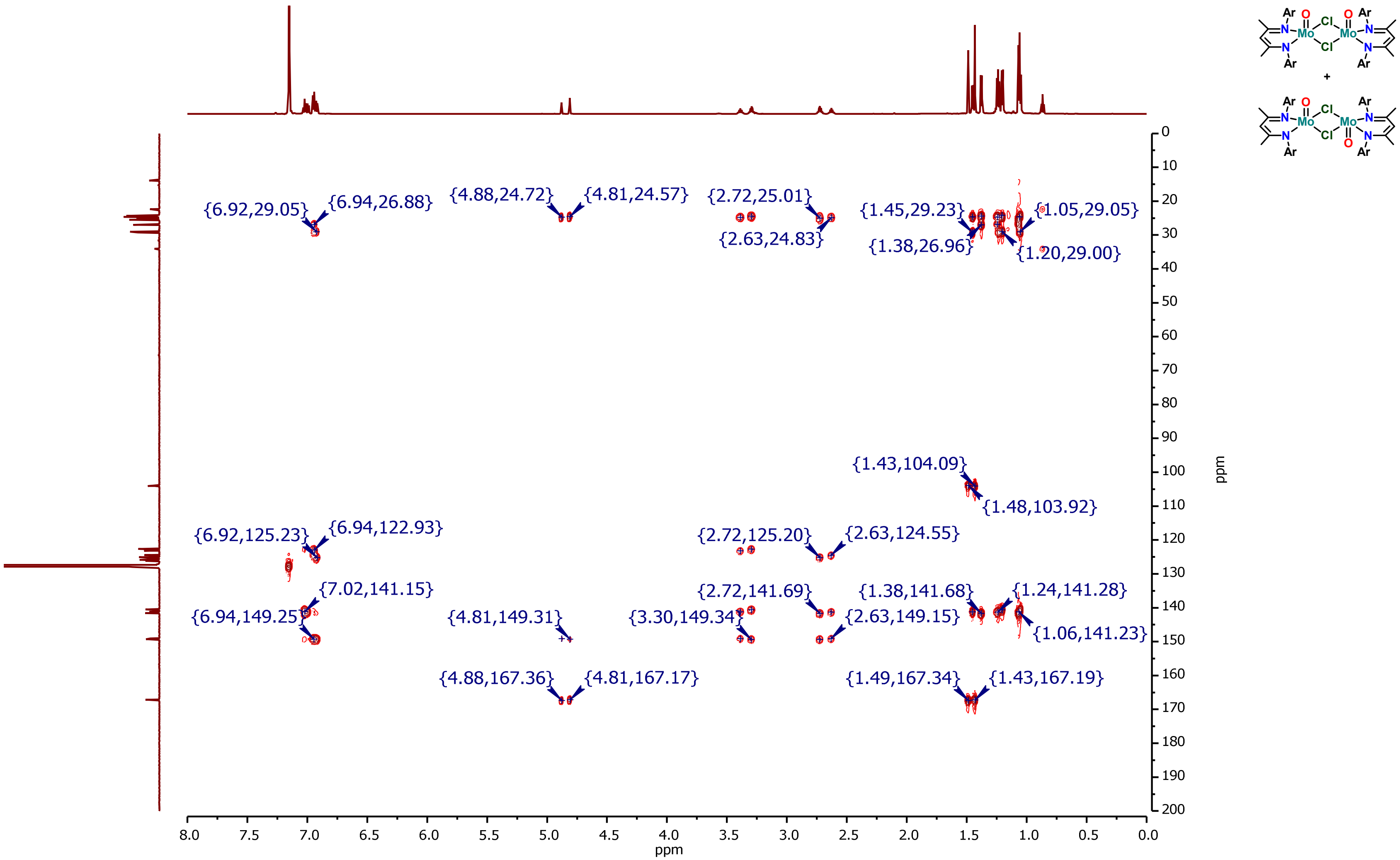

Figure S 21: ^{1}H-^{13}C HMBC of **15** in C_6D_6 at 298 K.

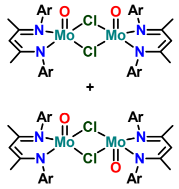

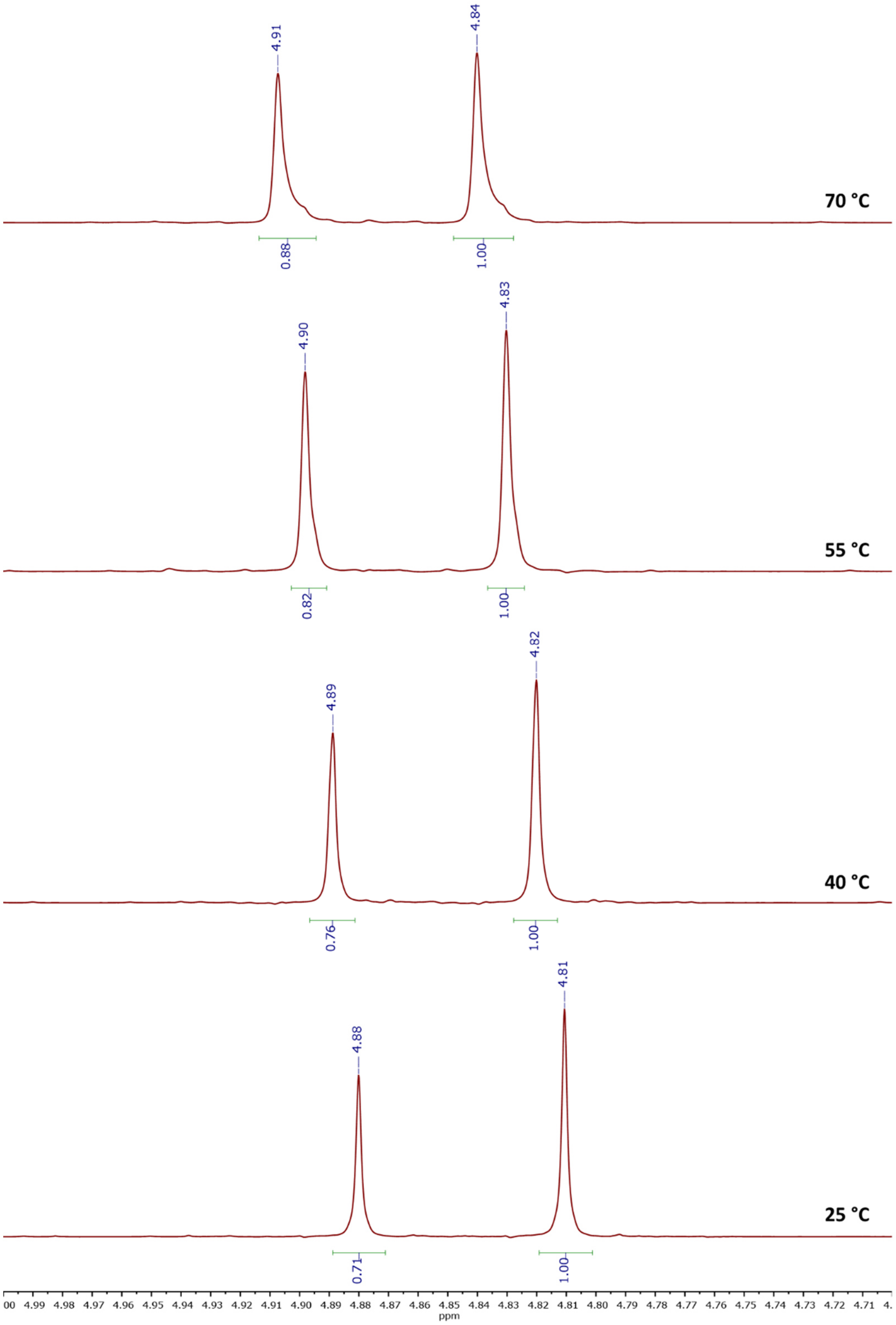

Figure S 22: Ratios of [1]H-BDI backbone ligand integrals over temperature range between 298 – 343 K to determine the ratio between **15-cis** and **15-trans** in solution.

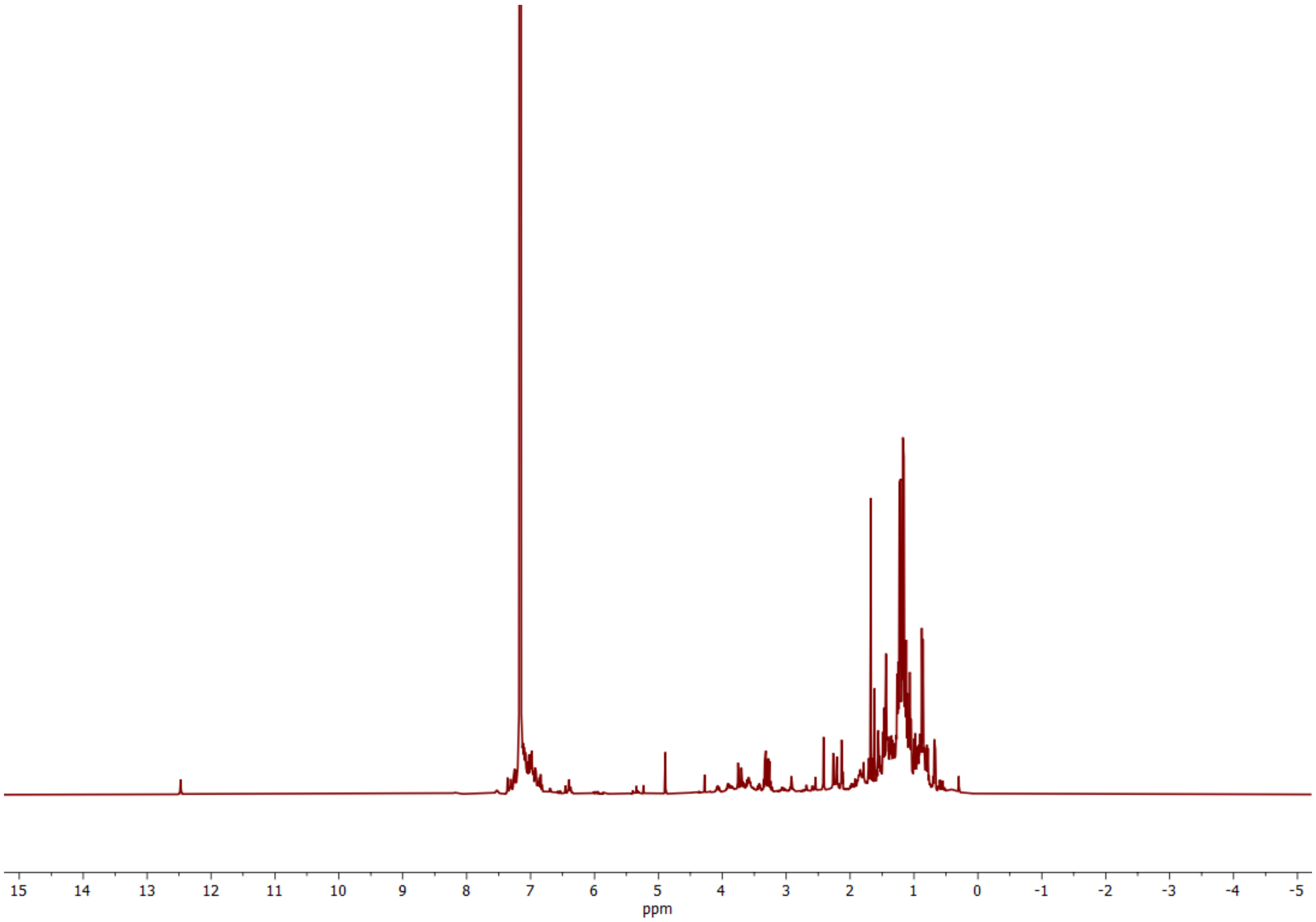

Figure S 23: ^1H NMR of the attempted reduction of **2** with KC_8.

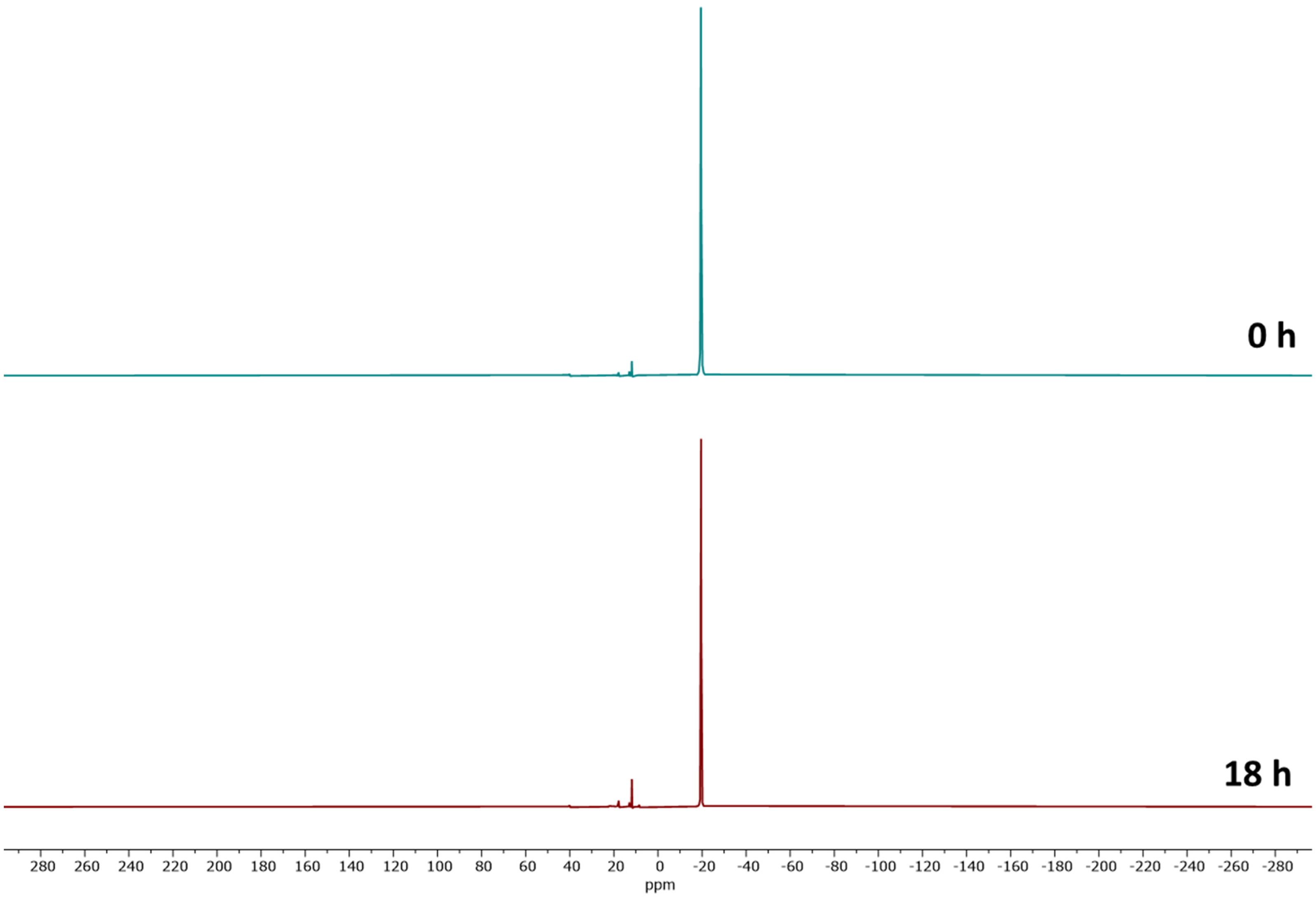

Figure S 24: Attempted deoxygenation of **1** with PEt_3 at 100 °C.

2. UV-Vis spectra

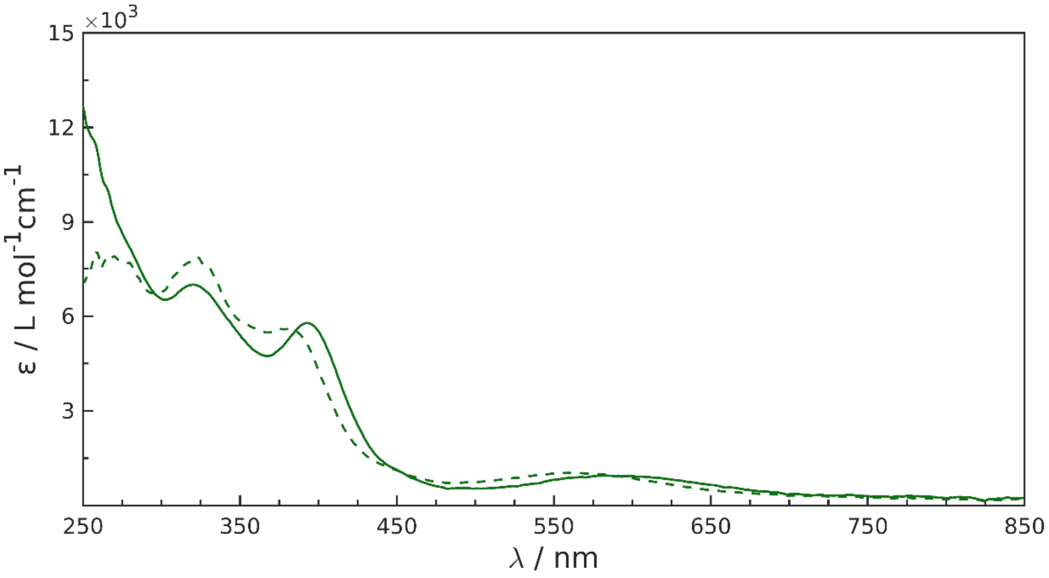

Figure S 25: UV-VIS spectrum of **1** (solid) and **3** (dashed) in Et_2O at 298 K.

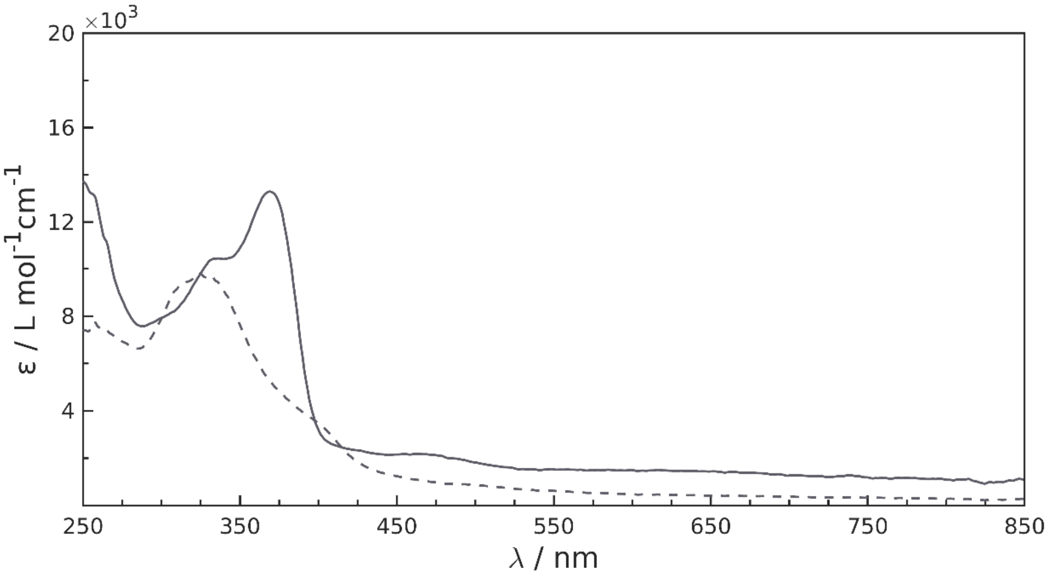

Figure S 26: UV-VIS spectrum of **2** (solid) and **4** (dashed) in Et_2O at 298 K.

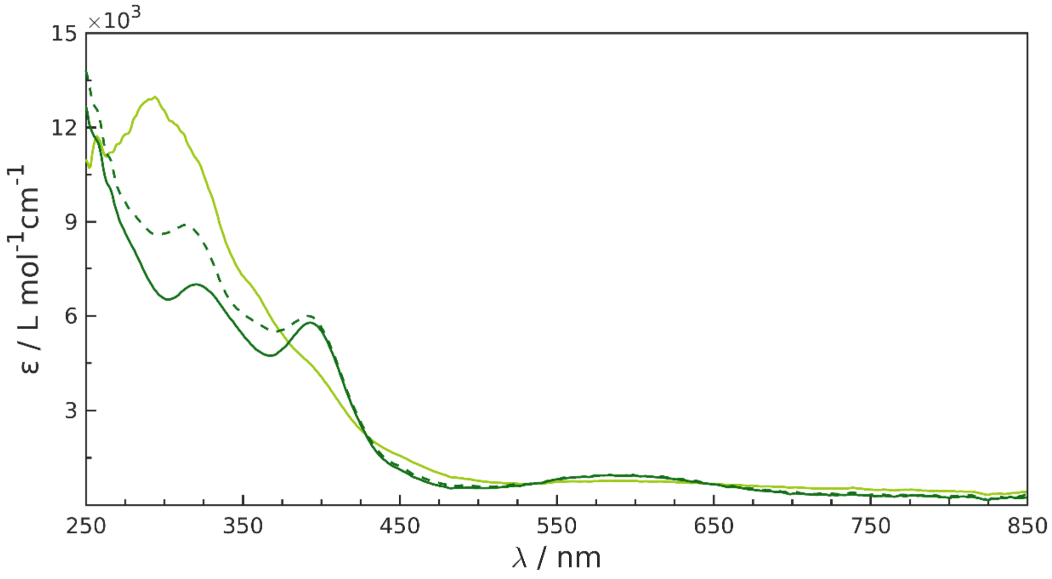

Figure S 27: UV-VIS spectrum of **5** (light green) and **7** (dark green) in Et_2O at 298 K including **1** as reference (dashed).

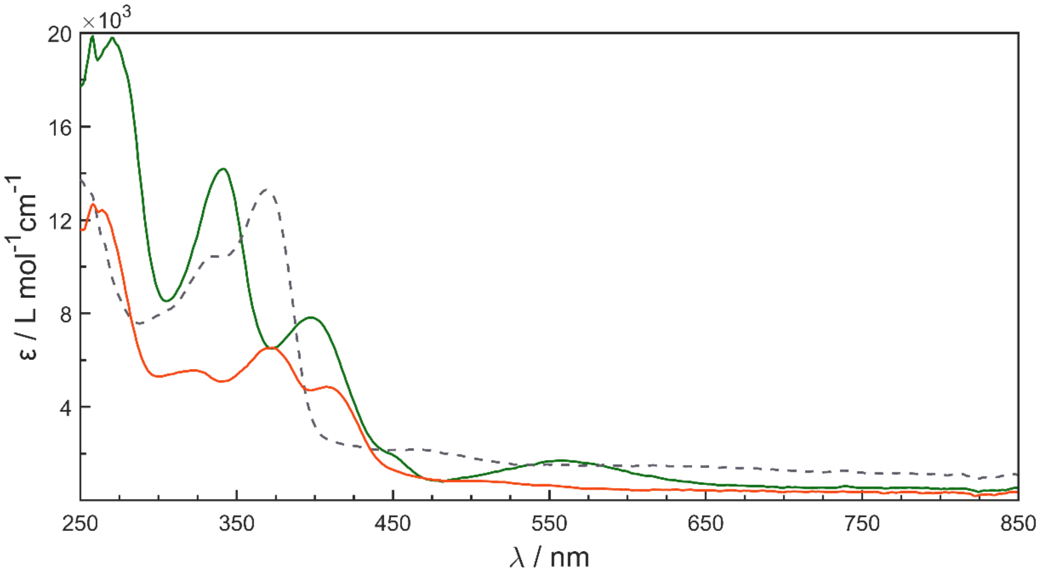

Figure S 28: UV-VIS spectrum of **6** (green) and **8** (orange) in Et_2O at 298 K including **2** as reference (dashed).

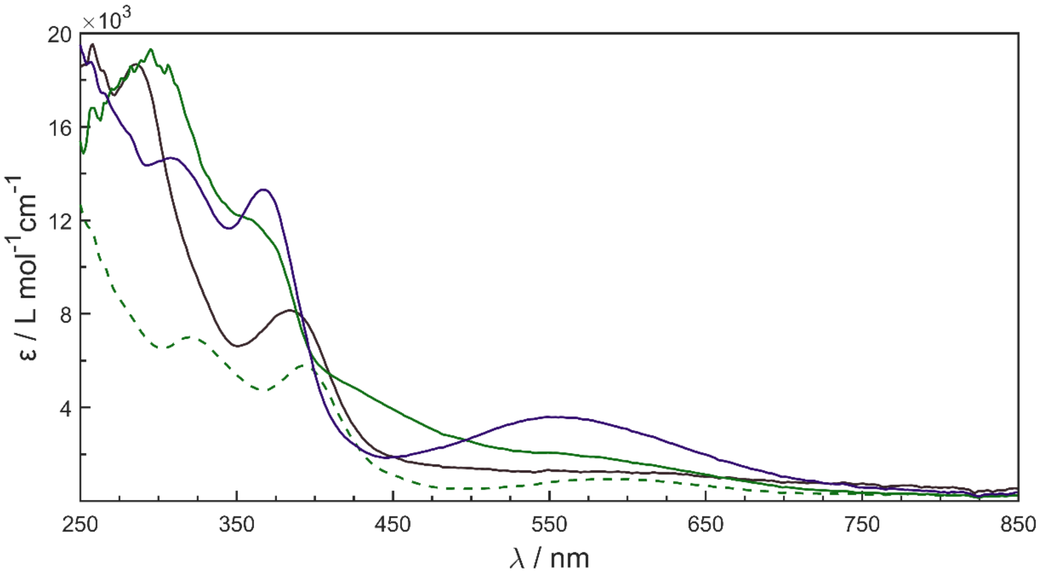

Figure S 29: UV-VIS spectrum of **9** (blue) and **12** (green) and **13** (black) in Et_2O at 298 K including **1** as reference (dashed).

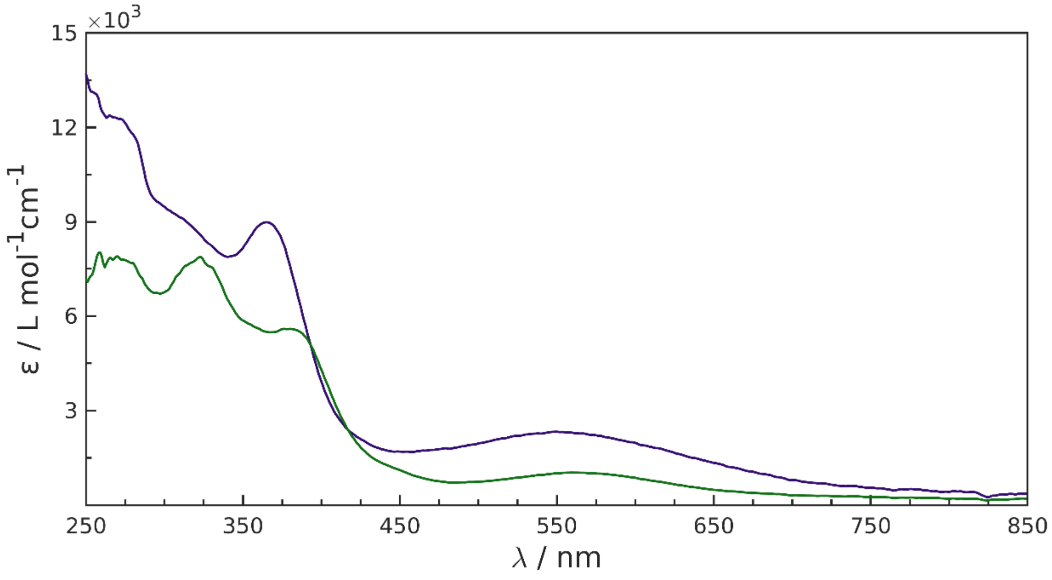

Figure S 30: UV-VIS spectrum of **11** (blue) and **3** (green) in Et_2O at 298 K.

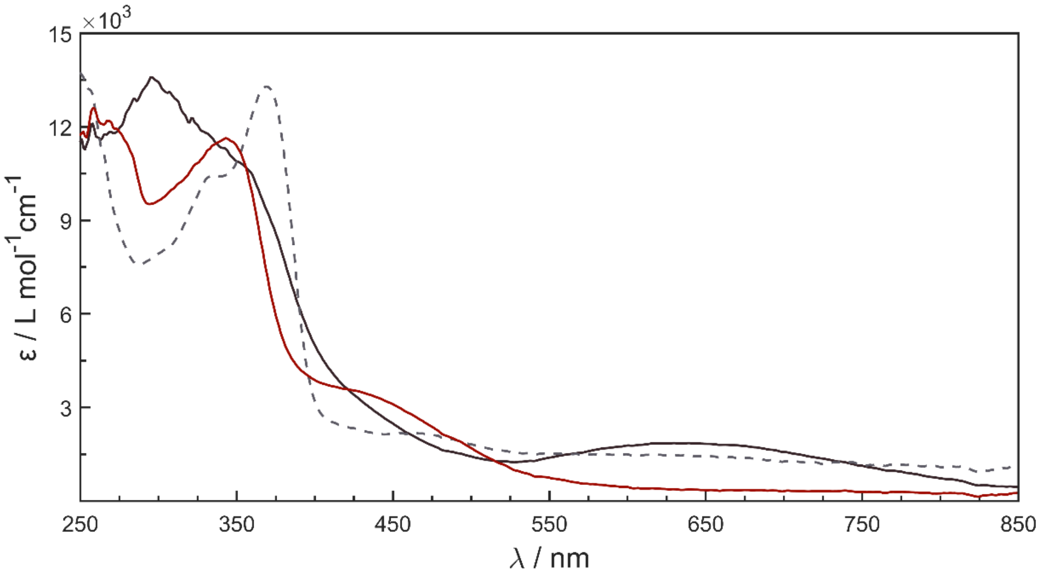

Figure S 31: UV-VIS spectrum of **10** (red) and **14** (black) in Et_2O at 298 K including **2** as reference (dashed).

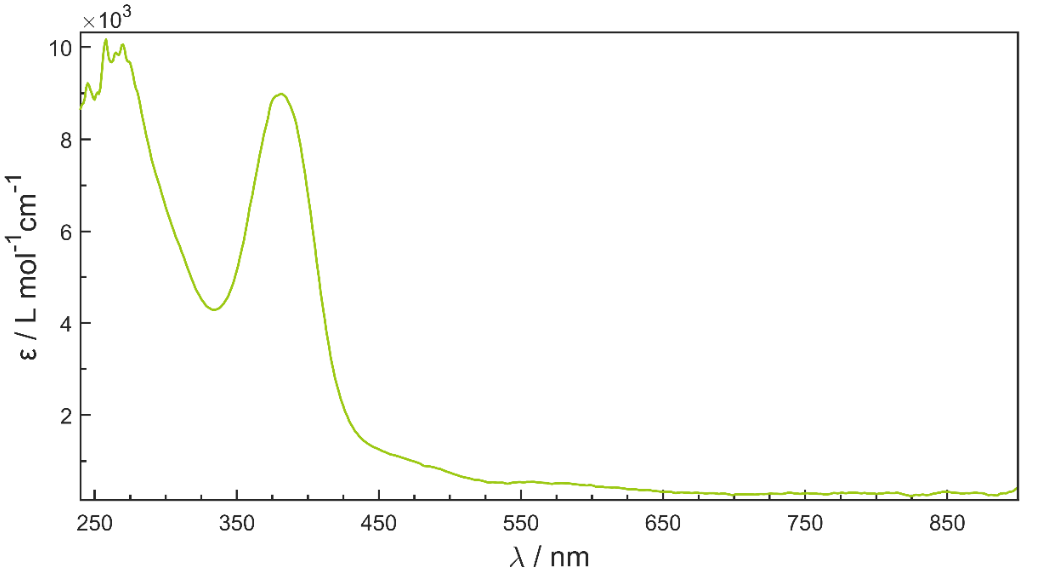

Figure S 32: UV-VIS spectrum of **15** in Et_2O at 298

3. IR spectra

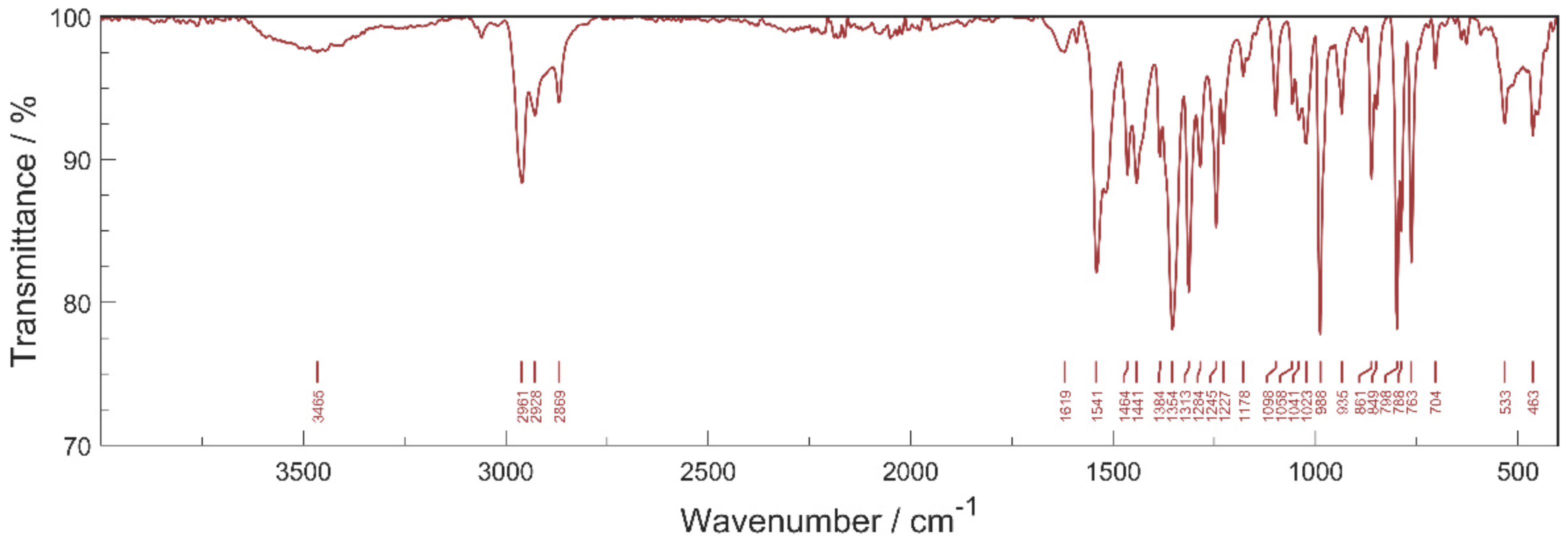

Figure S 33: ATR-IR spectrum of **1** at 298 K.

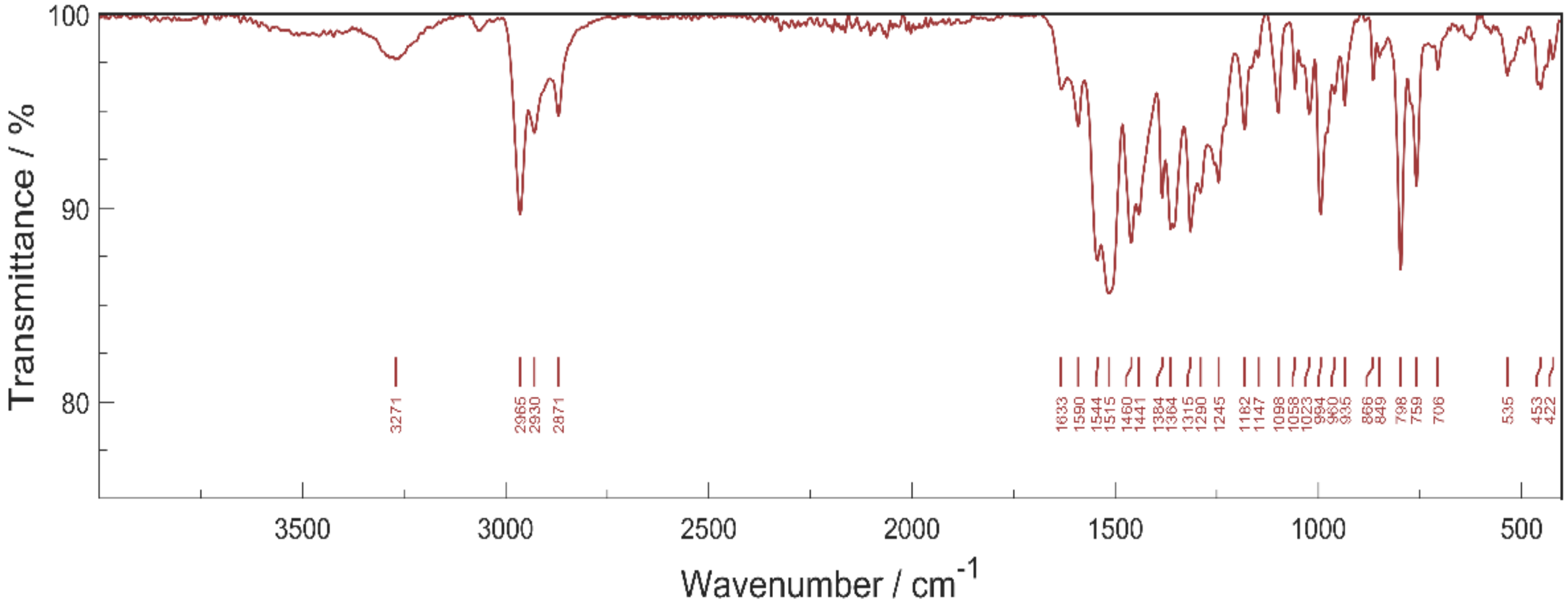

Figure S 34: ATR-IR spectrum of **2** at 298 K.

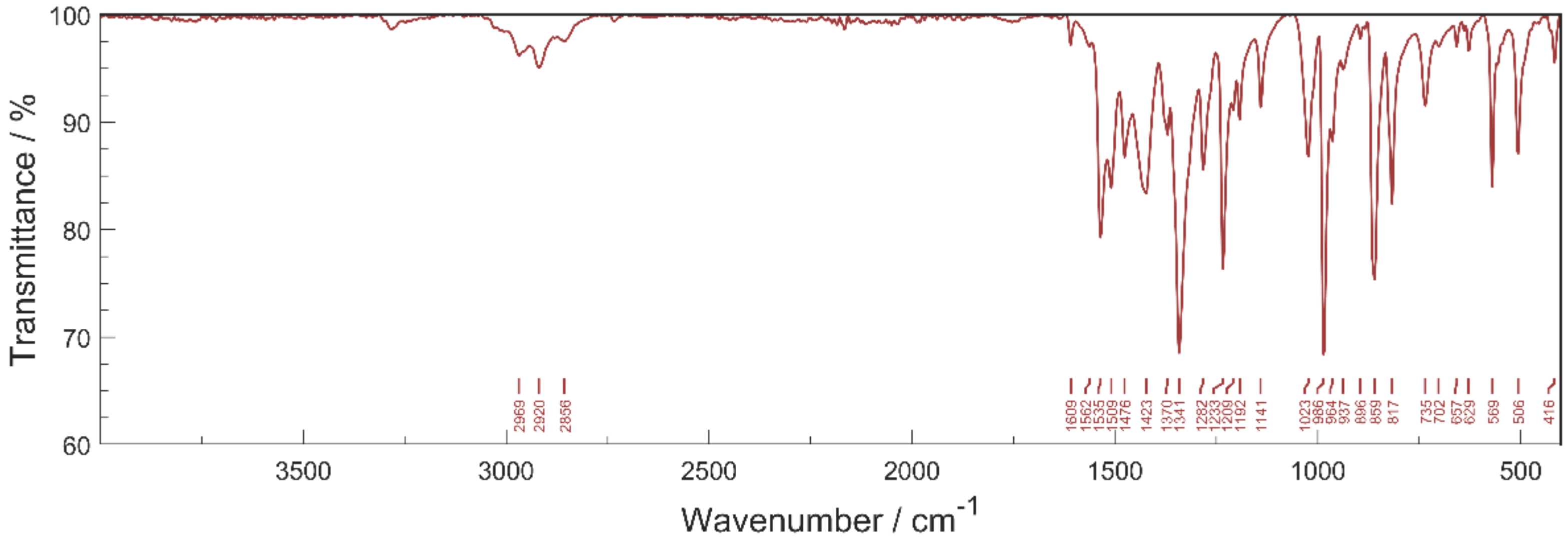

Figure S 35: ATR-IR spectrum of **3** at 298 K.

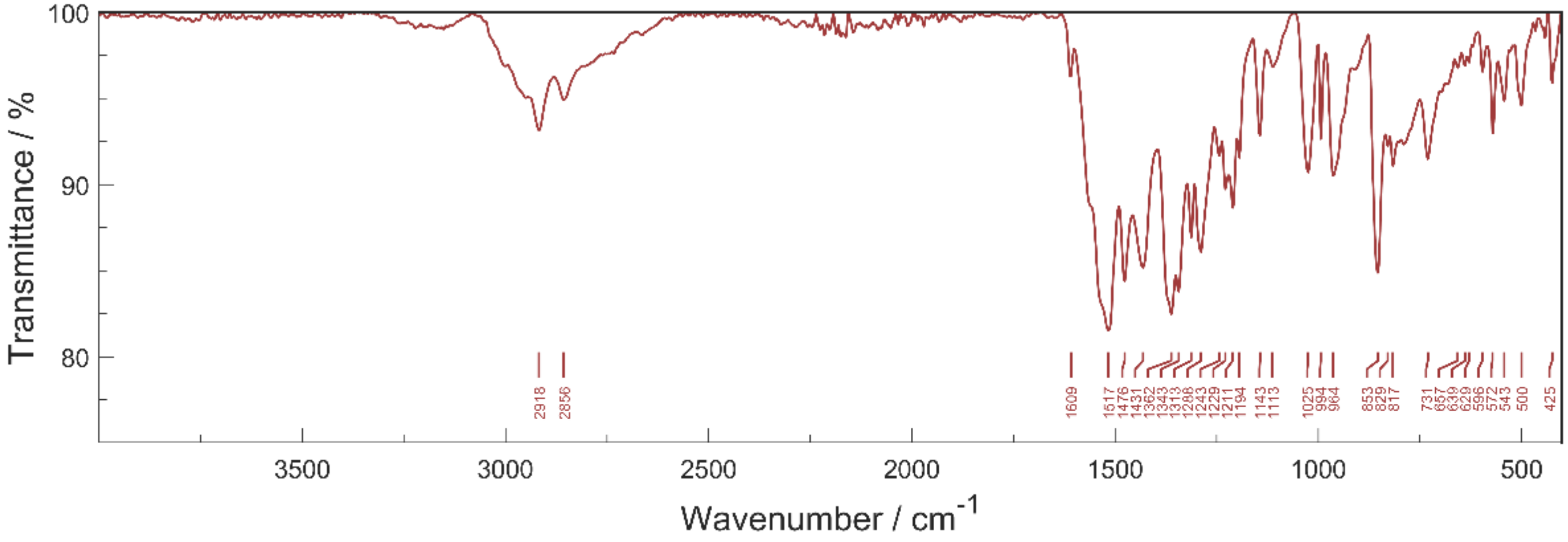

Figure S 36: ATR-IR spectrum of **4** at 298 K.

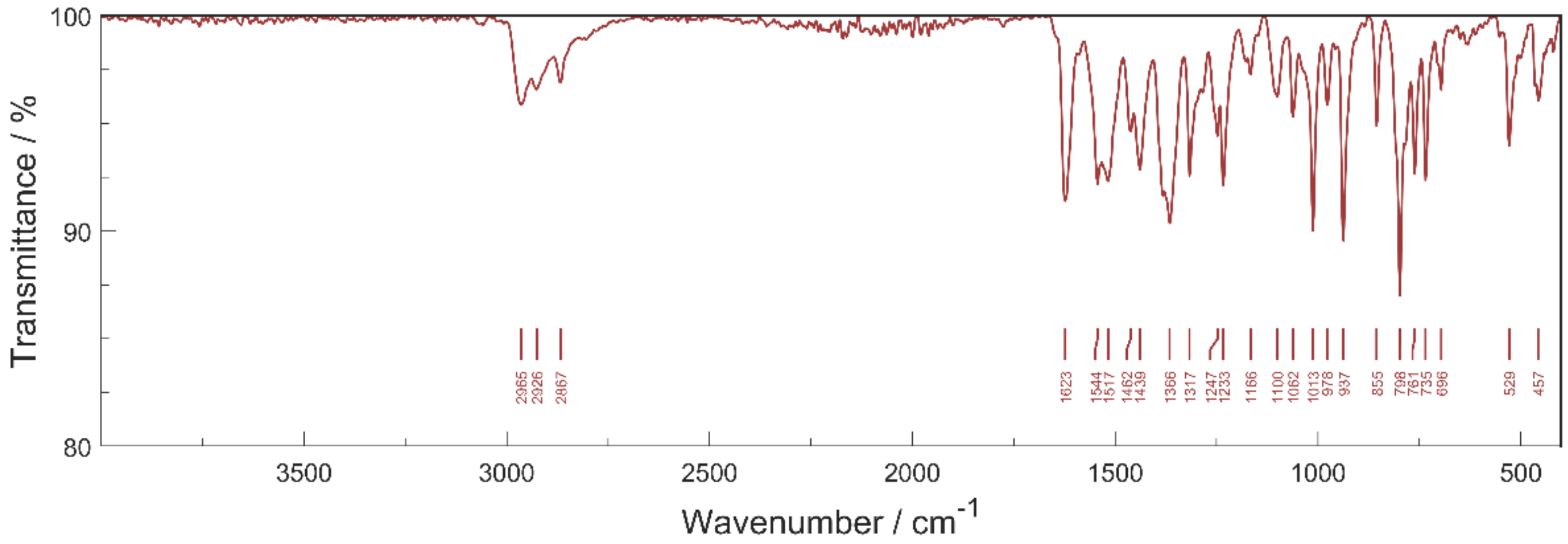

Figure S 37: ATR-IR spectrum of **5** at 298 K.

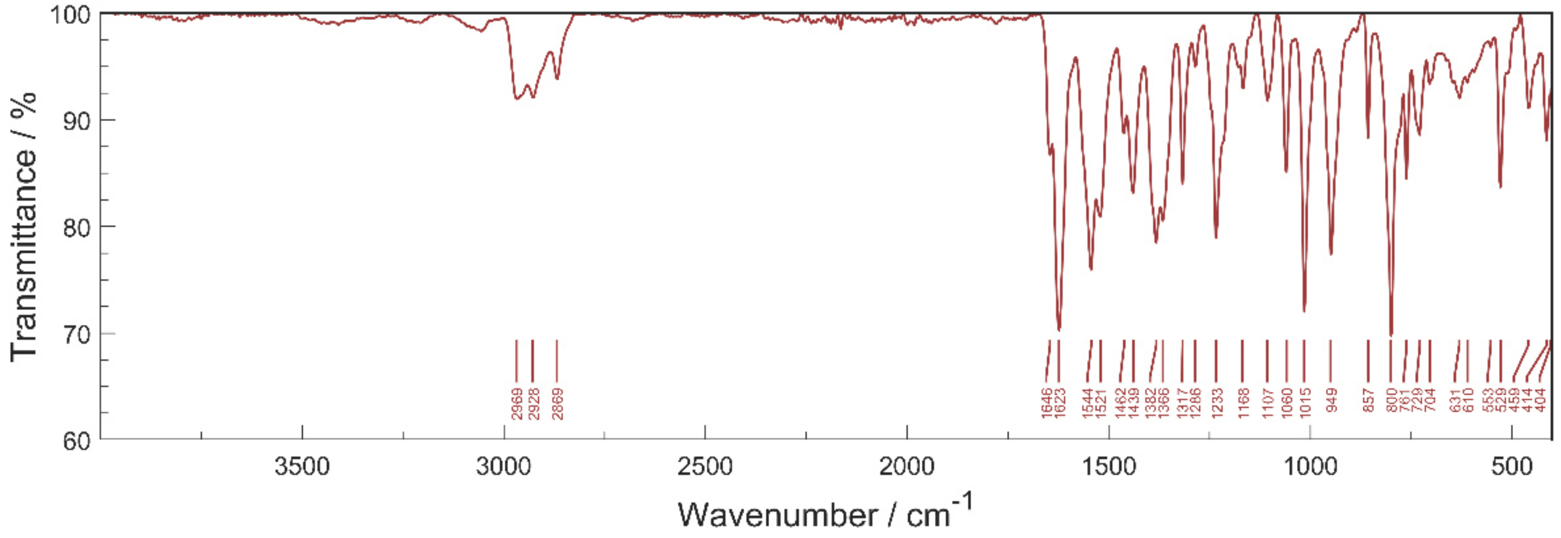

Figure S 38: ATR-IR spectrum of **6** at 298 K.

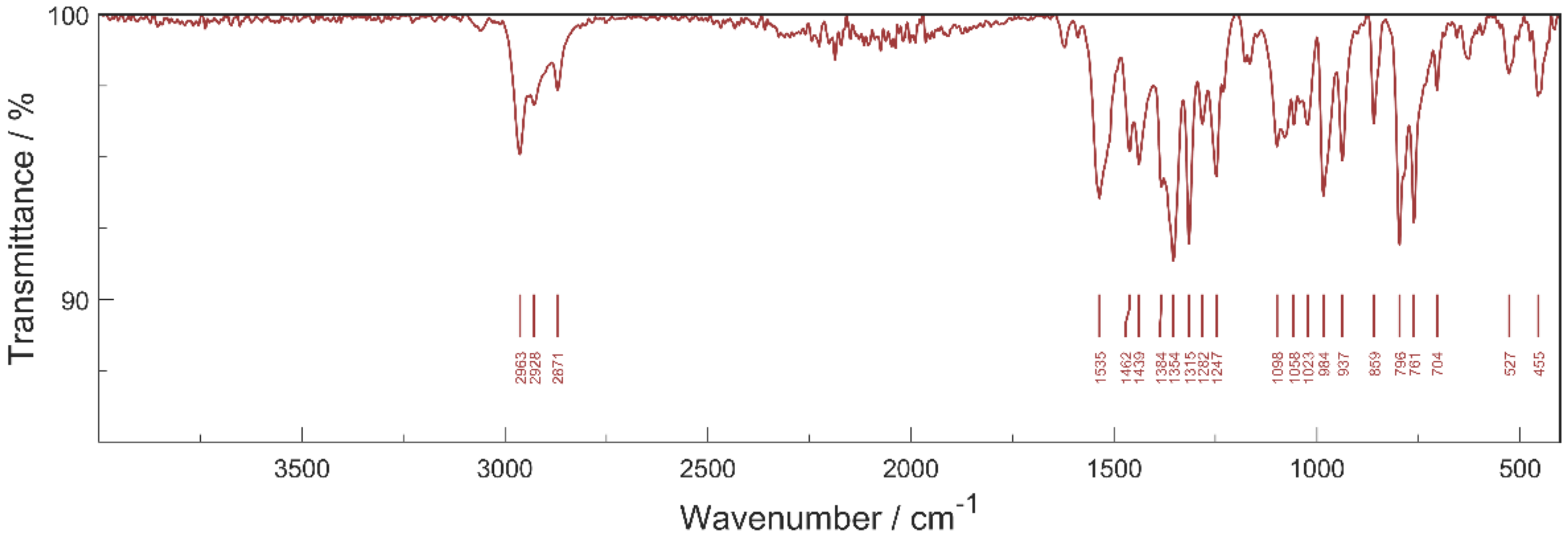

Figure S 39: ATR-IR spectrum of **7** at 298 K.

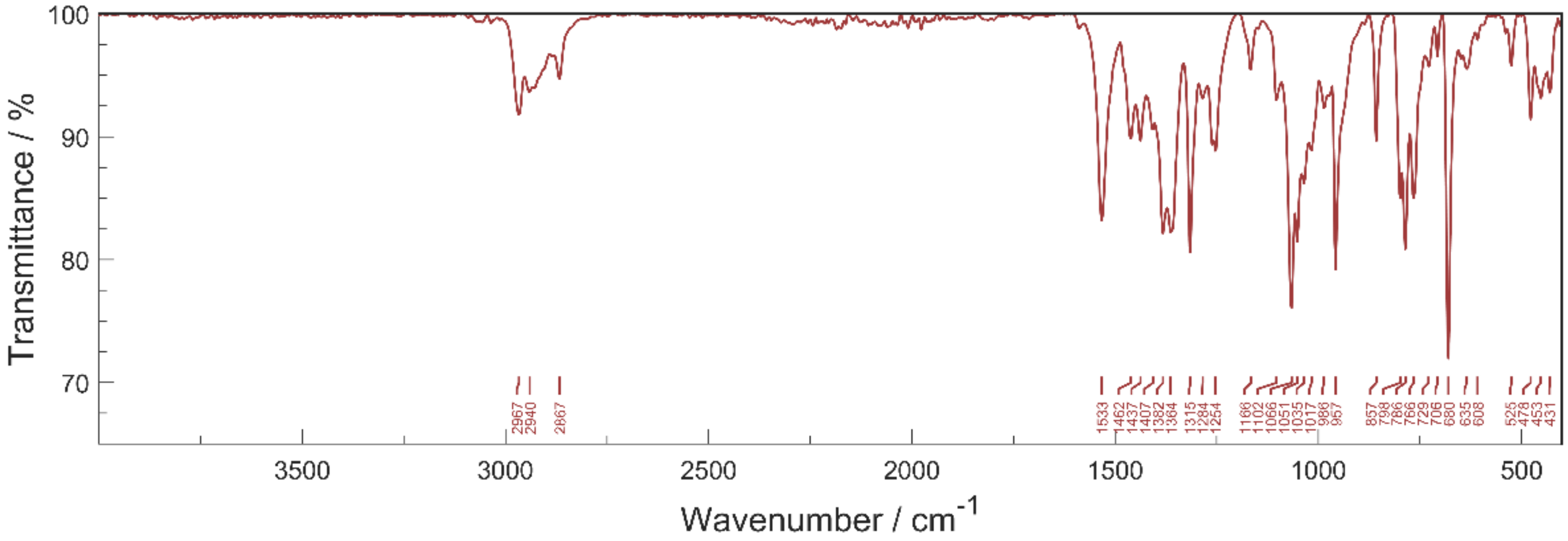

Figure S 40: ATR-IR spectrum of **8** at 298 K.

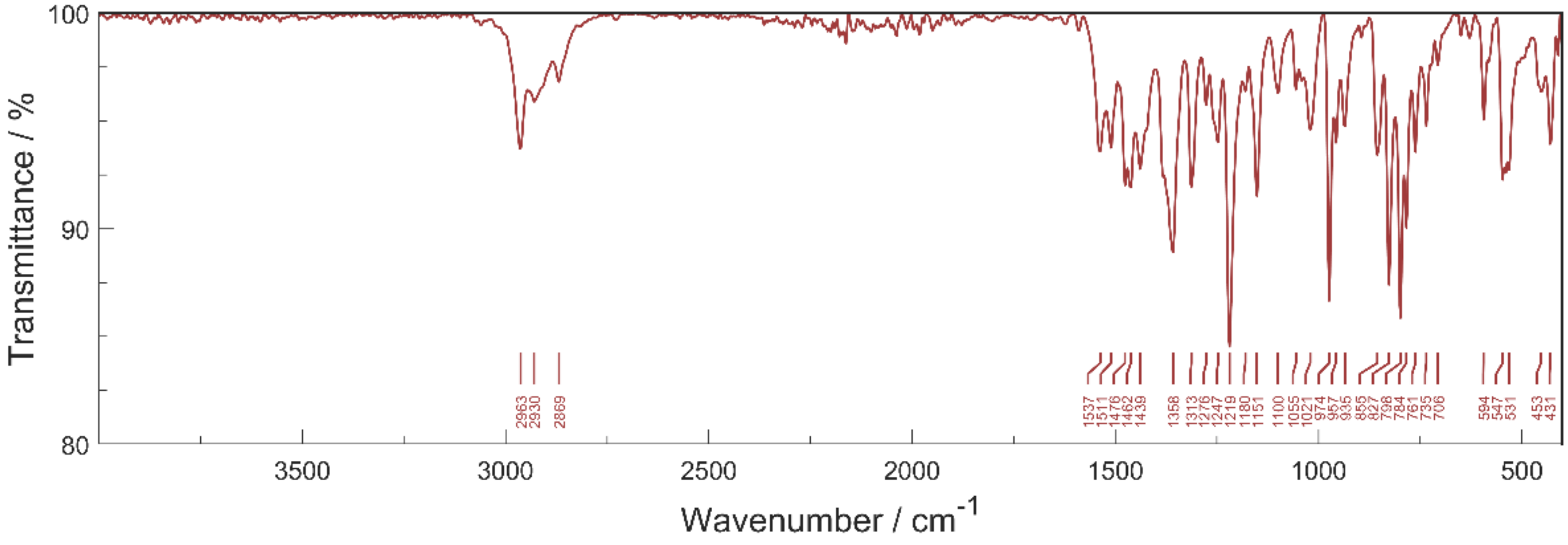

Figure S 41: ATR-IR spectrum of **9** at 298 K.

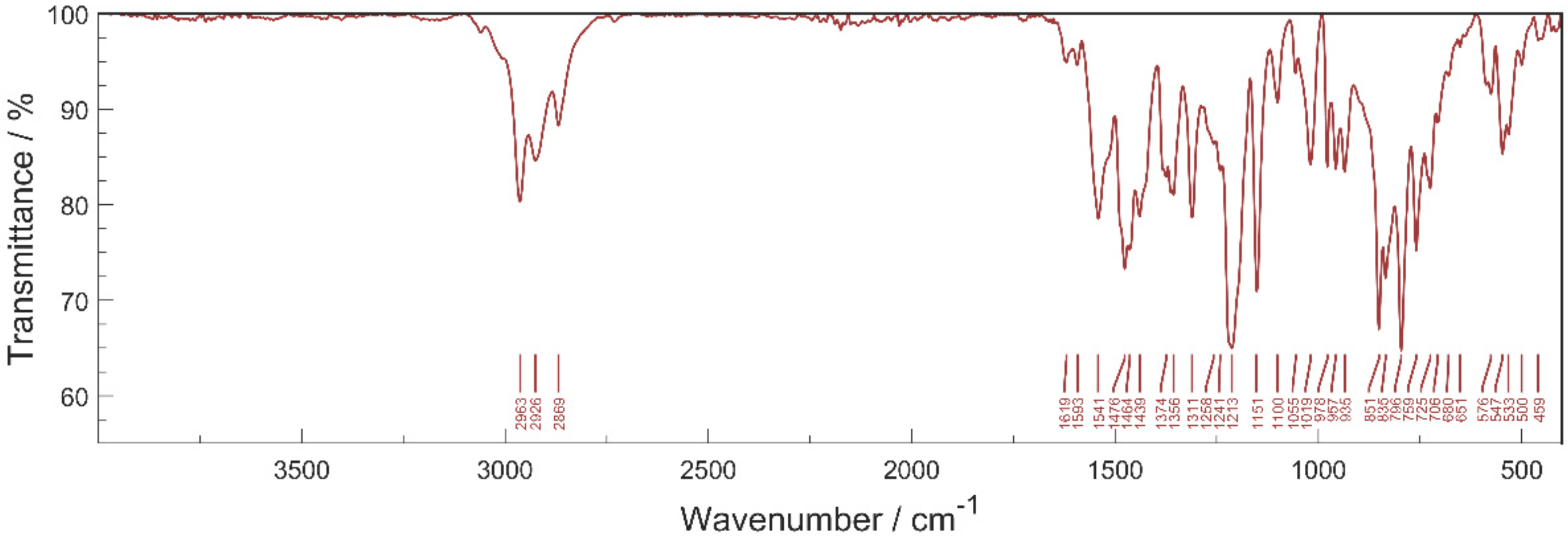

Figure S 42: ATR-IR spectrum of **10** at 298 K.

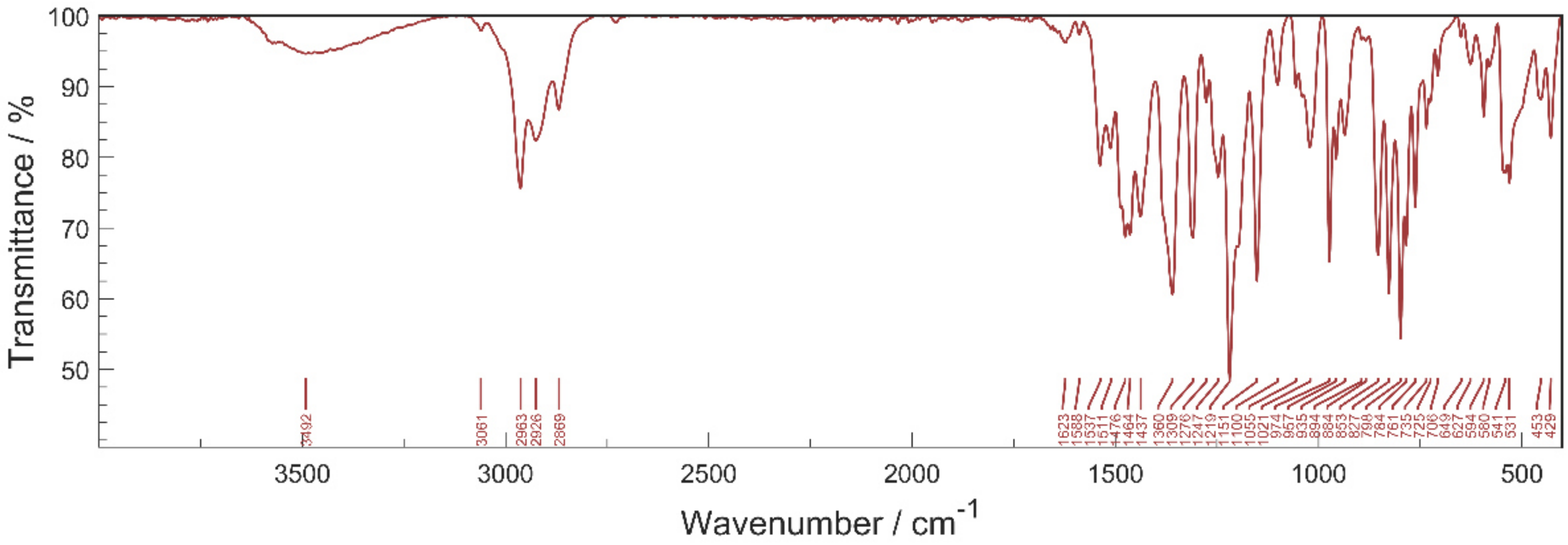

Figure S 43: ATR-IR spectrum of **11** at 298 K.

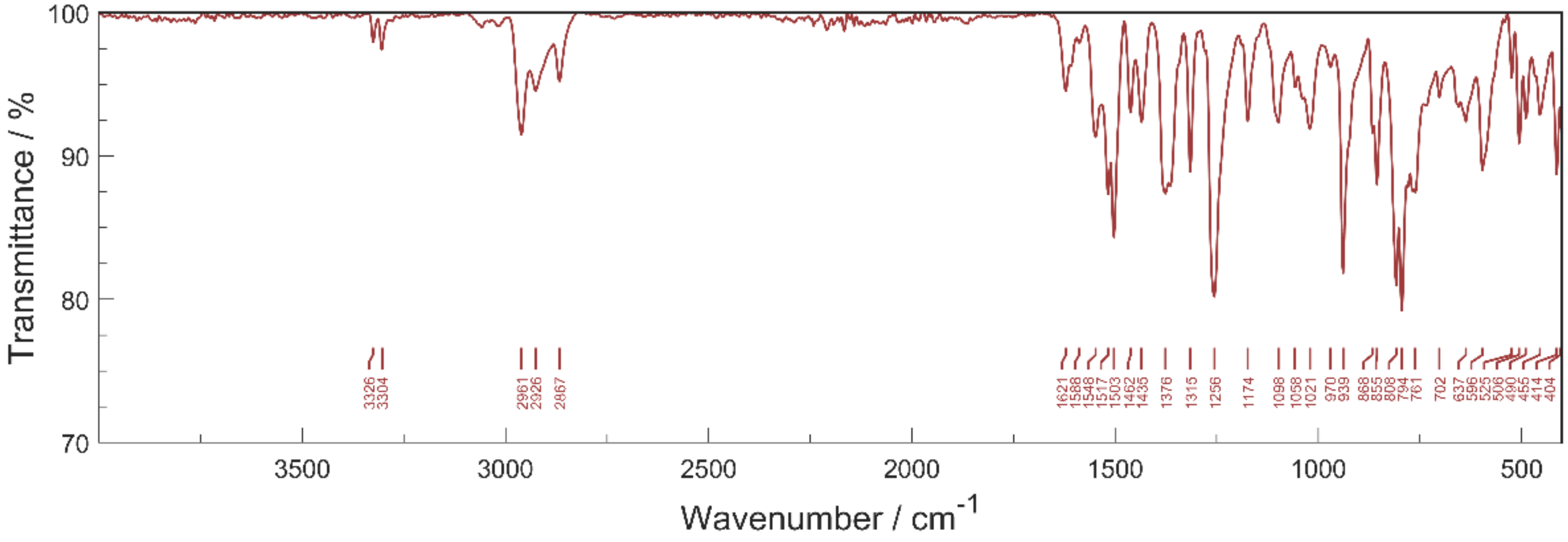

Figure S 44: ATR-IR spectrum of **12** at 298 K.

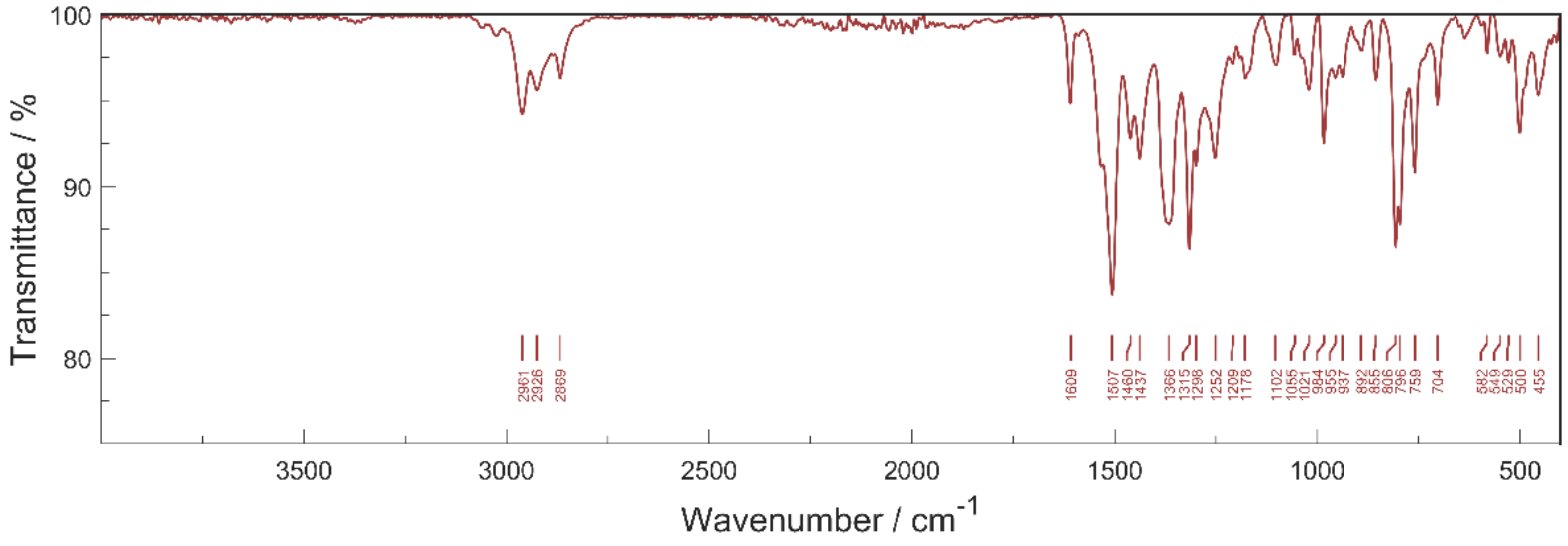

Figure S 45: ATR-IR spectrum of **13** at 298 K.

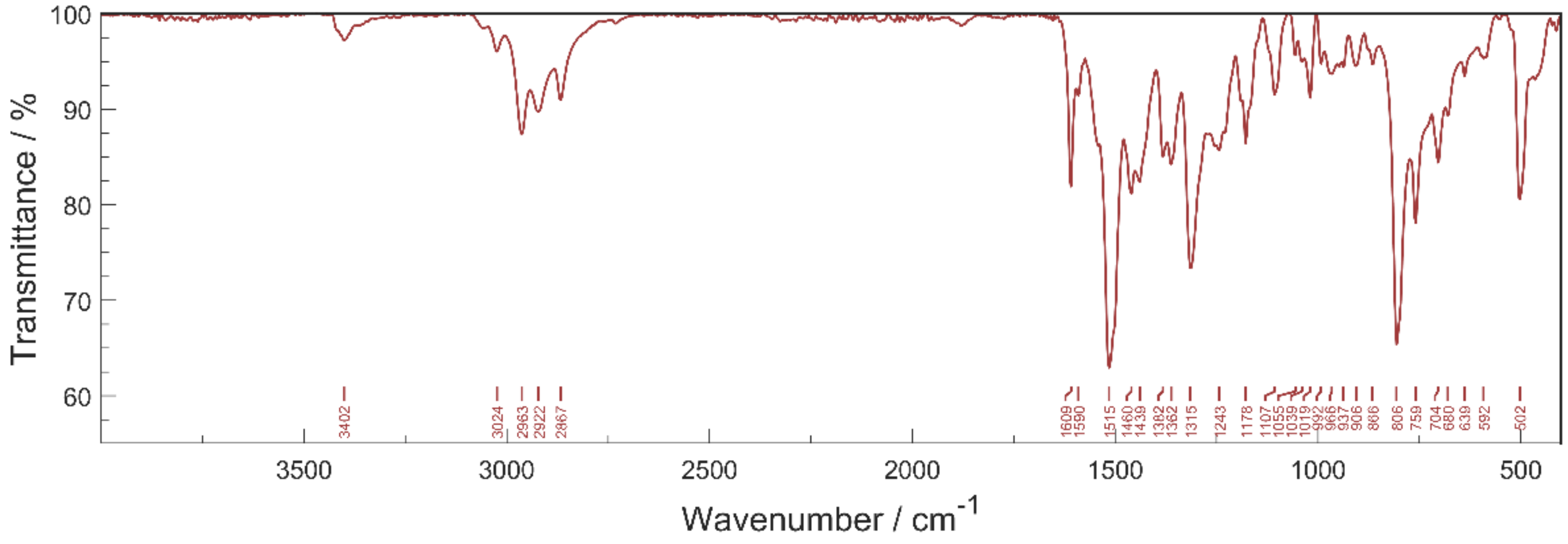

Figure S 46: ATR-IR spectrum of **14** at 298 K.

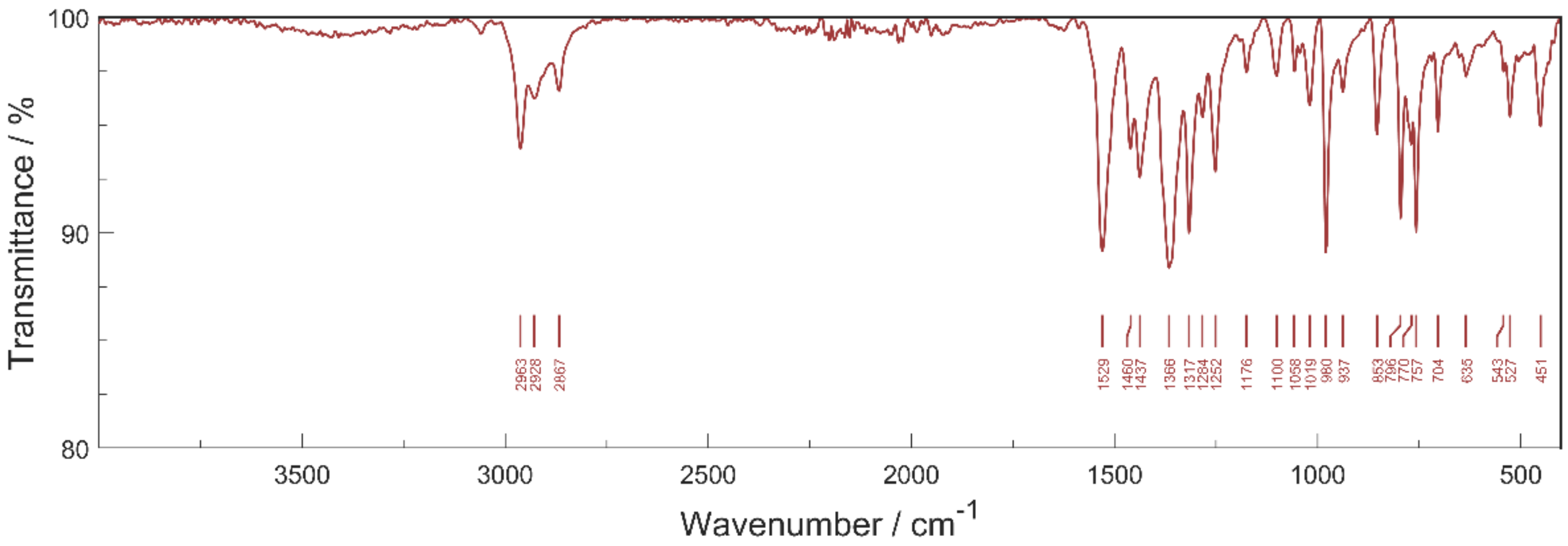

Figure S 47: ATR-IR spectrum of **15** at 298 K.

4. Crystallographic details

*Table S1: Crystallographic data for complexes **1** - **8***

	**1**	**2**	**3**	**4**	**5**	**6**	**7***	**8***
Chemical formula	$C_{29}H_{41}N_2O_1Cl_2Mo_1$	$C_{29}H_{41}N_2O_1Cl_2W_1$	$C_{23}H_{29}N_2O_1Cl_2Mo_1$	$C_{23}H_{29}N_2O_1Cl_2W_1$	$C_{36}H_{51}N_4O_1Cl_2Mo_1$ 0.75($C_4H_{10}O_1$)	$C_{36}H_{51}N_4O_1Cl_2W_1$ 0.75($C_4H_{10}O_1$)	$C_{35}H_{56}N_2O_2P_1Cl_2Mo_1$	$C_{35}H_{56}N_2O_2P_1Cl_2W_1$
M_r	600.48	688.39	516.32	604.23	778.23	866.14	734.62	822.53
Crystal system	Orthorhombic	Orthorhombic	Triclinic	Triclinic	Monoclinic	Monoclinic	Monoclinic	Monoclinic
Space group	*Pnma*	*Pnma*	*P*-1	*P*-1	$P2_1/n$	$P2_1/n$	*C*2/*c*	*C*2/*c*
a (Å)	13.7131(5)	13.6370(6)	9.0178(5)	9.0427(5)	9.987(10)	10.1394(8)	36.4916(11)	36.6413(17)
b (Å)	21.7428(7)	21.7900(9)	11.6478(6)	11.6987(7)	19.002(18)	19.0555(14)	17.9392(6)	17.9817(8)
c (Å)	9.9618(4)	10.0026(5)	12.1039(6)	12.1194(6)	22.33(2)	22.2601(14)	16.5789(5)	16.5615(7)
α (°)	90	90	71.086(2)	70.503(2)	90	90	90	90
β (°)	90	90	79.991(2)	79.578(2)	100.33(4)	99.984(2)	105.9860(10)	105.7830(10)
γ (°)	90	90	77.355(2)	77.008(2)	90	90	90	90
V (Å^3)	2970.22(19)	2972.3(2)	1166.24(11)	1169.75(11)	4168(7)	4235.8(5)	10433.3(6)	10500.5(8)
Z	4	4	2	2	4	4	8	8
Densitiy (g cm^{-3})	1.343	1.538	1.470	1.715	1.240	1.358	0.935	1.041
F(000)	1252	1380	530	594	1642	1770	3096	3352
Radiation Type	MoK_α	MoK_α	MoK_α	MoK_α	MoK_α	MoK_α	MoK_α	MoK_α
μ (mm^{-1})	0.645	4.089	0.808	5.182	0.478	2.887	0.407	2.355
Crystal size (mm)	0.35x0.23x0.10	0.45x0.44x0.40	0.33x0.28x0.19	0.35x0.31x0.10	0.05x0.03x0.02	0.12x0.10x0.07	0.25x0.22x0.14	0.01x0.03x0.02
Meas. Refl.	41865	21220	41741	56372	91204	108156	121706	113920
Indep. Refl.	3103	3003	4783	5367	7955	8704	9530	10778
Obsvd. [$I > 2\sigma(I)$]	2344	2614	4022	4774	6682	6818	8062	7377
R_{int}	0.1873	0.0766	0.0871	0.0851	0.0813	0.1168	0.0691	0.1211
R_1 [$I > 2\sigma(I)$]	0.0488	0.0378	0.0333	0.0244	0.0416	0.0431	0.0350	0.0430
$wR_2(F^2)$	0.1204	0.0908	0.0732	0.0454	0.1026	0.1101	0.0985	0.1100
GooF	1.081	1.081	1.031	1.028	1.083	1.040	1.034	1.020
$\Delta\rho_{max}$ (e Å^{-3})	0.770	0.947	0.478	0.825	0.617	0.885	0.284	1.143
$\Delta\rho_{min}$ (e Å^{-3})	-0.1318	-2.751	-0.593	-0.805	-0.418	-1.204	-0.362	-1.239
CCDC	2153141	2153140	2097750	2097753	2153144	2153143	2153145	2153142

*Due to some heavily disordered pentane molecules the SQUEEZE algorithm was applied for these structures.

Table S 2: Crystallographic data for complexes ***9*** *-* ***16***

	9	10#	11	12	13	14	15	16	17#
Chemical formula	$C_{38}H_{52}N_2O_2Cl_1Mo_1$	$C_{38}H_{52}N_2O_2Cl_1W_1$ 0.5(C2O)*	$C_{41}H_{51}N_2O_2Mo_1$	$C_{43}H_{57}N_4O_1Mo_1$	$C_{43}H_{55}N_3O_1Cl_1Mo_1$	$C_{43}H_{55}N_3O_1Cl_1W_1$	$C_{58}H_{82}N_4O_2Mo_2$ 2($C_4H_{10}O_1$)	$C_{58}H_{82}N_4O_3W_2$	$C_{58}H_{82}N_4O_2Cl_2W_2$
M_r	700.20	808.12	715.77	741.86	761.29	849.20	1278.29	1250.97	1305.87
Crystal system	Orthorhombic	Triclinic	Monoclinic	Triclinic	Monoclinic	Orthorhombic	Monoclinic	Triclinic	Triclinic
Space group	*Pbca*	P-1	$P2_1/n$	P-1	*C2/c*	$Pna2_1$	$P2_1/n$	P-1	P-1
a (Å)	27.4112(15)	9.2813(5)	8.9835(3)	10.3096(5)	40.770(3)	15.7066(12)	13.9987(4)	10.7785(5)	9.1993(13)
b (Å)	9.2982(5)	11.7303(5)	21.3628(7)	19.2282(11)	11.4904(9)	12.5428(10)	15.4997(5)	13.0902(7)	12.0391(16)
c (Å)	34.5224(19)	18.7167(10)	19.7475(7)	20.6960(11)	22.1771(19)	19.6990(13)	15.3794(6)	21.4274(12)	15.238(2)
α (°)	90	82.736(2)	90	89.220(2)	90	90	90	83.901(2)	69.443(4)
β (°)	90	81.138(2)	97.8360(10)	88.207(2)	121.174(2)	90	99.5020(10)	86.990(2)	88.744(4)
γ (°)	90	78.375(2)	90	77.200(2)	90	90	90	68.069(2)	68.883(4)
V ($Å^3$)	8798.9(8)	1962.50(17)	3754.4(2)	3998.7(4)	8888.9(13)	3880.8(5)	3291.17(19)	2788.3(3)	1463.4(4)
Z	8	2	4	4	8	4	2	2	1
Densitiy (g cm^{-3})	1.057	1.368	1.266	1.232	1.138	1.453	1.290	1.490	1.482
F(000)	2952	822	1508	1572	3208	1732	1352	1260	656
Radiation Type	MoK_α	MoK_α	MoK_α	MoK_α	MoK_α	MoK_α	MoK_α	MoK_α	MoK_α
μ (mm^{-1})	0.387	3.045	0.388	0.364	0.387	3.082	0.510	4.167	4.060
Crystal size (mm)	0.38x0.10x0.08	0.25x0.21x0.10	0.28x0.27x0.22	0.25x0.24x0.23	0.15x0.13x0.09	0.11x0.02x0.015	0.25x0.10x0.09	0.15x0.12x0.01	0.13x0.09x0.08
Meas. Refl.	139602	14857	56846	231289	57305	30557	62561	151623	7434
Indep. Refl.	7738	14857	7691	16479	7821	6609	6459	12814	7434
Obsvd. [$I > 2\sigma(I)$]	6191	13780	6253	13271	5810	4777	5686	9613	6870
R_{int}	0.1516	0.0440	0.0719	0.0877	0.0933	0.1222	0.0560	0.1524	0.0646
R_1 [$I > 2\sigma(I)$]	0.0777	0.0360	0.0354	0.0379	0.0660	0.0476	0.0284	0.0420	0.0670
$wR_2(F^2)$	0.1744	0.0809	0.0770	0.0923	0.1824	0.1181	0.0642	0.1054	0.1667
GooF	1.134	1.071	1.043	1.051	1.027	1.002	1.050	1.081	1.101
$\Delta\rho_{max}$ (e $Å^{-3}$)	1.010	1.262	0.290	0.487	3.522	1.562	0.324	1.706	4.932
$\Delta\rho_{min}$ (e $Å^{-3}$)	-1.495	-0.652	-0.552	-0.763	-0.680	-0.946	-0.436	-1.846	-3.275
CCDC	2153309	2156408	2099970	2236155	2153308	2156633	2225297	2225298	2231867

# The structure was refined as a two component twin.

* Due to a strong symmetry disorder, the protons on the partially occupied diethyl ether could not be resolved.

Table S3: Selected bond lengths and angles

	1	2	3	4	5	6	7	8	9	10
M1 – M1	-	-	-	-	-	-	-	-	-	-
M1 – N1	2.090(3)	2.100(3)	2.097(2)	2.094(2)	2.159(3)	2.113(4)	2.1070(19)	2.116(4)	2.076(5)	2.138(4)
M1 – N2	-	-	2.089(2)	2.088(3)	2.099(3)	2.149(5)	2.1306(18)	2.1244)	2.150(5)	2.069(4)
M1 – O10	1.640(4)	1.693(4)	1.657(2)	1.681(2)	1.681(3)	1.691(4)	1.6724(17)	1.706(3)	1.666(4)	1.688(3)
M1 – O11	-	-	-	-	-	-	-	-	-	-
M1 – Cl1/O40/N40	2.3390(10)	2.3385(13)	2.3309(7)	2.3343()	2.453(3)	2.4594(16)	2.4945(6)	2.4946(13)	1.932(4)	1.938(3)
M1 – Cl2/O50	-	-	2.3520(8)	2.3502(8)	2.383(2)	2.3753(14)	2.3946(6)	2.3935(13)	2.3278(15)	2.3301(13)
M1 – N30/O30	-	-	-	-	2.256(3)	2.237(5)	2.1117(15)	2.104(3)	-	-
N1 – M1 – N2	87.10(14)	86.38(19)	86.47(9)	86.15(10)	87.02(9)	87.6(16)	88.47(7)	89.14(159	87.51(17)	87.57(15)
X* – M1 – X*	83.71(5)	83.86(7)	84.00(3)	83.89(3)	91.37(4)	90.18(6)	89.48(2)	88.54(5)	85.56(11)	85.26(11)
N1 – M1 – X*	148.98(8)	149.23(11)	153.03(6)	152.68(7)	175.78(7)	172.12(18)	170.39(7)	169.30(14)	139.67(14)	156.09(15)
N2 – M1 – X*	148.98(8)	149.23(11)	145.19(7)	145.61(7)	172.34(9)	174.73(13)	173.61(5)	172.59(11)	157.87(18)	141.90(11)
N1 – M1 – X*	86.41(8)	86.82(10)	87.61(6)	86.38(7)	84.82(7)	89.75(13)	87.54(6)	86.91(11)	85.96(17)	86.80(11)
N2 – M1 – X*	86.41(8)	86.82(10)	85.96(6)	87.61(7)	97.42(9)	84.90(13)	91.66(6)	84.16(11)	85.82(13)	84.88(15)
N1 – M1 – O10	102.92(11)	103.27(15)	101.14(9)	101.81(10)	88.85(10	101.43(18)	98.96(8)	98.57(16)	106.07(19)	97.95(16)
N2 – M1 – O10	102.92(11)	103.27(15)	104.09(10)	104.62(10)	102.60(11)	89.89(18)	91.28(8)	91.46(16)	96.71(19)	105.51(16)
O10 – M1 – X*	108.10(10)	107.50(12)	110.71(8)	109.77(8)	95.26(8)	95.31(14)	95.01(6)	95.69(13)	114.20(15)	112.59(13)
O10 – M1 – X*	108.10(10)	107.50(12)	105.83(8)	105.50(8)	165.62(7)	167.43(14)	171.97(6)	172.95(12)	105.42(18)	105.93(16)
τ_5/τ_4'	0.00	0.00	0.13	0.12	-	-	-	-	0.30	0.24

*X corresponds to the coordinating atom being trans to the BDI nitrogen atoms and refer to Cl1, Cl2, O11, O10, N30, N40, N50, O30, O40, O50

[#] The tungsten center in complex 3 is octahedrally coordinated by six ligands.

	11	12	13	14	15	16**	17
M1 – M1	-	-	-	-	3.869(1)	2.6223(3)	3.1742(14)
M1 – N1	2.1159(19)	2.118(3)	2.179(4)	2.078(14)	2.1110(16)	2.143(5)	2.330(13)
M1 – N2	2.1107(18)	2.2098(19)	2.093(4)	2.134(5)	2.1184(16)	2.121(5)/2.184(5)	-
M1 – O10/N10	1.6765(16)	1.6801(18)	1.662(3)	1.669(9)	1.6585(14)	1.704(4)/1.750(5)	1.801(10)/2.220(11)
M1 – O11	-	-	-	-	-	1.976(4)/1.949(4)	-
M1 – O12	-	-	-	-	-	1.916(4)/1.960(4)	-
M1 – Cl1/O40/C61	1.9390(16)	1.984(2)	2.3959(12)	2.396(4)	2.4648(5)	2.159(6)	2.356(4)
M1 – Cl2/O50/N40	1.9356(16)	2.006(2)	1.995(4)	1.984(14)	2.4841(5)	-	1.764(12)
M1 – N30/O30/C2	-	-	-	-	-	-	2.132(13)
N1 – M1 – N2	85.97(7)	84.40(7)	88.10(14)	88.9(7)	85.64(6)	85.11(18)	74.9(5)**
X* – M1 – X*	92.12(7)	90.24(9)	86.12(11)	84.4(4)	77.136(18)	89.72(16)	76.2(5)
N1 – M1 – X*	147.74(7)	136.56(9)	177.83(11)	130.4(5)	146.98(5)	135.47(19)/149.49(18)	157.5(5)
N2 – M1 – X*	149.56(7)	163.58(8)	124.42(15)	174.8(4)	150.47(5)	150.26(18)/123.6(2)	152.3(4)**
N1 – M1 – X*	82.59(7)	84.40(7)	95.03(15)	89.3(4)	90.56(5)	83.65(17)/88.38(17)	77.9(3) / 82.3(4)
N2 – M1 – X*	82.73(9)	84.71(9)	89.73(10)	93.1(5)	90.33(4)	79.28(17)/79.7(2)	96.6(5) / 94.4(5)
N1 – M1 – O10	101.94(8)	111.25(8)	87.60(15)	110.3(5)	106.32(7)	105.7(2)/98.5(2)	157.5(5)
N2 – M1 – O10	100.79(8)	95.82(8)	116.72(15)	91.4(5)	104.80(7)	98.6(2)/109.5(2)	96.6(5)
O10 – M1 – X*	109.81(8)	111.63(10)	93.46(12)	93.8(4)	106.39(5)	110.97(18)/126.5(2)	106.(11)<br>7(3)
O10 – M1 – X*	109.13(8)	100.57(9)	118.85(16)	119.2(5)	104.37(5)	117.70(19)/107.3(2)	-
τ_5/τ_4'	0.03	0.45	0.89	0.74	0.06	0.25 / 0.43	-

*X corresponds to the coordinating atom being trans to the BDI nitrogen atoms and refer to Cl1, Cl2, O11, O10, C100, N40, N50, O40, O41, O50

** N2 is C2 in this case

**first values belong to the BDI side, second to the MAD side.

5. Cyclic Voltammetry

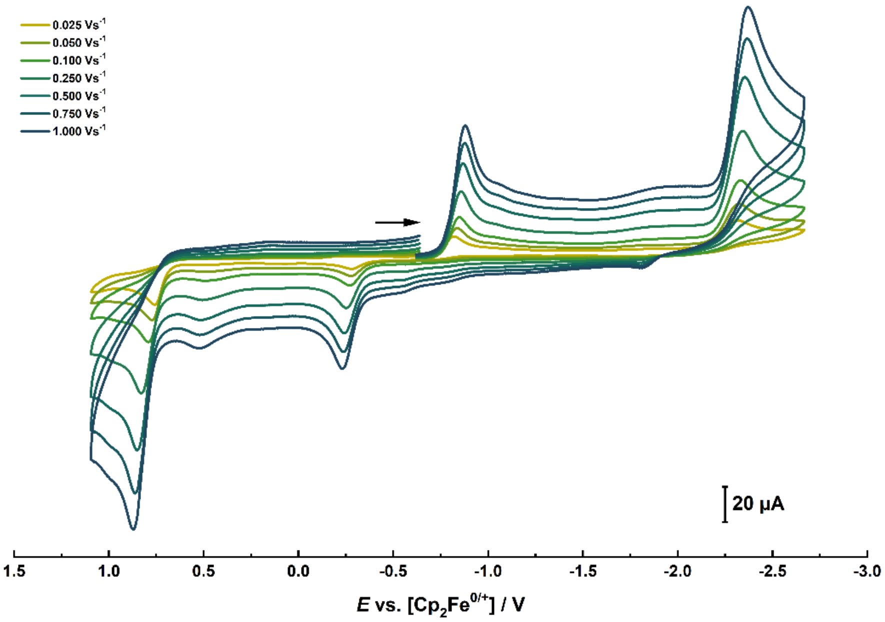

Figure S 48: Full CV of complex 1 in a 0.2 M NBu_4PF_6 MeCN solution at different scan rates between 25 – 1000 mV s^{-1}.

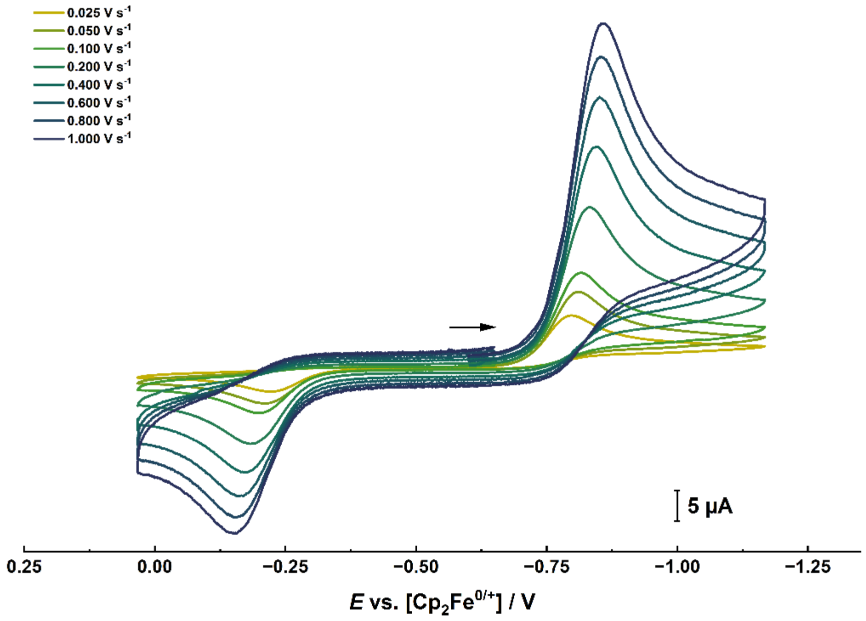

Figure S 49: Isolated measurement of the first reduction process in complex **1** in a 0.2 M NBu_4PF_6 MeCN solution at different scan rates.

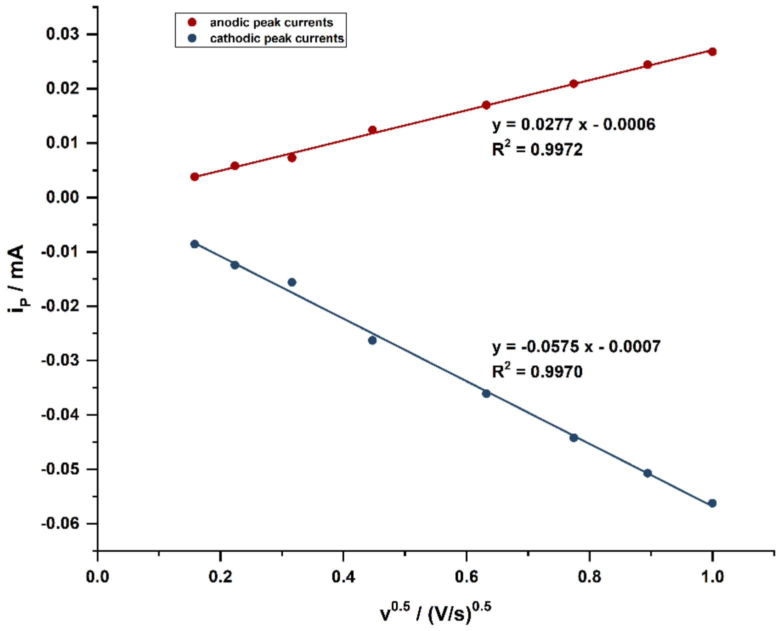

Figure S 50: Rendles-Sevcik-plot of the first reduction of complex **1** measured in a 0.2 M NBu_4PF_6 MeCN solution at different scan rates.

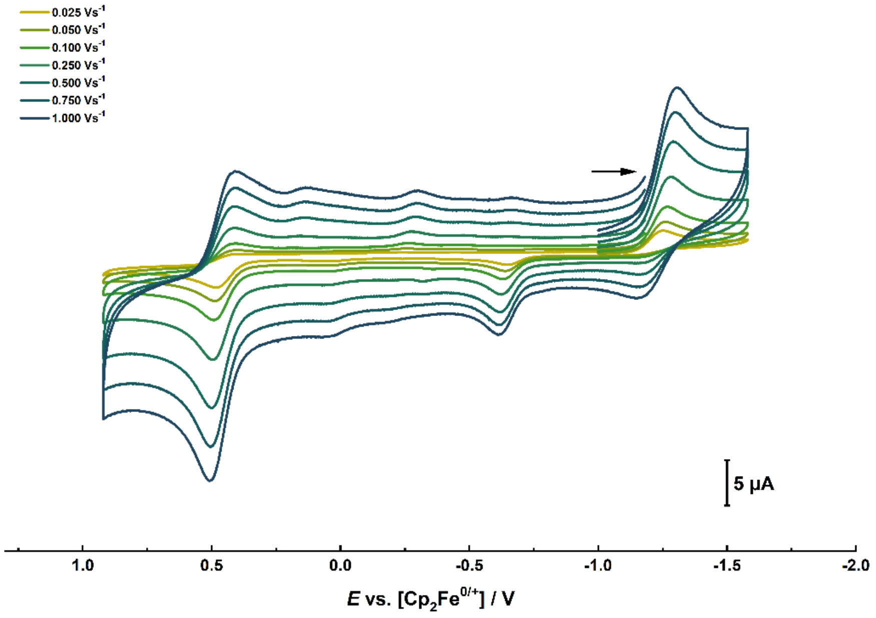

Figure S 51: Full CV of complex **2** in a 0.2 M NBu_4PF_6 MeCN solution at different scan rates between 25 – 1000 mV s^{-1}.

6. EPR spectra

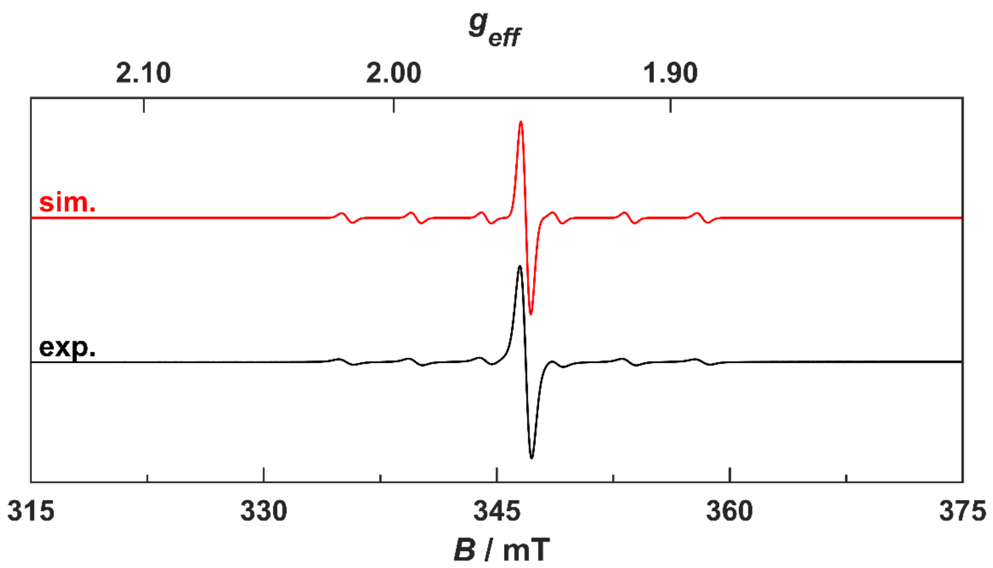

*Figure S 52: EPR spectrum of **5** in toluene at 298 K. Simulated Parameters: g_{iso} = 1.9511, a_{iso} = 124 MHz.*

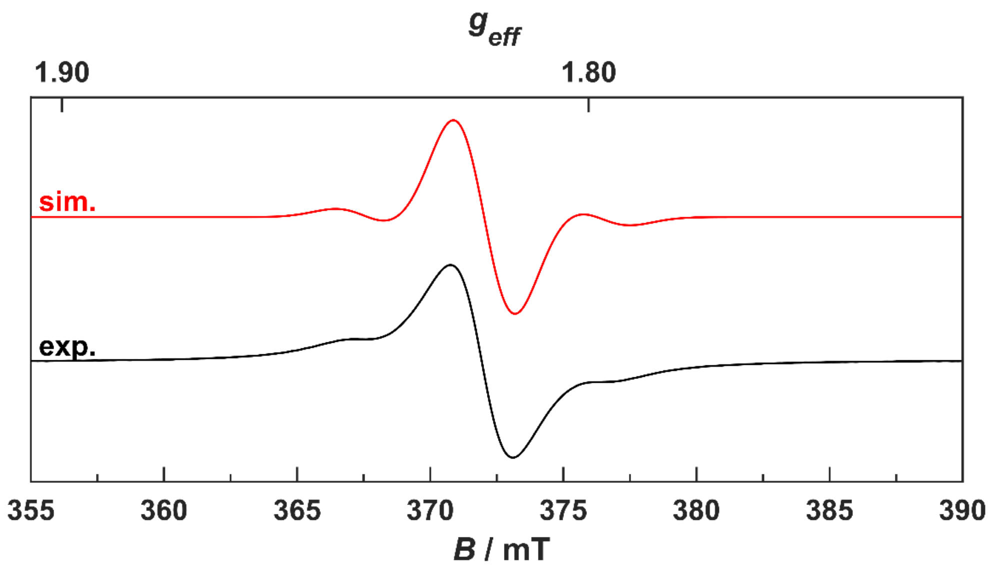

*Figure S 53: EPR spectrum of **6** in toluene at 298 K. Simulated Parameters: g_{iso} = 1.819, a_{iso} = 222 MHz.*

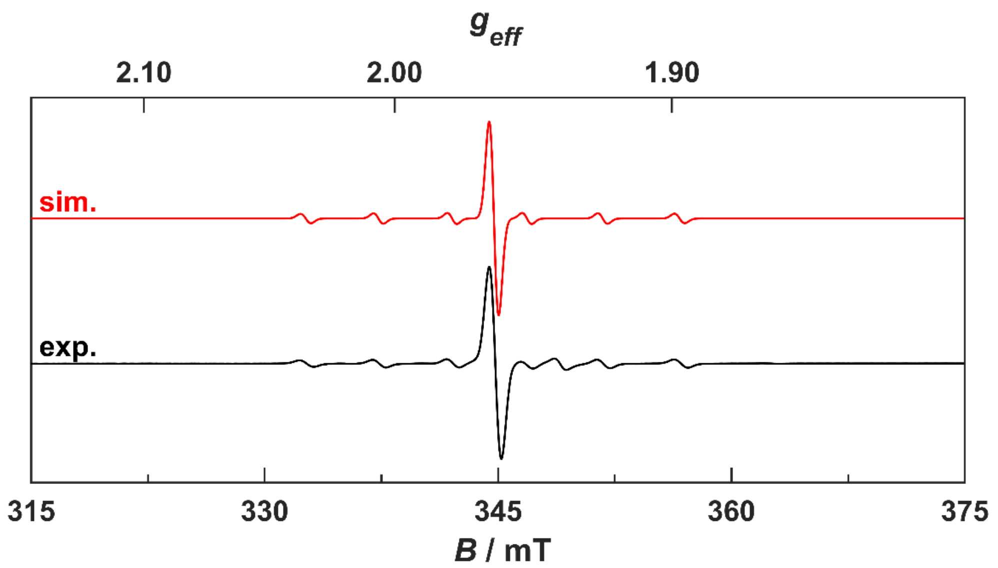

Figure S 54: EPR spectrum of **7** in toluene at 298 K. Simulated Parameters: g_{iso} = 1.963, a_{iso} = 131 MHz.

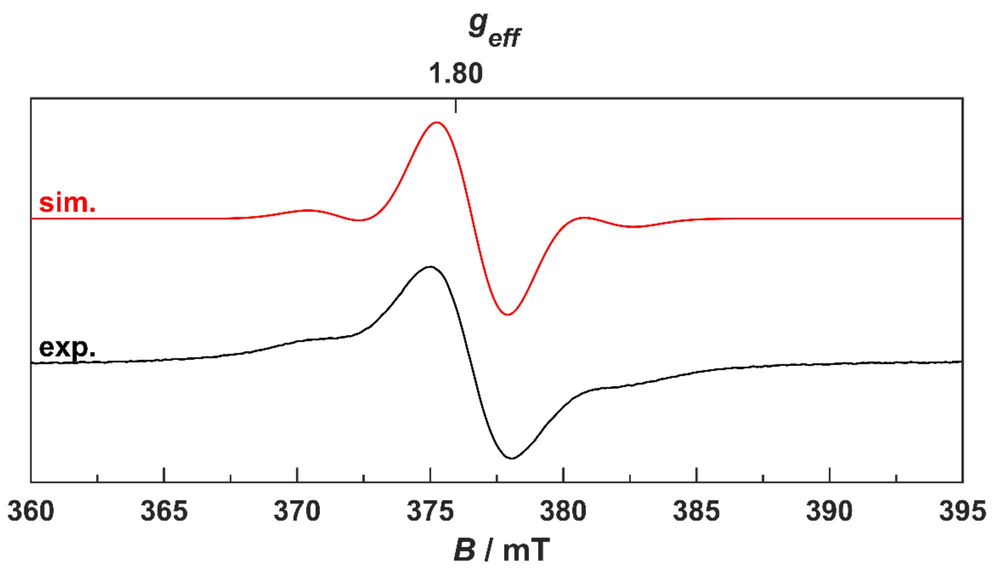

*Figure S 55: EPR spectrum of **8** in toluene at 298 K. Simulated Parameters: g_{iso} = 1.797, a_{iso} = 240 MHz.*

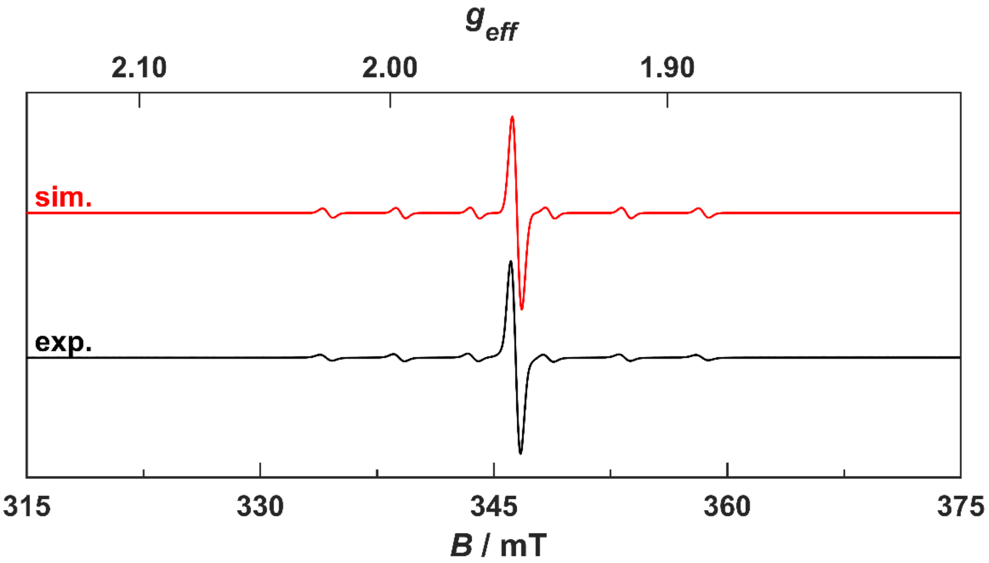

Figure S 56: EPR spectrum of **9** in toluene at 298 K. Simulated Parameters: g_{iso} = 1.953, a_{iso} = 131 MHz.

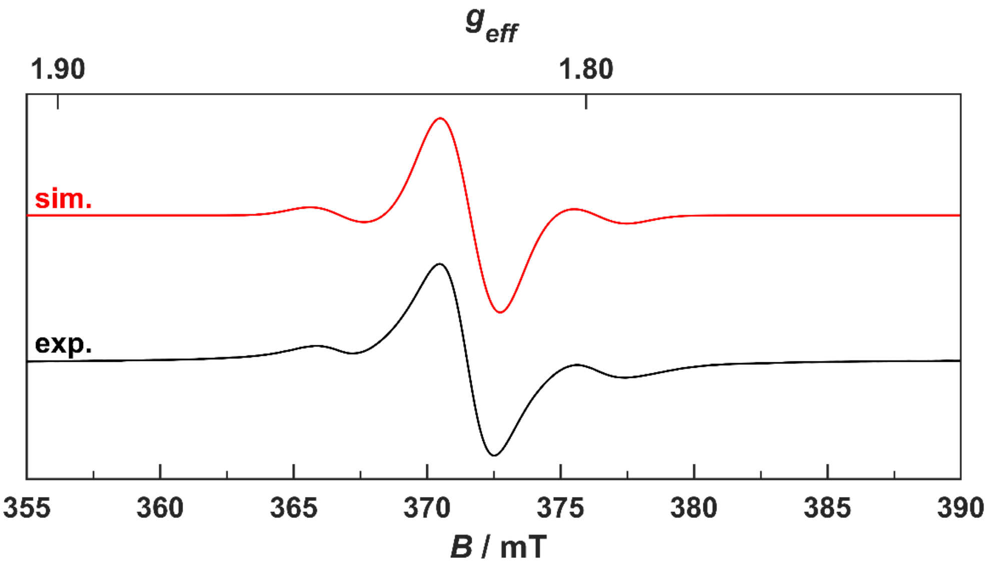

Figure S 57: EPR spectrum of **10** in toluene at 298 K. Simulated Parameters: g_{iso} = 1.821, a_{iso} = 245 MHz.

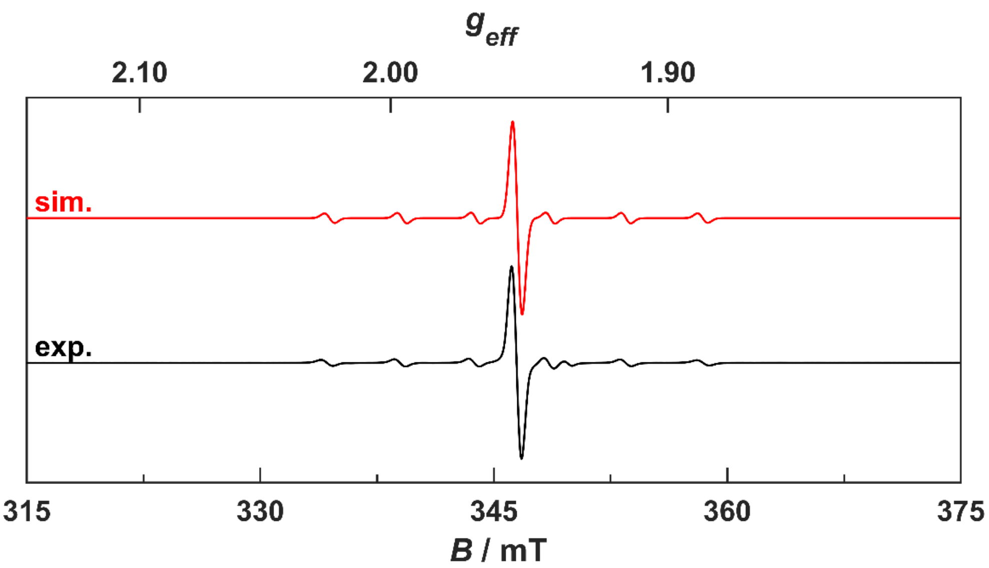

Figure S 58: EPR spectrum of **11** in toluene at 298 K. Simulated Parameters: g_{iso} = 1.953, a_{iso} = 130 MHz.

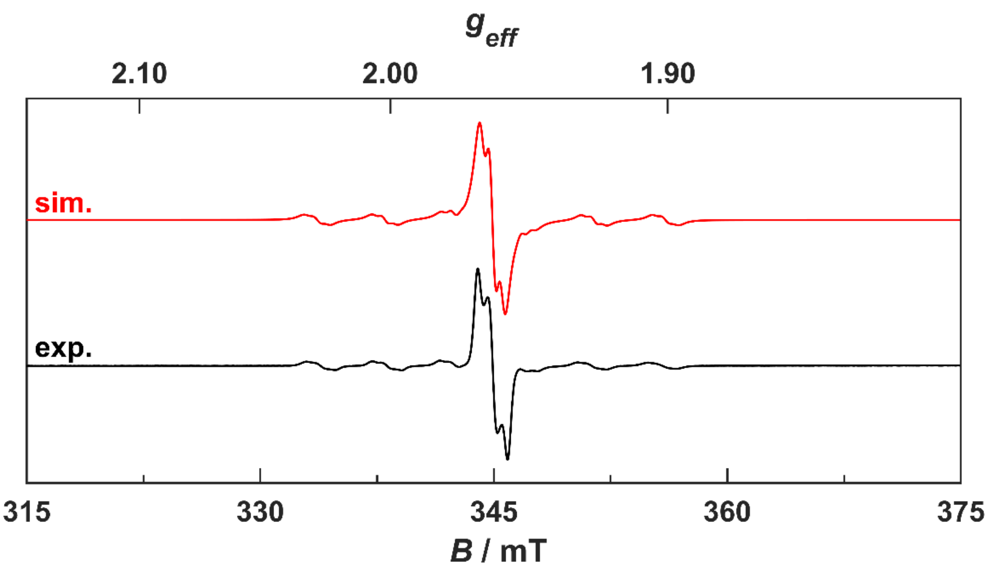

Figure S 59: EPR spectrum of **12** in toluene at 298 K. Simulated Parameters: g_{iso} = 1.962, a_{iso}(Mo) = 122 MHz, a_{iso}(H) = 17.2 MHz.

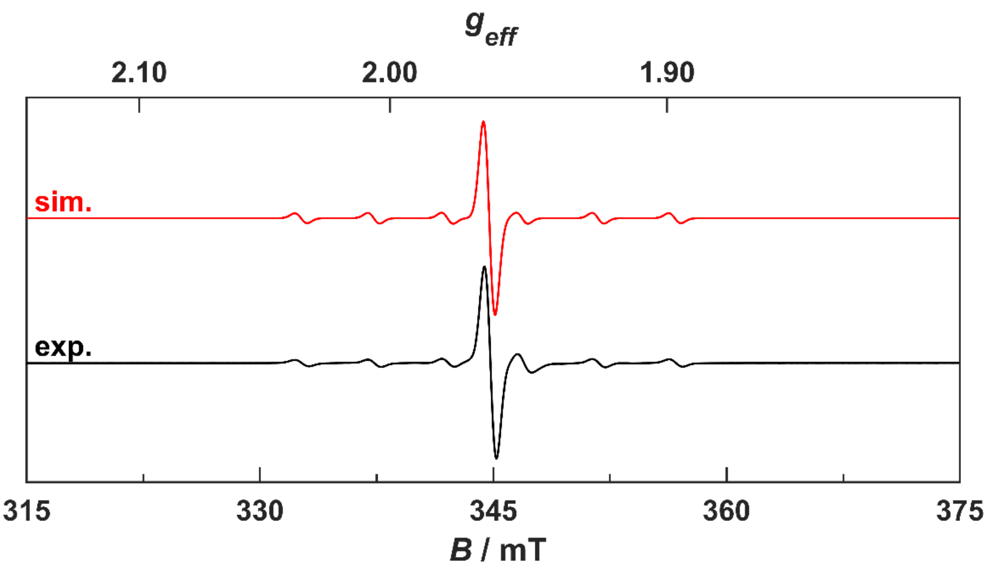

*Figure S 60: EPR spectrum of **13** in toluene at 298 K. Simulated Parameters: g_{iso} = 1.963, a_{iso}= 131 MHz.*

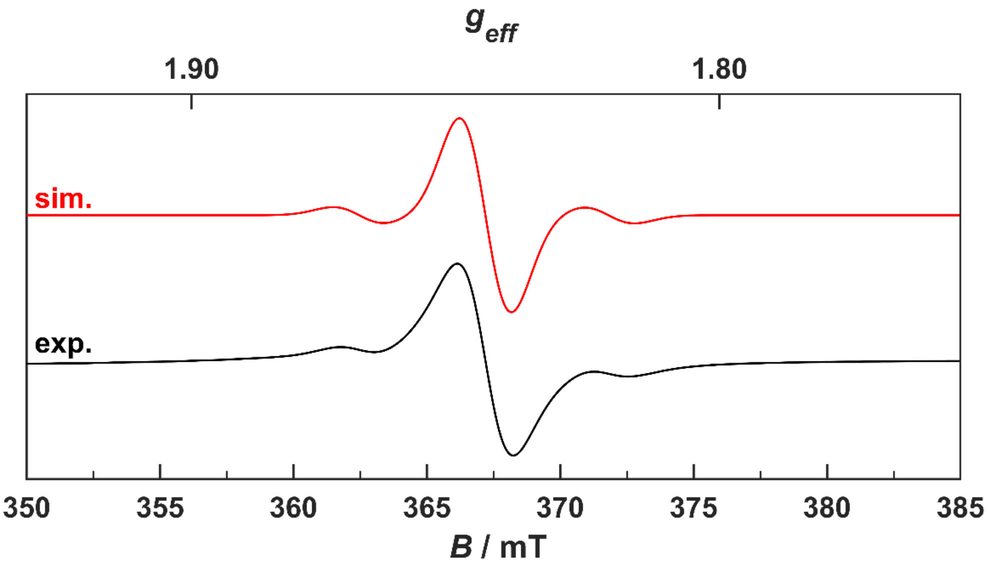

Figure S 61: EPR spectrum of **14** in toluene at 298 K. Simulated Parameters: g_{iso} = 1.843, a_{iso} = 241 MHz.
